# DABCO- and DBU-promoted one-pot reaction of *N*-sulfonyl ketimines with Morita–Baylis–Hillman carbonates: a sequential approach to (2-hydroxyaryl)nicotinate derivatives

**DOI:** 10.3762/bjoc.14.254

**Published:** 2018-11-02

**Authors:** Soumitra Guin, Raman Gupta, Debashis Majee, Sampak Samanta

**Affiliations:** 1 Discipline of Chemistry, Indian Institute of Technology Indore, Simrol, Indore, 453552, Madhya Pradesh, India

**Keywords:** MBH carbonates, metal-free, *N*-sulfonyl ketimines, one-pot sequential, pyridines

## Abstract

An intriguing DABCO-catalyzed and DBU-promoted one-pot synthesis of an important class of (2-hydroxyaryl)pyridine derivatives bearing a carboxylate or a nitrile group suitably placed at C3 position of the aza-ring has been achieved in acceptable chemical yields with a broad functional group tolerance. This sequential C–C/C–N bond making process proceeds through a regioselective allylic alkylation/aza-Michael reaction between MBH carbonates derived from an acrylate/acrylonitrile and *N*-sulfonyl ketimines as C,N-binucleophiles catalyzed by DABCO, followed by elimination of SO_2_ under the influence of base and subsequent aromatization in an open atmosphere.

## Introduction

**Figure 1:**
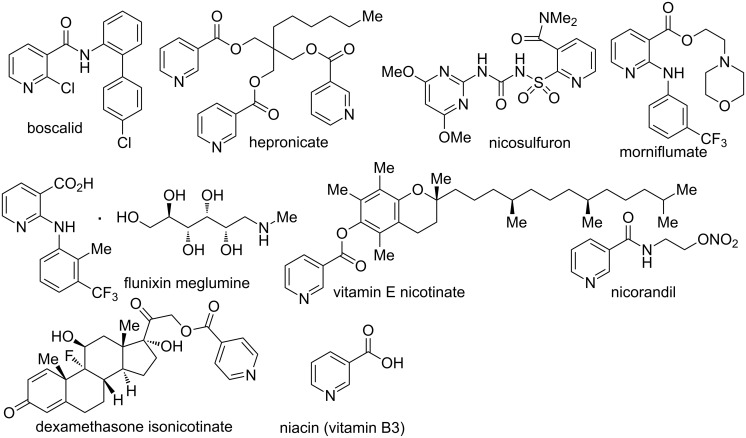
Drugs and agrochemicals having a nicotinic acid derivative.

Substituted pyridines are one of the most fascinating classes of heterocyclic molecules which are present in many biologically active natural and synthetic products [1–3]. In addition, several pyridine scaffolds have been used in agro-chemical products [4], medicines [5], chelating agents in transition metal complexes [6–7], and material science [8–9]. Pyridine derivatives such as vitamins B6, B3 (niacin), and nicotinamide adenine dinucleotide (NAD) play a significant role in the metabolism process of living organisms. Consequently, many contributions have been made by synthetic chemists to search for new method for the efficient synthesis and functionalization of pyridines. Among the most significant techniques are condensation reactions between reactive carbonyls and amines (namely Hantzsch [10] and Chichibabin pyridine synthesis [11–12]), transition metal salts-catalyzed [4 + 2] [13–18], [2 + 2 + 2] [19–26], and [3 + 3] cyclization reactions [27], multicomponent reactions [28–33], and direct C–H functionalizations on the pyridine rings [34–36]. Particularly, the fascinating construction of pyridines bearing a carboxylate or CN group at C3 position has been a lucrative target for chemists due to the pharmaceutically privileged status (Figure 1).

**Scheme 1:**
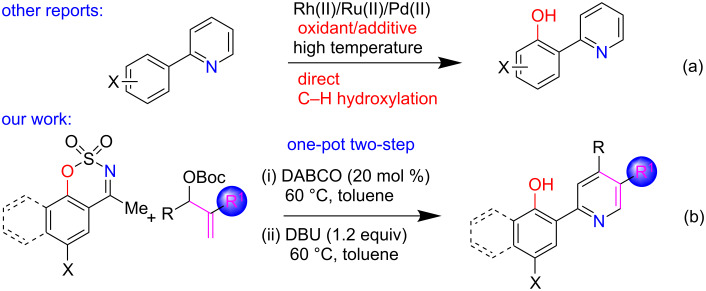
One-pot access to (2-hydroxyaryl)pyridines.

In this context, Rodriguez’s group successfully established a one-pot three-component reaction between β,γ-unsaturated α-ketoesters, 1,3-dicarbonyl compounds, and ammonium acetate promoted by acid under aerobic conditions [37–38]. Furthermore, Brønsted- and Lewis-acid-catalyzed cyclization reactions between β-enamino esters (derived from β-ketoesters and ammonium acetate) and alkynones/α,β-unsaturated carbonyls/in situ generated α,β-unsaturated ketones (from alkyl ketones) as Michael acceptors to construct a diverse set of nicotinate derivatives were developed by Bohlman–Rahtz [39], Bagley [40–41] and other groups independently [42–55]. Similarly, the synthesis of functionalized nicotinonitriles has been also greatly explored in the literature [56–57]. Even though great progress, the synthesis of (2-hydroxyaryl)pyridines bearing a CO_2_Et/CN group at C3 position remains a daunting task in synthetic organic chemistry [58–59]. A literature study reveals that a lot of research is focused on the synthesis of (2-hydroxyaryl)pyridines from 2-arylpyridines via a direct C–H hydroxylation on the aryl ring using several expensive transition metal salts (e.g., Pd(II) [60–63], Rh(III) [64–65], Ru(II) [66]) as catalysts (Scheme 1a). However, the above methods have several drawbacks such as the requirement of high temperatures or use of strong oxidants (H_2_O_2_, oxone, K_2_S_2_O_8_, TBHP, PIDA, NHPI etc.) that are not much compatible with functionality, precluding late-stage functionalization. Moreover, the scope of substitution on the pyridine ring is limited which in turn hampers the practical usage. Therefore, we are interested to devise a metal-free based general synthetic technique for the construction of substituted (2-hydroxyaryl)nicotinate/nicotinonitrile scaffolds. In recent years, our research group has been concentrated on the development of new synthetic methods for the preparation of polyfunctionalized pyridine building blocks involving cyclic sulfamidate imines as carbon nucleophiles [67–71]. In this direction, we recently documented an excellent example of an organobase promoted pot-economical approach to 4,6-disubstituted nicotinates by choosing 5-membered cyclic sulfamidate imines and MBH acetates of acrylate as coupling partners [68]. Herein, we further present a DABCO-catalyzed and DBU-promoted sequential one-pot procedure for the access to the interesting class of (2-hydroxyaryl)nicotinates/nicotinonitriles from *N*-sulfonyl ketimines and MBH adducts as useful synthons [72–73] in the presence of open atmosphere (Scheme 1b).

## Results and Discussion

We have commenced the model reaction between cyclic *N*-sulfonyl imine **1a** as interesting C,N-binucleophiles [74] and MBH carbonate of acrylate **2a** using 20 mol % of DABCO in toluene at room temperature for 14 h. This reaction produced 87% yield of allylic alkylation adduct **3a** along with a small amount of 9,10-dihydro-8*H*-benzo[*e*]pyrido[1,2-*c*]oxathiazine 6,6-dioxide **4a** in 4% yield (entry 1, Table 1).

**Table 1:** Optimization reaction conditions.

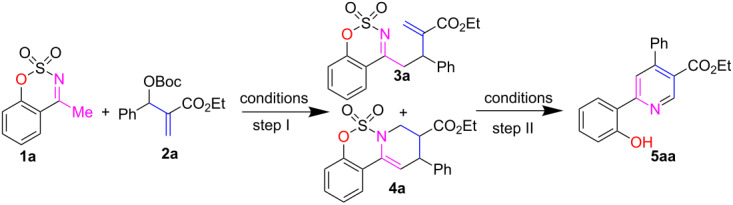				

entry	conditions of step I and step II	yield (%)^c^		
		
		**3a**	**4a**	**5aa**

1	[I]^a^ DABCO (20 mol %), toluene, rt, 14 h	87	4	0
2	[I]^a^ DABCO (20 mol %), toluene, 60 °C, 6 h	0	88	>2
3	[I]^d^ DABCO (1.0 equiv), toluene, 70 °C, 6 h	0	85	>4
4	[I]^a^ DABCO (20 mol %), toluene, 60 °C, 6 h [II]^b^ DBU (1.2 equiv), toluene, 60 °C, air, 5 h			76
5	[I]^a^ DABCO (20 mol %), THF, 60 °C, 6 h [II]^b^ DBU (1.2 equiv), THF, 60 °C, air, 5 h			68
6	[I]^a^ DABCO (20 mol %), MeCN, 60 °C, 6 h [II]^b^ DBU (1.2 equiv), MeCN, 60 °C, air, 5 h			50
7	[I]^a^ DABCO (20 mol %), DMF, 60 °C, 6 h [II]^b^ DBU (1.2 equiv), DMF, 60 °C, air, 5 h			30
8	[I]^a^ DABCO (20 mol %), DMSO, 60 °C, 6 h [II]^b^ DBU (1.2 equiv), DMSO, 60 °C, air, 5 h			19
9	[I]^a^ Et_3_N (20 mol %), toluene, 60 °C, 6 h [II]^b^ DBU (1.2 equiv), toluene, 60 °C, air, 5 h			<7
10	[I]^a^ DMAP (20 mol %), toluene, 60 °C, 6 h [II]^b^ DBU (1.2 equiv), toluene, 60 °C, air, 5 h			<5
11	[I]^e^ DBU (1.0 equiv), toluene, 60 °C, air, 5 h			ND^f^
12	[I]^a^ PPh_3_ (20 mol %), toluene, 60 °C, 6 h [II]^b^ DBU (1.2 equiv), toluene, 60 °C, air, 5 h			21

^a^Unless otherwise specified, all of the above reactions were conducted with *N*-sulfonyl ketimine **1a** (0.2 mmol), **2a** (0.26 mmol) and base (0.04 mmol, 20 mol %) in the specified dry solvent (1.0 mL) and temperature under atmospheric conditions. ^b^After completion of step I, DBU (1.2 equiv) was added directly to the reaction mixture. ^c^Yield of isolated product after column chromatography. ^d^DABCO (1.0 equiv) was used at 70 °C. ^e^Reaction was carried out using 1.0 equiv of DBU. ^f^ND = not detected.

Gratifyingly, high yields (88%) of cyclized product **4a** and a trace amount of desired nicotinate **5aa** (2% yield) were obtained when the reaction was conducted at 60 °C (entry 2, Table 1). By increasing the temperature as well as the loading of DABCO (1.0 equiv, entry 3, Table 1), a similar result was observed. At this situation, we surmised that step II may require a stronger base like DBU which will facilitate the aromatization process. For this purpose, DBU (1.2 equiv, entry 4, Table 1) was added to the reaction mixture at 60 °C after completion of step I. Pleasantly, 76% yield of wanted **5aa** was isolated after 5 h. Attempts to optimize the reaction conditions using several solvents (THF, MeCN, DMF and DMSO) all led to lower yields (19–68%, entries 5–8, Table 1) as compared to the toluene-mediated reaction (76%, entry 4, Table 1). Next, several N/P-containing Lewis bases such as Et_3_N, DMAP, DBU and PPh_3_ were tested for this reaction in toluene and resulted in very poor yields (5–21%, entries 9–12).

**Scheme 2:**
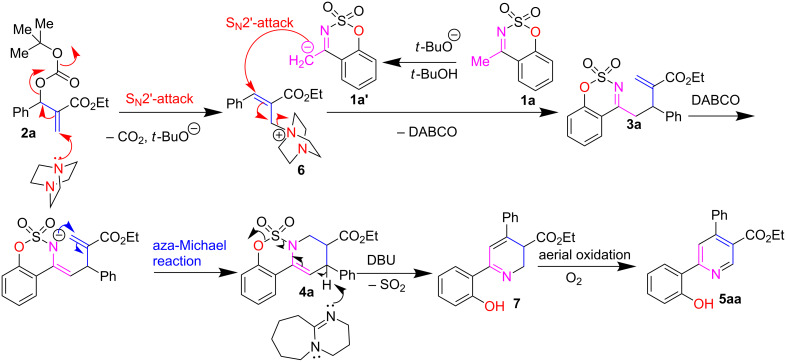
A possible mechanism for this sequential reaction.

Based on the above experimental results as well as our previous report on DABCO-catalyzed reactions of cyclic sulfamidate imines with MBH carbonates of isatins [75], a plausible mechanism is presented and depicted in Scheme 2. For the first step, the nucleophilic Lewis base DABCO reacts with **2a** in an S_N_2' fashion to make a very reactive allyl ammonium intermediate **6**. The latter further involves in the S_N_2' reaction with in situ generated carbanion intermediate **1a'** forming S_N_2-adduct **3a**. It undergoes an intramolecular aza-Michael reaction in the presence of DABCO, leading to tricyclic product **4a**. For the second step, DBU (strong base) abstracts an allylic proton of **4a**, followed by elimination of SO_2_ to give intermediate **7**. Finally, the desired product **5aa** is formed from **7** via aerial oxidation under open-flask conditions.

**Scheme 3:**
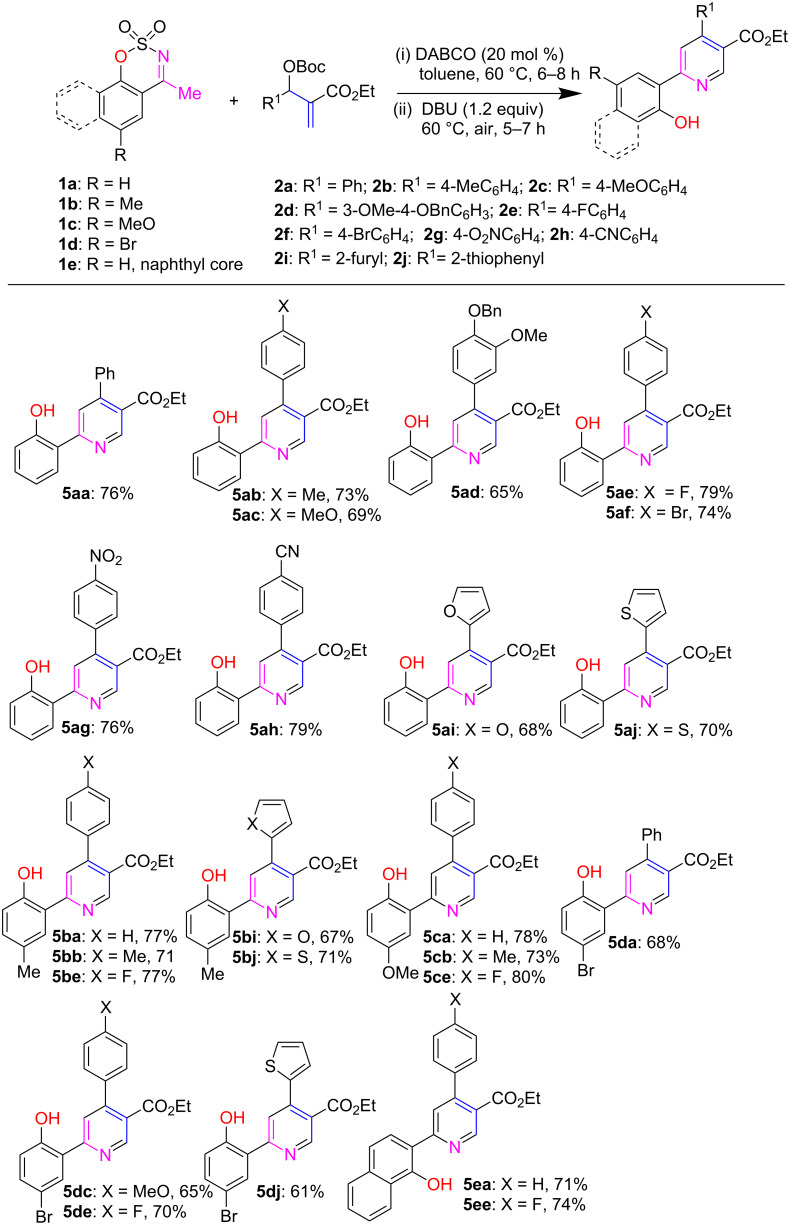
Substrate scope for (2-hydroxyaryl)nicotinates syntheses. The reaction was performed with **1a**–**e** (0.2 mmol), **2a**–**j** (0.26 mmol) and DABCO (0.04 mmol, 20 mol %) in toluene at 60 °C for 6–8 h, followed by the addition of DBU (0.24 mmol, 1.2 equiv) at the same temperature in an open atmosphere.

With the optimization reaction conditions in hand, we decided to explore the generality and scope of the reaction by reacting several aryl/heteroaryl-substituted MBH carbonates derived from acrylates **2a**–**j** and 4-methyl-*N*-sulfonyl ketimines **1a**–**e** under present sequential reaction conditions. The results are incorporated in Scheme 3. The regioselective allylic alkylation/aromatization reaction between 4-methyl-*N*-sulfonyl ketimine **1a** and several aryl-substituted MBH carbonates having electron donating (Me, MeO and BnO) and withdrawing substituents (F, Br, CN and NO_2_) on the benzene rings proceeded smoothly under the present conditions. The results showed that electron donating substituents of MBH carbonates produced 65–73% yields of the corresponding (2-hydroxyphenyl)nicotinates **5ab**–**5ad** which are slightly lower yields as compared to electron withdrawing ones (74–79% yields, **5ae**–**5ah**) under identical conditions. Furthermore, heteroaryl-substituted MBH carbonates **2i** and **2j** also afforded 68% and 70% yields of **5ai** and **5aj**, respectively.

Interestingly, the coupling reaction proceeded nicely not only with *N*-sulfonyl ketamine **1d** bearing an electron poor Br atom but also substrates **1b** and **1c** having electron donating substituents (Me, MeO) on the aryl rings, although the latter produced better yields than the former one. By this C–C/C–N bonds forming procedure, N-sulfonyl ketimine derived from a bulky α-naphthol moiety was found to be a suitable coupling reagent, leading to the corresponding α-naphthol-substituted nicotinate derivatives **5ea** and **5ee** in 71% and 74% yields, respectively.

**Scheme 4:**
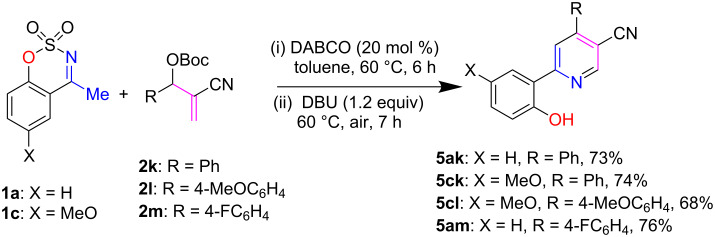
One-pot synthesis of (2-hydroxyaryl)nicotinonitriles **5ak**–**5am**.

Next, we further expanded our present methodology for the synthesis of 2-hydroxyarylated nicotinonitriles by using MBH carbonates derived from an acrylonitrile. Experimental data revealed that several aryl-substituted MBH carbonates **2k**–**2m** underwent [3 + 3] cyclization with *N*-sulfonyl ketimines **1a** and **1c** in our established reaction conditions, resulting in good yields (68–76%) of aforesaid heterocycles **5ak**–**5am** (Scheme 4).

## Conclusion

In the current manuscript, a unique one-pot two-step sequential reaction of 4-methyl *N*-sulfonyl ketimines with MBH carbonates of acrylate/acrylonitrile catalyzed by DABCO, followed by aromatization using DBU as a base in an open-flask has been developed. This smart oxidant metal-free C–C/C–N bond forming process leads to an array of functionalized nicotinates/nicotinonitriles possessing an interesting phenolic moiety at C6 position in good to high yields. Moreover, the current process is mild enough to be applied on a broad range of functional groups. Further studies on the application of this reaction with broader substrate scope as well as the biological evaluation of the synthesized pyridines are in progress which will be documented in due course of time.

## Supporting Information

File 1 Synthetic protocols, characterization data and copies of ^1^H and ^13^C NMR spectra.
