# Supplementary material for: DABCO- and DBU-promoted one-pot reaction of N-sulfonyl ketimines with Morita–Baylis–Hillman carbonates: a sequential approach to (2-hydroxyaryl)nicotinate derivatives
Source: Beilstein J Org Chem. 2018 Nov 2;14:2771–8. doi: 10.3762/bjoc.14.254 (PMC6244423; doi:10.3762/bjoc.14.254)
Supplement: File 1 — Synthetic protocols, characterization data and copies of 1H and 13C NMR spectra. [file Beilstein_J_Org_Chem-14-2771-s001.pdf]

Supporting Information

for

DABCO- and DBU-promoted one-pot reaction of *N*-sulfonyl ketimines with Morita–Baylis–Hillman carbonates: a sequential approach to (2-hydroxyaryl)nicotinate derivatives

Soumitra Guin [‡,1], Raman Gupta[‡,1], Debashis Majee[1] and Sampak Samanta*[1]

Address: [1]Discipline of Chemistry, Indian Institute of Technology Indore, Simrol, Indore, 453552, Madhya Pradesh, India

Email: Sampak Samanta - sampaks@iiti.ac.in

*Corresponding author

‡Both the authors equally contributed to this work

Synthetic protocols, characterization data and copies of ^{1}H and ^{13}C NMR spectra

General information……………………………………………………S2
Experimental procedures………………………………………………S2
Characterization data……………………………………………..……S3–S14
References……………………………………………………………..S14
Copies of ^{1}H and ^{13}C NMR of **5aa–5am**……………………………..S15–S44

General information

All the reactions were carried out under air and monitored by TLC using Merck 60 F_{254} pre-coated silica-gel plates (0.25 mm thickness) and the products were visualized by UV detection. Flash chromatography was carried out with silica-gel (200–300 mesh). ^{1}H and ^{13}C NMR spectra were recorded on a BrukerAvance (III) 400 Hz spectrometer. Data for ^{1}H NMR are reported as a chemical shift (δ ppm), multiplicity (s = singlet, d = doublet, q = quartet, m = multiplet), coupling constant *J* (Hz), integration, and assignment, data for ^{13}C are reported as a chemical shift. High resolutions mass spectral analyses (HRMS) were carried out using ESI-QTOF-MS (Microtof Q-II Bruker, condition: capillary voltage = −4500V, temperature = 250 °C, N_2 gas flow = 7 lit/min, nebulizer pressure = 2 bar, instrument is calibrated using ESI tune low calibration mixture before HRMS sample analysis). Melting points were recorded on an Electro thermal melting point apparatus and are uncorrected. FT-IR spectra were recorded on a KBr plate.

Experimental procedures

One-pot synthesis of compound 4a: A mixture of sulfonyl ketimine (**1a**, 0.2 mmol), MBH carbonates (**2a**, 0.26 mmol), and DABCO (0.04 mmol) in anhydrous toluene (1.0 mL) was heated at 60 °C for 6h. Then the crude product was directly purified by column chromatography technique to give the product **4a** as a mixture of diastereomer in 88% yield.

General procedure for the synthesis of compounds 5aa-5am: To a stirred solution of *N*-sulfonyl ketimine[1] (**1**, 0.2 mmol), MBH carbonates[2] (**2**, 0.26 mmol), and DABCO (0.04 mmol) in anhydrous toluene (1.0 mL) was heated at 60 °C. After completion of step I (monitored by TLC), DBU (1.2 equiv) was added to the above reaction mixture at same temperature. After 6–7 h, the reaction mixture was extracted with ethyl acetate (3 × 10 mL), washed with brined and dried over anhydrous Na_2SO_4. Afterwards, evaporation of organic solvent gave the crude product which was purified through column chromatography over silica-gel to afford the pure product.

All the obtained products (**5aa–5am**) were characterized by IR, ^{1}H NMR, ^{13}C NMR and HRMS data.

Characterization data

Ethyl 10-phenyl-9,10-dihydro-8*H*-benzo[*e*]pyrido[1,2-*c*][1,2,3]oxathiazine-9-carboxylate

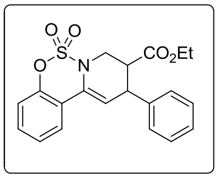

6,6-dioxide (4a): gummy liquid; yield 88%; R_f = 0.67 (EtOAc:hexane = 1:6); IR (KBr) ν 1731, 1596, 1537, 1488, 1474, 1391, 1366 cm^{-1} ; ^{1}H NMR (400 MHz, $CDCl_3$) *δ* (major isomer) 7.61 (d, *J*= 8.0 Hz, 1H), 7.31-7.40 (m, 3H), 7.20-7.30 (m, 4H), 7.13 (d, *J*= 8.2 Hz, 1H), 5.89 (d, *J*= 3.5 Hz, 1H), 4.11-4.16 (m, 3H), 4.03-4.07 (m, 1H), 3.93-3.98 (m, 1H), 2.92-2.97 (m, 1H), 1.19 (t, *J*= 7.0 Hz, 3H) ppm; ^{13}C NMR (100 MHz, $CDCl_3$) δ (major isomer) 171.3, 148.5, 141.9, 132.4, 130.6, 128.9, 128.2, 127.5, 126.1, 124.1, 119.2, 118.8, 108.3, 61.5, 45.6, 43.5, 41.4, 14.0 ppm; HRMS (ESI) m/z calcd for $C_{20}H_{19}NO_5S[M+H]^+$ 386.1052, found 386.1065.

Ethyl 6-(2-hydroxyphenyl)-4-phenylnicotinate(5aa): colorless solid; mp 121-123 ºC; yield

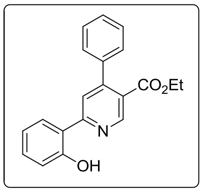

76% (48.5 mg); R_f = 0.7 (EtOAc:hexane = 1:6); IR (KBr) ν 3433, 1698, 1593, 1530, 1499, 1376 cm^{-1};^{1}H NMR (400 MHz, $CDCl_3$) *δ* 13.99 (s, 1H), 9.00 (s, 1H), 7.86 (s, 1H), 7.82 (d, *J* = 8.0 Hz, 1H), 7.46-7.46 (m, 3H), 7.35-7.37 (m, 3H), 7.04-7.07 (m, 1H), 6.89-6.93 (m, 1H), 4.18 (q, *J* = 7.0 Hz, 2H), 1.09 (t, *J* = 7.0 Hz, 3H) ppm; ^{13}C NMR (100 MHz, $CDCl_3$) *δ* 165.9, 160.6, 159.8, 152.1, 148.1, 138.9, 132.6, 128.7, 128.4, 128.0, 126.7, 124.2, 120.4, 119.1, 118.9, 118.1, 61.4, 13.7 ppm; HRMS (ESI) m/z calcd for $C_{20}H_{17}NO_3[M+Na]^+$ 342.1101, found 342.1120.

Ethyl 6-(2-hydroxyphenyl)-4-(4-methylphenyl)nicotinate (5ab): light yellow solid; mp 124-

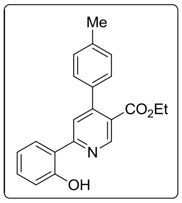

126 ºC; yield 73% (48.6 mg); R_f = 0.7 (EtOAc:hexane = 1:6); IR (KBr) ν 3442, 1699, 1596, 1529, 1511, 1306 cm^{-1}; ^1H NMR (400 MHz, $CDCl_3$) δ 14.0 (s, 1H), 8.95 (s, 1H), 7.83 (s, 1H), 7.80 (d, *J* = 8.0 Hz, 1H), 7.31-7.35 (m, 1H), 7.22-7.26 (m, 4H), 7.03 (d, *J* = 8.0 Hz, 1H), 6.87-6.91 (m, 1H), 4.20 (q, *J* = 7.2 Hz, 2H), 2.42 (s, 3H), 1.13 (t, *J* = 7.0 Hz, 3H) ppm; ^{13}C NMR (100 MHz, $CDCl_3$) δ 166.0, 160.6, 159.7, 152.1, 148.0, 138.7, 135.9, 132.5, 129.1, 128.0, 126.7, 124.2, 120.4, 119.1, 118.9, 118.1, 61.4, 21.3, 13.7 ppm; HRMS (ESI) m/z calcd for $C_{21}H_{19}NO_3[M+Na]^+$ 356.1257, found 356.1270

Ethyl 6-(2-hydroxyphenyl)-4-(4-methoxyphenyl)nicotinate (5ac): yellow solid; mp 131-133

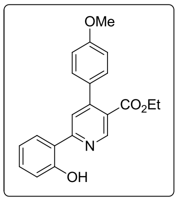

ºC yield 69% (48.1 mg); R_f = 0.68 (EtOAc:hexane = 1:6); IR (KBr) ν 3433, 1701, 1597, 1531, 1511, 1462, 1305 cm^{-1}; ^1H NMR (400 MHz, $CDCl_3$) δ 14.02 (s, 1H), 8.95 (s, 1H), 7.84 (s, 1H), 7.82-7.82 (m, 1H), 7.31-7.36 (m, 3H), 6.98-7.06 (m, 3H), 6.89-6.93 (m, 1H), 4.22 (q, *J* = 7.0 Hz, 2H), 3.87 (s, 3H), 1.16 (t, *J* = 7.0 Hz, 3H) ppm; ^{13}C NMR (100 MHz, $CDCl_3$) δ 166.2, 160.6, 160.2, 159.7, 151.6, 148.0, 132.5, 131.0, 129.5, 126.7, 124.2, 120.3, 119.0, 118.9, 118.2, 113.9, 61.5, 55.4, 13.9 ppm; HRMS (ESI) m/z calcd for $C_{21}H_{19}NO_4[M+Na]^+$ 372.1206, found 372.1220.

Ethyl 4-(4-(benzyloxy)-3-methoxyphenyl)-6-(2-hydroxyphenyl)nicotinate (5ad): colorless

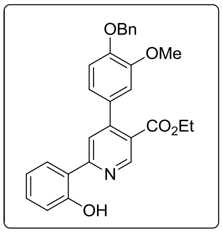

solid; mp 140-142 ºC; yield 65% (59.2 mg); R_f = 0.60 (EtOAc:hexane = 1:6); IR (KBr) ν 3443, 1705, 1626, 1581, 1474, 1369 cm^{-1}; ^1H NMR (400 MHz, $CDCl_3$) δ 13.80 (s, 1H), 8.92 (s, 1H), 7.85 (s, 1H), 7.82 (d, *J*= 8.0 Hz, 1H), 7.46 (d, *J*= 7.3 Hz, 2H), 7.32-7.40 (m, 4H), 7.04 (d, *J*= 8.3 Hz, 1H), 6.96 (d, *J*= 8.0 Hz, 1H), 6.86-6.93 (m, 3H), 5.22 (s, 2H), 4.19 (q, *J*= 7.0 Hz, 2H), 3.92 (s, 3H), 1.11 (t, *J*= 7.0 Hz, 3H) ppm; ^{13}C NMR (100 MHz, $CDCl_3$) δ 166.3, 160.6, 159.7, 151.5, 149.5, 148.8,

147.8, 136.8, 132.5, 131.8, 128.6, 128.0, 127.3, 126.7, 124.5, 120.7, 120.2, 119.1, 118.9, 118.2, 113.6, 111.9, 71.0, 61.5, 56.2, 13.9 ppm. HRMS (ESI) m/z calcd for $C_{28}H_{25}NO_5[M+H]^+$ 456.1805, found 456.1807.

Ethyl 4-(4-fluorophenyl)-6-(2-hydroxyphenyl)nicotinate (5ae): white solid; yield 79% (53.3 mg); mp 112-114 ºC; R_f = 0.69 (EtOAc:hexane = 1:6); IR (KBr) ν 3442, 1732, 1593, 1511, 1420, 1288 cm^{-1}; ^1H NMR (400 MHz, $CDCl_3$) δ 13.9 (s, 1H), 9.0 (s, 1H), 7.82 (s, 1H), 7.81 (s, 1H), 7.34-7.35 (m, 3H), 7.14-7.19 (m, 2H), 7.05 (d, J = 8.0 Hz, 1H), 6.91-6.93 (m, 1H), 4.21 (q, J = 7.0 Hz, 2H), 1.15 (t, J = 7.0 Hz, 3H) ppm; ^{13}C NMR (100 MHz, $CDCl_3$) δ 166.0, 163.1 (J_{C-F} = 247.2 Hz), 160.5, 160.0, 151.1, 148.3, 134.9, 132.7, 129.9 (J_{C-F} = 8.0 Hz), 126.7, 123.9, 120.4, 119.1, 119.0, 118.0, 115.4 (J_{C-F} = 21.8 Hz), 61.5, 13.9 ppm ; HRMS (ESI) m/z calcd for $C_{20}H_{16}FNO_3[M+Na]^+$ 360.1006, found 360.1020.

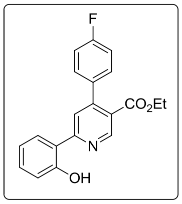

Ethyl 4-(4-bromophenyl)-6-(2-hydroxyphenyl)nicotinate (5af): colorless solid; mp 144-146 ºC; yield 74% (58.9 mg) ; R_f = 0.7 (EtOAc:hexane = 1:6); IR (KBr) ν 3441, 1731, 1593, 1534, 1420, 1364 cm^{-1}; ^1H NMR (400 MHz, $CDCl_3$) δ 13.86 (s, 1H), 9.09 (s, 1H), 7.79-7.82 (m, 2H), 7.73 (d, J = 8.0 Hz, 2H), 7.48 (d, J= 7.8 Hz, 2H), 7.35-7.38 (m, 1H), 7.06 (d, J = 8.0 Hz, 1H), 6.89-6.93 (m, 1H), 4.19 (q, J = 7.0 Hz, 2H), 1.11 (t, J = 7.0 Hz, 3H) ppm; ^{13}C NMR (100 MHz, $CDCl_3$) δ 166.6, 160.6, 159.9, 149.5, 147.7, 144.6, 138.3, 132.6, 126.8, 122.2, 119.2, 119.0, 118.3, 116.4, 113.2, 112.5, 62.0, 14.3 ppm; HRMS (ESI): m/z calcd for $C_{20}H_{16}{}^{79}BrNO_3[M+Na]^+$ 420.0206, found 420.0219; m/z calcd for $C_{20}H_{16}{}^{81}BrNO_3[M+Na]^+$ 422.0185, found 422.0203.

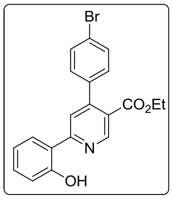

Ethyl 6-(2-hydroxyphenyl)-4-(4-nitrophenyl)nicotinate (5ag): yellow solid; mp 133-135 ºC;

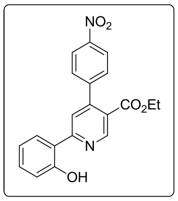

yield 76% (55.3 mg); R_f = 0.6 (EtOAc:hexane = 1:6); IR (KBr) ν 3427, 1720, 1591, 1516, 1420, 1348 cm^{-1}; ^{1}H NMR (400 MHz, $CDCl_3$) δ 13.90 (s, 1H), 9.03 (s, 1H), 7.81 (s, 1H), 7.79 (m, 1H), 7.60 (d, *J*= 8.3 Hz, 2H), 7.34-7.38 (m, 1H), 7.24 (m, 2H), 7.05 (d, *J* = 8.3 Hz, 1H), 6.90-6.93 (m, 1H), 4.21 (q, *J* = 7.0 Hz, 2H), 1.16 (t, *J* = 7.0 Hz, 3H) ppm; ^{13}C NMR (100 MHz, $CDCl_3$) δ 166.5, 160.4, 159.8, 149.3, 147.6, 144.4, 138.2, 132.5, 126.7, 122.0, 119.1, 118.9, 118.2, 116.3, 113.1, 112.4, 61.9, 14.2 ppm; HRMS (ESI) m/z calcd for $C_{20}H_{16}N_2O_5$[M+Na]$^+$ 387.0951, found 387.0962.

Ethyl 4-(4-cyanophenyl)-6-(2-hydroxyphenyl)nicotinate (5ah): yellow solid; mp 152-154 ºC

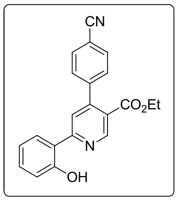

yield 79% (54.3 mg) R_f = 0.55 (EtOAc:hexane = 1:6); IR (KBr) ν 3404, 2224, 1713, 1599, 1534, 1474, 1302 cm^{-1}; ^{1}H NMR (400 MHz, $CDCl_3$) δ 13.80 (s, 1H), 9.11 (s, 1H), 7.78-7.80 (m, 3H), 7.75 (s, 1H), 7.47 (d, *J* = 8.0 Hz, 2H), 7.35-7.39 (m, 1H), 7.06 (d, *J*= 8.3 Hz, 1H), 6.90-6.94 (m, 1H), 4.21 (q, *J* = 7.0 Hz, 2H), 1.16 (t, *J* = 7.0 Hz, 3H) ppm; ^{13}C NMR (100 MHz, $CDCl_3$) δ 164.9, 160.7, 160.4,150.5, 148.8, 143.7, 133.1, 132.0, 128.9, 126.8, 123.0, 120.1, 119.3, 119.1, 118.4, 117.7, 112.5, 61.7, 13.9 ppm; HRMS (ESI) m/z calcd for $C_{21}H_{16}N_2O_3$[M+H]$^+$ 345.1234, found 345.1250.

Ethyl 4-(furan-2-yl)-6-(2-hydroxyphenyl)nicotinate (5ai): brown solid; mp 119-121 ºC; yield

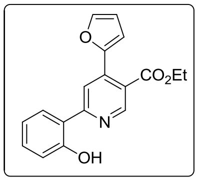

68% (42.01 mg); R_f = 0.7 (EtOAc:hexane = 1:6); IR (KBr) ν 3431, 1723, 1600, 1491, 1362 cm^{-1}; ^{1}H NMR (400 MHz, $CDCl_3$) δ 13.9 (s, 1H), 8.8 (s, 1H), 8.18 (s, 1H), 7.88 (d, *J* = 8 Hz, 1H), 7.61 (s, 1H), 7.33-7.37 (m, 1H), 7.02-7.05 (m, 2H), 6.93-6.96 (m, 1H), 6.58 (s, 1H), 4.40 (q, *J* = 7.0 Hz, 2H), 1.36 (t, *J* = 7.4 Hz, 3H) ppm; ^{13}C NMR (100 MHz, $CDCl_3$) δ 166.5, 160.4, 159.8, 149.3, 147.6, 144.4, 138.2,

132.5, 126.7, 122.0, 119.1, 118.9, 118.2, 116.3, 113.1, 112.4, 61.8, 14.1 ppm; HRMS (ESI) m/z calcd for $C_{18}H_{15}NO_4[M+Na]^+$ 332.0893, found 332.0907.

Ethyl 6-(2-hydroxyphenyl)-4-(thiophen-2-yl)nicotinate (5aj): brown solid; mp 125-127 ºC

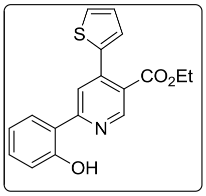

yield 70% (45.6 mg); R_f = 0.7 (EtOAc:hexane = 1:6); IR (KBr) ν 3430, 1724, 1603, 1531, 1492, 1360 cm^{-1};^{1}H NMR (400 MHz, $CDCl_3$): δ 13.8 (s, 1H), 8.89 (s, 1H), 7.95 (s, 1H), 7.82 (d, *J* =7.8 Hz, 1H), 7.50 (d, *J* = 6.0 Hz, 1H), 7.34-7.37 (m, 1H), 7.23-7.27 (m, 1H), 7.13-7.15 (m, 1H), 7.05 (d, *J* = 8.2 Hz, 1H), 6.91-6.95 (m, 1H), 4.29 (q, *J* = 7.3 Hz, 2H), 1.24 (t, *J*= 7.0 Hz, 3H) ppm; ^{13}C NMR (100 MHz, $CDCl_3$) δ 166.0, 160.5, 159.8, 147.8, 143.9, 138.9, 132.6, 128.3, 127.9, 127.7, 126.7, 124.4, 120.4, 119.1, 118.9, 118.0, 61.7, 13.9 ppm; HRMS (ESI) m/z calcd for $C_{18}H_{15}NO_3S[M+Na]^+$ 348.0665, found 348.0680

Ethyl 6-(2-hydroxy-5-methylphenyl)-4-phenylnicotinate (5ba): colorless solid; mp 129-131

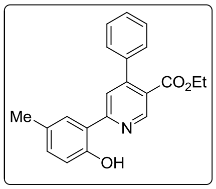

ºC; yield 77% (51.3 mg); R_f = 0.67 (EtOAc:hexane = 1:6); IR (KBr) ν 3457, 1720, 1594, 1532, 1497, 1396 cm^{-1}; ^{1}H NMR (400 MHz, $CDCl_3$) δ 13.76 (s, 1H), 8.99 (s, 1H), 7.84 (s, 1H), 7.60 (s, 1H), 7.46-7.47 (m, 3H), 7.37-7.39 (m, 2H), 7.16 (d, *J* = 8.0 Hz, 1H), 6.94-6.97 (m, 1H), 4.18 (q, *J* = 7.0 Hz, 2H), 2.31 (s, 3H), 1.09 (t, *J* = 7.0 Hz, 3H) ppm; ^{13}C NMR (100 MHz, $CDCl_3$) δ 165.9, 159.9, 158.4, 152.0, 148.2, 139.0, 133.5, 128.6, 128.3, 128.1, 128.0, 126.8, 124.0, 120.3, 118.7, 117.7, 61.4, 20.7, 13.7 ppm; HRMS (ESI) m/z calcd for $C_{21}H_{19}NO_3[M+Na]^+$ 356.1257, found 356.1268

Ethyl 6-(2-hydroxy-5-methylphenyl)-4-(4-methylphenyl)nicotinate (5bb): colorless solid; mp

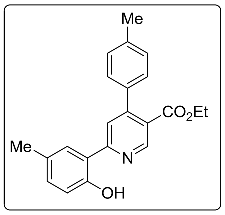

132-134 ºC; yield 71% (49.3 mg); R_f = 0.67 (EtOAc:hexane = 1:6); IR (KBr) ν 3438, 1729, 1608, 1483, 1427, 1387 cm^{-1}; ^{1}H NMR (400 MHz, $CDCl_3$) δ 13.75 (s, 1H), 8.99 (s, 1H), 7.84 (s, 1H), 7.60 (s, 1H), 7.26-7.28 (m, 3H), 7.15-7.17 (m, 2H), 6.94-6.96 (m, 1H), 4.20 (q, J = 7.0 Hz, 2H), 2.82 (s, 3H), 2.31 (s, 3H), 1.14 (t, J = 7.0 Hz, 3H) ppm; ^{13}C NMR (100 MHz, $CDCl_3$) δ 165.7, 158.2, 150.1, 148.8, 139.5, 139.4, 135.9, 134.8, 129.9, 129.5, 127.1, 126.9, 120.9, 120.8, 61.8, 21.3, 21.2, 14.3 ppm; HRMS (ESI) m/z calcd for $C_{22}H_{21}NO_3[M+H]^+$ 348.1594, found 348.1601.

Ethyl 4-(4-fluorophenyl)-6-(2-hydroxy-5-methylphenyl)nicotinate (5be): white solid; mp

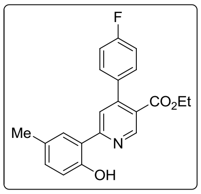

110-112 ºC; yield 77% (54.1 mg); R_f = 0.66 (EtOAc:hexane = 1:6); IR (KBr) ν 3439, 1730, 1597, 1491, 1366 cm^{-1};^{1}H NMR (400 MHz, $CDCl_3$) δ 13.70 (s, 1H), 9.0 (s, 1H), 7.80 (s, 1H), 7.59 (s, 1H), 7.34-7.37 (m, 2H), 7.14-7.19 (m, 3H), 6.94-697 (m, 1H), 4.20 (q, J = 7.0 Hz, 2H), 2.32 (s, 3H), 1.15 (t, J = 7.0 Hz, 3H) ppm; ^{13}C NMR (100 MHz, $CDCl_3$) δ 165.4, 162.7 ($J_{C\text{-}F}$ = 247.1 Hz), 159.8, 158.1, 150.8, 148.1, 134.6, 133.3, 129.6 ($J_{C\text{-}F}$ = 8.0 Hz), 127.8, 126.4, 123.4, 120.1, 118.6, 117.3, 115.1 ($J_{C\text{-}F}$= 22.0 Hz), 61.2, 20.4, 13.6 ppm; HRMS (ESI) m/z calcd for $C_{21}H_{18}FNO_3[M+Na]^+$ 374.1163, found 374.1174.

Ethyl 4-(furan-2-yl)-6-(2-hydroxy-5-methylphenyl)nicotinate (5bi): brown solid; mp 100-102

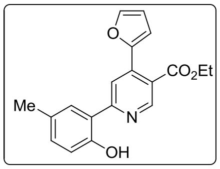

ºC; yield 67% (43.3 mg); R_f = 0.68 (EtOAc:hexane = 1:6); IR (KBr) ν 3448, 1719, 1547, 1523, 1392 cm^{-1}; ^{1}H NMR (400 MHz, $CDCl_3$) δ 13.65 (s, 1H), 8.79 (s, 1H), 8.14 (s, 1H), 7.61-7.64 (m, 2H), 7.15-7.17 (m, 1H), 7.03 (d, J = 3.2 Hz, 1H), 6.94 (d, J = 8.3 Hz, 1H), 6.57-6.58 (m, 1H), 4.40 (q, J = 8.3 Hz, 2H), 2.35 (s, 3H), 1.36 (t, J = 7.3 Hz, 3H) ppm; ^{13}C NMR (100 MHz, $CDCl_3$) δ 166.5, 159.9, 158.2, 149.4, 147.6,

144.4, 138.2, 133.4, 128.1, 126.7, 121.9, 118.6, 117.8, 116.2, 113.0, 112.4, 61.8, 20.7, 14.2 ppm; HRMS (ESI) m/z calcd for $C_{19}H_{17}NO_4[M+Na]^+$ 346.1050, found 346.1062.

Ethyl 6-(2-hydroxy-5-methylphenyl)-4-(thiophen-2-yl)nicotinate (5bj): brown solid; mp 105-

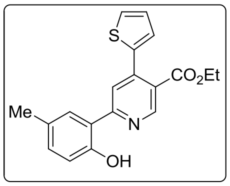

107 ºC; yield 72% (48.9 mg); R_f = 0.68 (EtOAc:hexane = 1:6); IR (KBr) ν 3422, 1719, 1596, 1523, 1487, 1364 cm^{-1}; ^1H NMR (400 MHz, $CDCl_3$): δ 13.8 (s, 1H), 8.89 (s, 1H), 7.93 (s, 1H), 7.59 (s, 1H), 7.49-7.51 (m, 1H), 7.15-7.18 (m, 2H), 6.95 (d, J = 8.0 Hz, 1H), 4.29 (q, J = 7.3 Hz, 2H), 1.24 (t, J = 7.0 Hz, 3H) ppm; ^{13}C NMR (100 MHz, $CDCl_3$) δ 166.1, 159.9, 158.3, 147.9, 143.9, 139.1, 133.6, 128.3, 128.1, 127.8, 127.7, 126.7, 124.3, 120.4, 118.7, 117.6, 61.7, 20.7, 13.9 ppm; HRMS (ESI) m/z calcd for $C_{19}H_{17}NO_3S[M+Na]^+$ 362.0821, found 362.0833.

Ethyl 6-(2-hydroxy-5-methoxyphenyl)-4-phenylnicotinate (5ca): colorless solid; mp 118-120

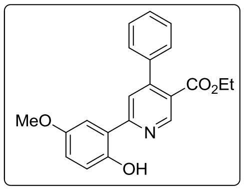

ºC; yield 78% (54.5 mg); R_f = 0.68 (EtOAc:hexane = 1:6); IR (KBr) ν 3449, 1719, 1593, 1529, 1491, 1399 cm^{-1}; ^1H NMR (400 MHz, $CDCl_3$) δ 13.49 (s, 1H), 9.00 (s, 1H), 7.79 (s, 1H), 7.46-7.47 (m, 3H), 7.36-7.38 (m, 2H), 7.27-7.29 (m, 1H), 6.99-6.99 (m, 1H), 6.96-7.0 (m, 2H), 4.16 (q, J = 7.0 Hz, 2H), 3.80 (s, 3H), 1.08 (t, J = 7.0 Hz, 3H) ppm; ^{13}C NMR (100 MHz, $CDCl_3$) δ 165.9, 159.6 154.8, 152.4, 152.1, 148.2, 138.9, 128.7, 128.4, 128.0, 124.2, 120.5, 119.6, 119.4, 118.0, 111.0, 61.4, 56.1, 13.8 ppm; HRMS (ESI) m/z calcd for $C_{21}H_{19}NO_4[M+Na]^+$ 372.1206, found 372.1220

Ethyl 6-(2-hydroxy-5-methoxyphenyl)-4-(4-methylphenyl)nicotinate (5cb): light yellow

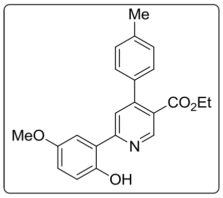

solid; mp 125-127ºC; yield 73% (52.3 mg); R_f = 0.65 (EtOAc:hexane = 1:6); IR (KBr) ν 3438, 1721, 1595, 1514, 1462, 1387 cm^{-1}; ^1H NMR (400 MHz, $CDCl_3$) δ 13.48 (s, 1H), 8.95 (s, 1H), 7.76 (s, 1H), 7.23-7.26 (m, 5H), 6.95-6.96 (m, 2H), 4.17 (q, J = 7.0 Hz, 2H), 3.78 (s, 3H), 2.41 (s, 3H), 1.12 (t, J = 7.0 Hz, 3H) ppm;

^{13}C NMR (100 MHz, $CDCl_3$) δ 166.0, 159.5, 154.7, 152.3, 152.2, 148.1, 138.8, 135.9, 129.0, 128.0, 124.2, 120.5, 119.5, 119.3, 118.0, 111.0, 61.4, 56.1, 21.3, 13.8 ppm; HRMS (ESI) m/z calcd for $C_{22}H_{22}NO_4[M+Na]^+$ 386.1363, found 386.1275.

Ethyl4-(4-fluorophenyl)-6-(2-hydroxy-5-methoxyphenyl)nicotinate (5ce): colorless solid; mp

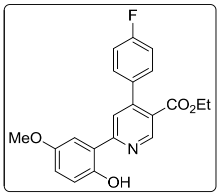

114-116 ºC; yield 80% (58.7 mg); R_f = 0.65 (EtOAc:hexane = 1:6); IR (KBr) ν 3448, 1714, 1594, 1512, 1463, 1395 cm^{-1}; ^{1}H NMR (400 MHz, $CDCl_3$) δ 13.41 (s, 1H), 9.01 (s, 1H), 7.75 (s, 1H), 7.33-7.36 (m, 2H), 7.26-7.28 (m, 1H), 7.14-7.19 (m, 2H), 6.97-6.99 (m, 2H), 4.20 (q, J = 7.0 Hz, 2H), 3.81 (s, 3H), 1.15 (t, J = 7.0 Hz, 3H) ppm; ^{13}C NMR (100 MHz, $CDCl_3$) δ 165.7, 163.1 (J_{C-F} = 247.9 Hz), 159.7, 154.8, 152.4, 151.2, 148.4, 134.8, 129.9 (J_{C-F}= 8.0 Hz), 124.0, 120.5, 119.6, 119.4, 117.9, 115.4 (J_{C-F}= 22.0 Hz), 111.2, 61.5, 56.1, 13.8 ppm; HRMS (ESI) m/z calcd for $C_{21}H_{18}FNO_4[M+Na]^+$ 390.1112, found 390.1120

Ethyl 6-(5-bromo-2-hydroxyphenyl)-4-phenylnicotinate (5da) : colorless solid; mp 120-122

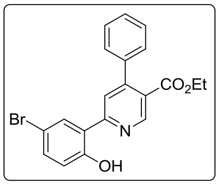

ºC; yield 68% (54.1 mg); R_f = 0.7 (EtOAc:hexane = 1:6); IR (KBr) ν 3448, 1731, 1594, 1536, 1472, 1390 cm^{-1};^{1}H NMR (400 MHz, $CDCl_3$) δ 14.02 (s, 1H), 8.99 (s, 1H), 7.91 (s, 1H), 7.80 (s, 1H), 7.47-7.47 (m, 3H), 7.37-7.42 (m, 2H), 6.94 (d, J = 8.2 Hz, 2H), 4.18 (q, J = 7.0 Hz, 2H), 1.08 (t, J = 7.0 Hz, 3H) ppm; ^{13}C NMR (100 MHz, $CDCl_3$) δ 165.8, 159.6, 158.4, 152.4, 148.1, 138.6, 135.1, 129.1, 128.8, 128.4, 128.0, 124.8, 120.8, 120.5, 119.7, 110.9, 61.6, 13.7 ppm; HRMS (ESI) m/z calcd for $C_{20}H_{16}{}^{79}BrNO_3[M+Na]^+$ 420.0206, found 420.0217; m/z calcd for $C_{20}H_{16}{}^{81}BrNO_3[M+Na]^+$ 422.0185, found 422.0197 .

Ethyl6-(5-bromo-2-hydroxyphenyl)-4-(4-methoxyphenyl)nicotinate (5dc): pale yellow solid;

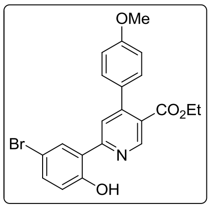

mp 136-138ºC; yield 65% (55.7 mg); R_f = 0.64 (EtOAc:hexane = 1:6); IR (KBr) ν 3443, 1706, 1653, 1581, 1515, 1474, 1338 cm^{-1}; ^{1}H NMR (400 MHz, $CDCl_3$) δ 14.00 (s, 1H), 8.94 (s, 1H), 7.91 (d, J = 2.0 Hz, 1H), 7.78 (s, 1H), 7.41 (dd, J_1= 2.0 Hz, J_2 = 7.6 Hz, 1H), 7.33 (d, J = 8.5 Hz, 2H), 7.0 (d, J = 8.5 Hz, 2H), 6.94 (d, J= 8.0 Hz, 1H), 4.22 (q, J = 7.04 Hz, 2H), 3.88 (s, 3H), 1.16 (t, J = 7.0 Hz, 3H) ppm; ^{13}C NMR (100 MHz, $CDCl_3$) δ 166.1, 160.4, 159.6, 158.2, 151.9, 148.0, 135.0, 129.5, 129.1, 124.9, 120.8, 120.4, 119.8, 114.0, 110.8, 61.6, 55.4, 13.9 ppm; HRMS (ESI) m/z calcd for $C_{21}H_{18}{}^{79}BrNO_4[M+Na]^+$ 450.0311, found 450.0323; m/z calcd for $C_{21}H_{18}{}^{81}BrNO_4[M+Na]^+$ 452.0291, found 452.0309.

Ethyl6-(5-bromo-2-hydroxyphenyl)-4-(4-fluorophenyl)nicotinate (5de): colorless solid; mp

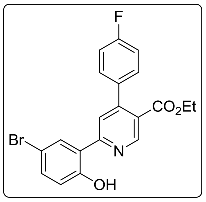

128-130 ºC; yield 70% (58.2 mg); R_f = 0.68 (EtOAc:hexane = 1:6); IR (KBr) ν 3423, 1730, 1595, 1537, 1488, 1390 cm^{-1}; ^{1}H NMR (400 MHz, $CDCl_3$) δ 13.96 (s, 1H), 9.0 (s, 1H), 7.90 (s, 1H), 7.76 (s, 1H), 7.40-7.43 (m, 1H), 7.34-7.37 (m, 2H), 7.15-7.20 (m, 2H), 6.94 (d, J = 8.8 Hz, 1H), 4.21 (q, J = 7.04 Hz, 2H), 1.15 (t, J = 7.0 Hz, 3H) ppm; ^{13}C NMR (100 MHz, $CDCl_3$) δ 165.5, 163.2 ($J_{C\text{-}F}$ = 247.8 Hz), 159.6, 158.4, 151.4, 148.3, 135.2, 134.5, 130.0, 129.9 ($J_{C\text{-}F}$ = 8.0 Hz), 124.6, 120.9, 120.5, 119.6, 115.5 ($J_{C\text{-}F}$ = 21.8 Hz), 110.9, 61.6, 13.8 ppm; HRMS (ESI) m/z calcd for $C_{20}H_{15}{}^{79}BrFNO_3[M+Na]^+$ 438.0112, found 438.0102; m/z calcd for $C_{20}H_{15}{}^{81}BrFNO_3[M+Na]^+$ 440.0091, found 440.0078.

6-(5-bromo-2-hydroxyphenyl)-4-(thiophen-2-yl)nicotinate (5dj): brown solid; mp 108-110

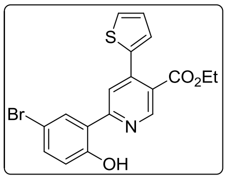

ºC; yield 61% (49.3 mg); R_f = 0.68 (EtOAc:hexane = 1:6); IR (KBr) ν 3457, 1720, 1594, 1532, 1497, 1396 cm^{-1}; ^{1}H NMR (400 MHz, $CDCl_3$) δ 13.88 (s, 1H), 8.88 (s, 1H), 7.89 (d, J = 7.8 Hz, 2H), 7.51-7.52 (m, 1H), 7.42 (d, J =

8.0 Hz, 1H), 7.25-7.26 (m, 1H), 7.13-7.16 (m, 1H), 6.93-6.95 (m, 1H), 4.29 (q, *J* = 7.0 Hz, 2H), 1.23 (t, *J* = 7.0 Hz, 3H) ppm; ^{13}C NMR (100 MHz, $CDCl_3$) δ 165.9, 159.5, 158.3, 147.8, 144.3, 138.6, 135.2, 129.1, 128.5, 128.2, 127.8, 125.1, 120.8, 120.5, 119.6, 110.9, 61.9, 13.9 ppm; HRMS (ESI) m/z calcd for $C_{18}H_{14}{}^{79}BrNO_3S[M+H]^+$ 403.9951, found 403.9956; m/z calcd for $C_{18}H_{14}{}^{81}BrNO_3S[M+H]^+$ 405.9930, found 405.9938.

Ethyl 6-(1-hydroxynaphthalen-2-yl)-4-phenylnicotinate (5ea): colorless solid; mp 139-141

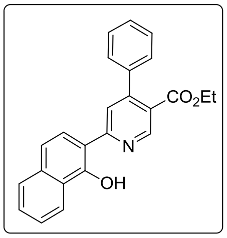

ºC; yield 71% (52.4 mg); R_f = 0.65 (EtOAc:hexane = 1:6); IR (KBr) ν 3442, 1694, 1596, 1519, 1483, 1379 cm^{-1}; ^{1}H NMR (400 MHz, $CDCl_3$) δ 15.49 (s, 1H), 9.04 (s, 1H), 8.50 (d, *J* = 7.6 Hz, 1H), 7.90 (s, 1H), 7.83 (d, *J* = 9.0 Hz, 1H), 7.76 (d, *J* = 7.8 Hz, 1H), 7.47-7.57 (m, 5H), 7.33-7.40 (m, 3H), 4.18 (d, *J* = 7.3 Hz, 2H), 1.10 (t, *J* = 7.0 Hz, 3H) ppm; ^{13}C NMR (100 MHz, $CDCl_3$) δ 165.9, 160.1, 158.9, 152.2, 147.9, 139.1, 135.7, 128.6, 128.3, 128.3, 128.0, 127.3, 128.4, 125.6, 123.9, 123.4, 122.7, 120.2, 118.6, 110.7, 61.4, 13.8 ppm; HRMS (ESI) m/z calcd for $C_{24}H_{19}NO_3[M+H]^+$ 370.1438, found 370.1443.

Ethyl 4-(4-fluorophenyl)-6-(1-hydroxynaphthalen-2-yl)nicotinate (5ee): colorless solid; mp

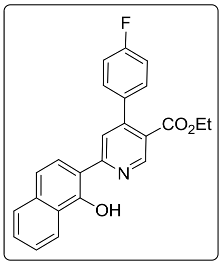

136-138 ºC; yield 74% (57.3 mg); R_f = 0.65 (EtOAc:hexane = 1:6); IR (KBr) ν 3448, 1695, 1596, 1465, 1377 cm^{-1};^{1}H NMR (400 MHz, $CDCl_3$) δ 15.45 (s, 1H), 9.05 (s, 1H), 8.50 (d, *J* = 7.8 Hz, 1H), 7.86 (s, 1H), 7.82 (d, *J* = 8.8 Hz, 1H), 7.76 (d, *J* = 7.6 Hz, 1H), 7.58-7.51 (m, 2H), 7.39-7.33 (m, 3H), 7.19-7.15 (m, 2H), 4.20 (d, *J* = 7.0 Hz, 2H), 1.16 (t, *J* = 7.0 Hz, 3H) ppm; ^{13}C NMR (100 MHz, $CDCl_3$) δ 165.7, 163.1 ($J_{C\text{-}F}$ = 247.0 Hz), 160.3, 158.9, 151.2, 148.2, 135.7, 135.1, 129.9 ($J_{C\text{-}F}$ = 8.0 Hz), 128.4, 127.3, 126.4, 125.6, 123.9, 123.2, 122.7, 120.3, 118.6, 115.4 ($J_{C\text{-}F}$ = 21.0

Hz), 110.6, 61.5, 13.9 ppm; HRMS (ESI) m/z calcd for $C_{24}H_{18}FNO_3[M+H]^+$ 388.1343, found 388.1349.

6-(2-Hydroxyphenyl)-4-phenylnicotinonitrile (5ak): light yellow solid; mp 134-136 ºC; yield 73% (39.7 mg) ; R_f = 0.6 (EtOAc:hexane = 1:6); IR (KBr) ν 3443, 2225, 1595, 1529, 1497, 1371 cm^{-1}; ^1H NMR (400 MHz, $CDCl_3$) δ 13.28 (s, 1H), 8.85 (s, 1H), 7.96 (s, 1H), 7.81 (d, *J* = 7.7 Hz, 1H), 7.64-7.67 (m, 2H), 7.37-7.41 (m, 1H), 7.27-7.29 (m, 3H), 7.05 (d, *J* = 8.0 Hz, 1H), 6.92-6.96 (m, 1H) ppm; ^{13}C NMR (100 MHz, $CDCl_3$) δ 160.8, 160.7, 153.4, 151.0, 135.6, 133.5, 130.5, 129.3, 128.4, 126.9, 119.5, 119.2, 117.7, 116.5, 106.0 ppm; HRMS (ESI) m/z calcd for $C_{18}H_{12}N_2O[M+H]^+$ 273.1022, found 273.1031.

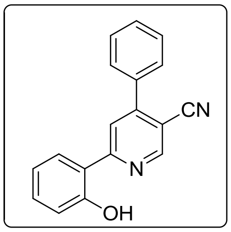

6-(2-Hydroxy-5-methoxyphenyl)-4-phenylnicotinonitrile(5ck): light yellow solid; mp 152-154 ºC; yield 74% (44.7 mg); R_f = 0.58 (EtOAc:hexane = 1:6); IR (KBr) ν 3425, 2227, 1596, 1529, 1488, 1401 cm^{-1}; ^1H NMR (400 MHz, $CDCl_3$) δ 13.77 (s, 1H), 9.14 (s, 1H), 8.36 (s, 1H), 8.33 (s, 1H), 7.79-7.81 (m, 2H), 7.53 (d, *J*= 8.6 Hz, 2H), 7.36-7.40 (m, 1H), 7.07 (d, *J* = 8.3 Hz, 1H), 6.91-6.94 (m, 1H), 3.78 (s, 3H) ppm; ^{13}C NMR (100 MHz, $CDCl_3$) δ 160.5, 154.8, 153.5, 152.5, 151.1, 135.6, 130.5, 129.3, 128.4, 120.2, 119.9, 119.3, 117.6, 116.5, 111.2, 106.0, 56.1 ppm; HRMS (ESI) m/z calcd for $C_{19}H_{14}N_2O_2[M+Na]^+$ 325.0947, found 325.0963.

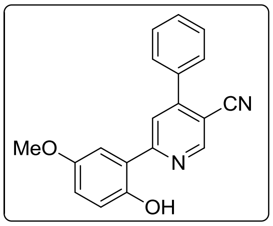

6-(2-Hydroxy-5-methoxyphenyl)-4-(4-methoxyphenyl)nicotinonitrile (5cl): light yellow solid ; mp 146-148 ºC; yield 68% (45.2 mg); R_f = 0.5 (EtOAc:hexane = 1:6); IR (KBr) ν 3442, 2223, 1609, 1588, 1494, 1369 cm^{-1}; ^1H NMR (400 MHz, $CDCl_3$) δ 12.92 (s, 1H), 8.83 (s, 1H), 7.91 (s, 1H), 7.65 (d, *J*

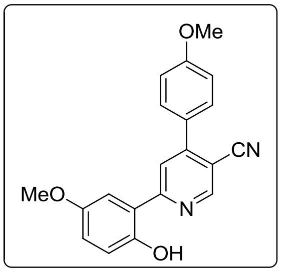

= 8.0 Hz, 2H), 7.30 (s, 1H), 7.10 (d, *J* = 8.0 Hz, 2H), 7.02 (m, 2H), 3.91 (s, 3H), 3.83 (s, 3H) ppm; ^{13}C NMR (100 MHz, $CDCl_3$) δ 159.0, 153.8, 153.0, 150.8, 150.4, 136.6, 132.2, 128.9, 125.4, 125.2, 121.3, 116.9, 116.4, 116.1, 112.7, 109.2, 56.0 (2C) ppm; HRMS (ESI) m/z calcd for $C_{20}H_{16}N_2O_3[M+H]^+$ 333.1234, found 333.1245.

4-(4-Fluorophenyl)-6-(2-hydroxyphenyl)nicotinonitrile (5am): light yellow solid; mp 140-142 ºC; yield 76% (44.0 mg); R_f = 0.57 (EtOAc:hexane = 1:6); IR (KBr) ν 3443, 2223, 1601, 1513, 1417, 1362 cm^{-1}; ^{1}H NMR (400 MHz, $CDCl_3$) δ 13.30 (s, 1H), 8.86 (s, 1H), 7.98 (s, 1H), 7.83 (d, *J* = 7.8 Hz, 1H), 7.66-7.668 (m, 2H), 7.42-7.38 (m, 1H), 7.30-7.28 (m, 2H), 7.07 (d, *J* = 8.0 Hz,1H), 6.97-6.94 (m, 1H) ppm; ^{13}C NMR (100 MHz, $CDCl_3$) ^{13}C NMR (100 MHz, $CDCl_3$) δ 164.1 (J_{C-F} = 250.8 Hz), 160.9, 160.7, 152.3, 151.1, 133.6, 131.7, 130.5 (J_{C-F} = 8.8 Hz), 126.9, 119.5, 119.2 (J_{C-F} = 21.0 Hz), 117.6, 116.7, 116.5, 116.4, 105.8 ppm; HRMS (ESI) m/z calcd for $C_{18}H_{11}FN_2O[M+H]^+$ 291.0928, found 291.0921.

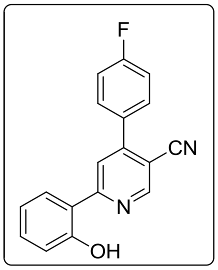

References

1. C. A. Osborne, T. B. D. Endean, E. R. Jarvo, *Org. Lett*. **2015**, *17*, 5340.
2. S. Kayal, S. Mukherjee, *Org. Lett*. **2017**, *19*, 4944.

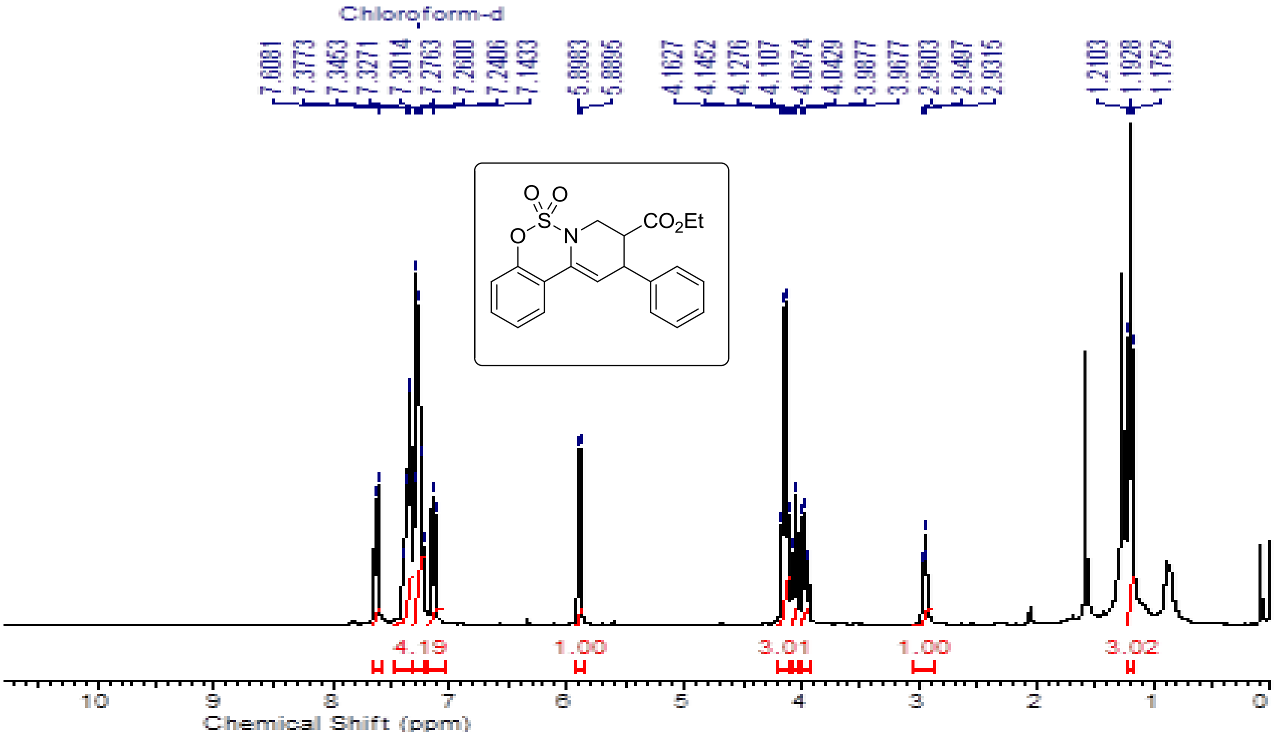
Chloroform-d
7.6081
7.3773
7.3453
7.3271
7.3014
7.2763
7.2600
7.2406
7.1433
5.8983
5.8895
4.1627
4.1452
4.1276
4.1107
4.0674
4.0429
3.9877
3.9677
2.9603
2.9407
2.9315
1.2103
1.1928
1.1752
CO2Et
4.19
1.00
3.01
1.00
3.02
Chemical Shift (ppm)

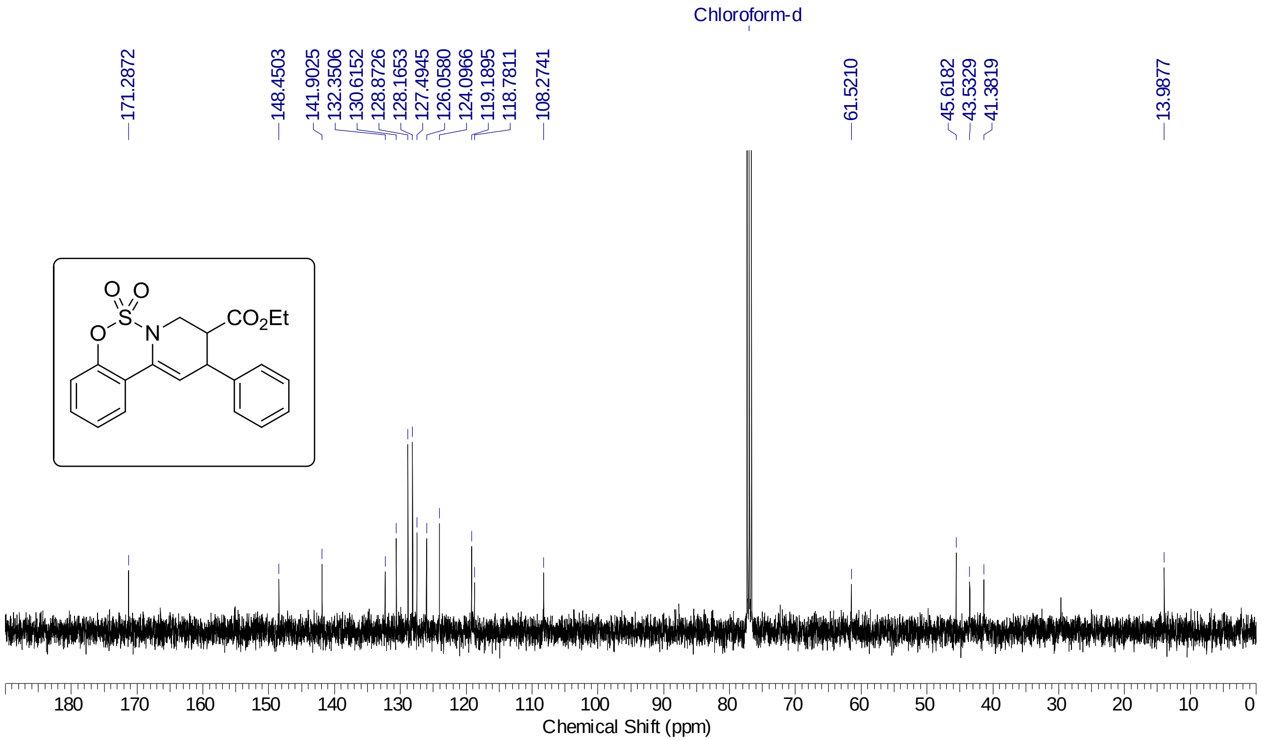
Chloroform-d
171.2872
148.4503
141.9025
132.3506
130.6152
128.8726
128.1653
127.4945
126.0580
124.0966
119.1895
118.7811
108.2741
61.5210
45.6182
43.5329
41.3819
13.9877
CO2Et
Chemical Shift (ppm)

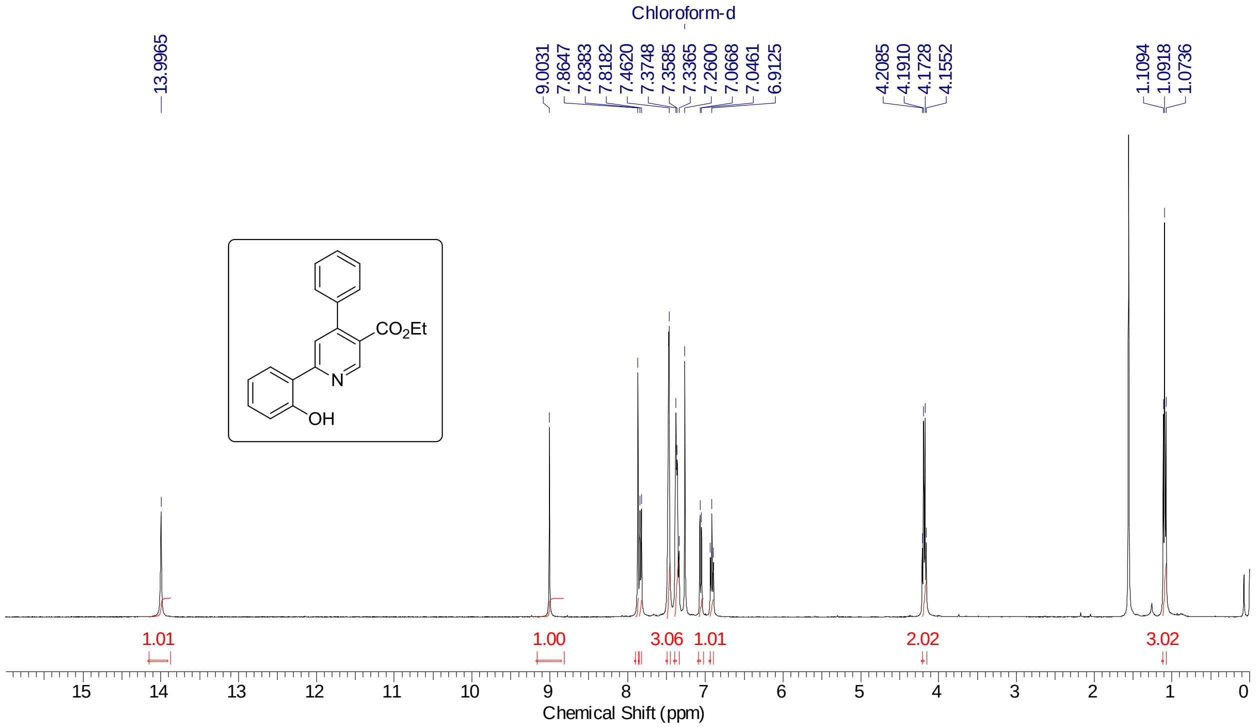
Chloroform-d
13.9965
9.0031
7.8647
7.8383
7.8182
7.4620
7.3748
7.3585
7.3365
7.2600
7.0668
7.0461
6.9125
4.2085
4.1910
4.1728
4.1552
1.1094
1.0918
1.0736
CO_2Et
N
OH
1.01
1.00
3.06
1.01
2.02
3.02
Chemical Shift (ppm)

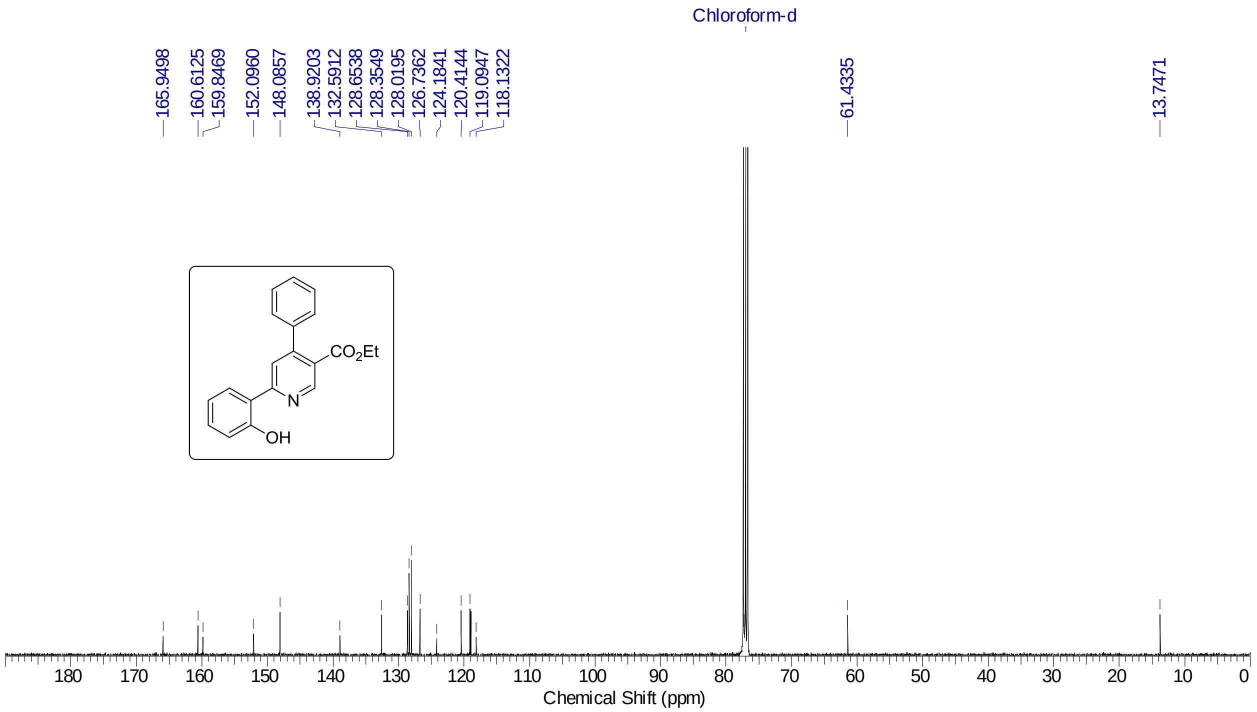
Chloroform-d
165.9498
160.6125
159.8469
152.0960
148.0857
138.9203
132.5912
128.6538
128.3549
128.0195
126.7362
124.1841
120.4144
119.0947
118.1322
61.4335
13.7471
CO_2Et
N
OH
Chemical Shift (ppm)

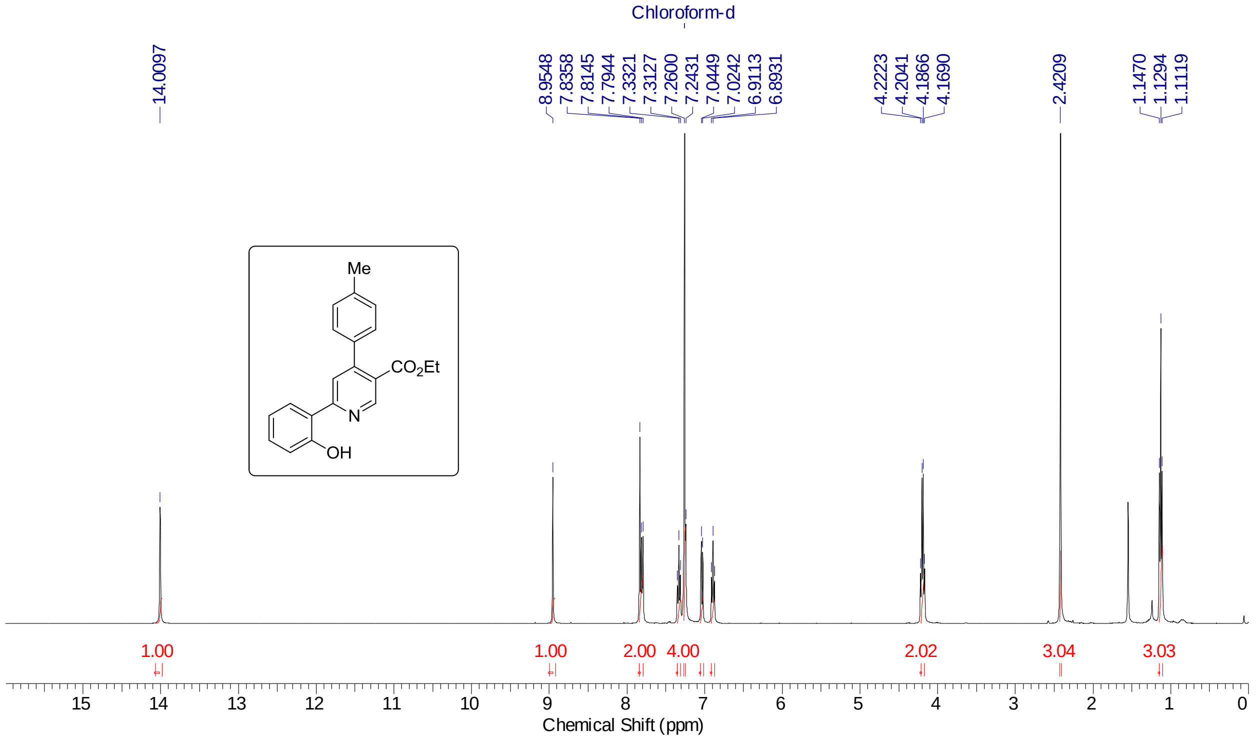
Chloroform-d
14.0097
8.9548
7.8358
7.8145
7.7944
7.3321
7.3127
7.2600
7.2431
7.0449
7.0242
6.9113
6.8931
4.2223
4.2041
4.1866
4.1690
2.4209
1.1470
1.1294
1.1119
Me
CO2Et
N
OH
1.00
1.00
2.00
4.00
2.02
3.04
3.03
Chemical Shift (ppm)

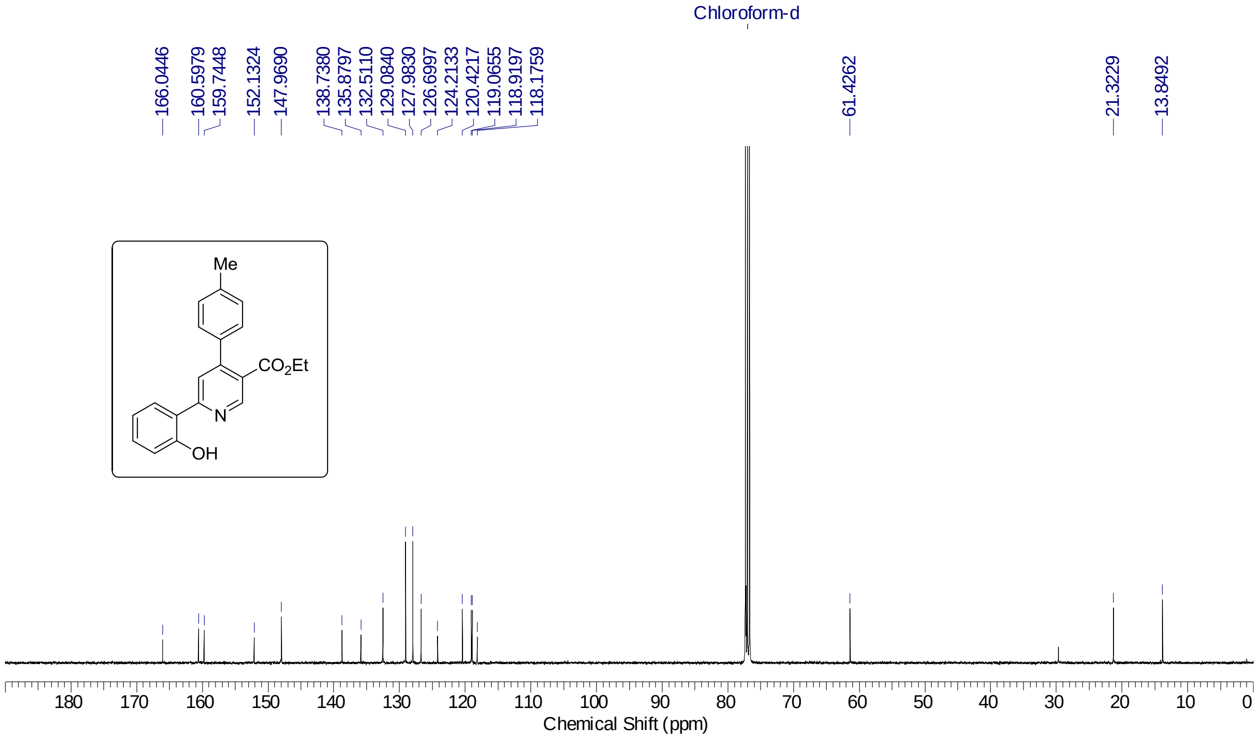
Chloroform-d
166.0446
160.5979
159.7448
152.1324
147.9690
138.7380
135.8797
132.5110
129.0840
127.9830
126.6997
124.2133
120.4217
119.0655
118.9197
118.1759
61.4262
21.3229
13.8492
Me
CO2Et
N
OH
Chemical Shift (ppm)

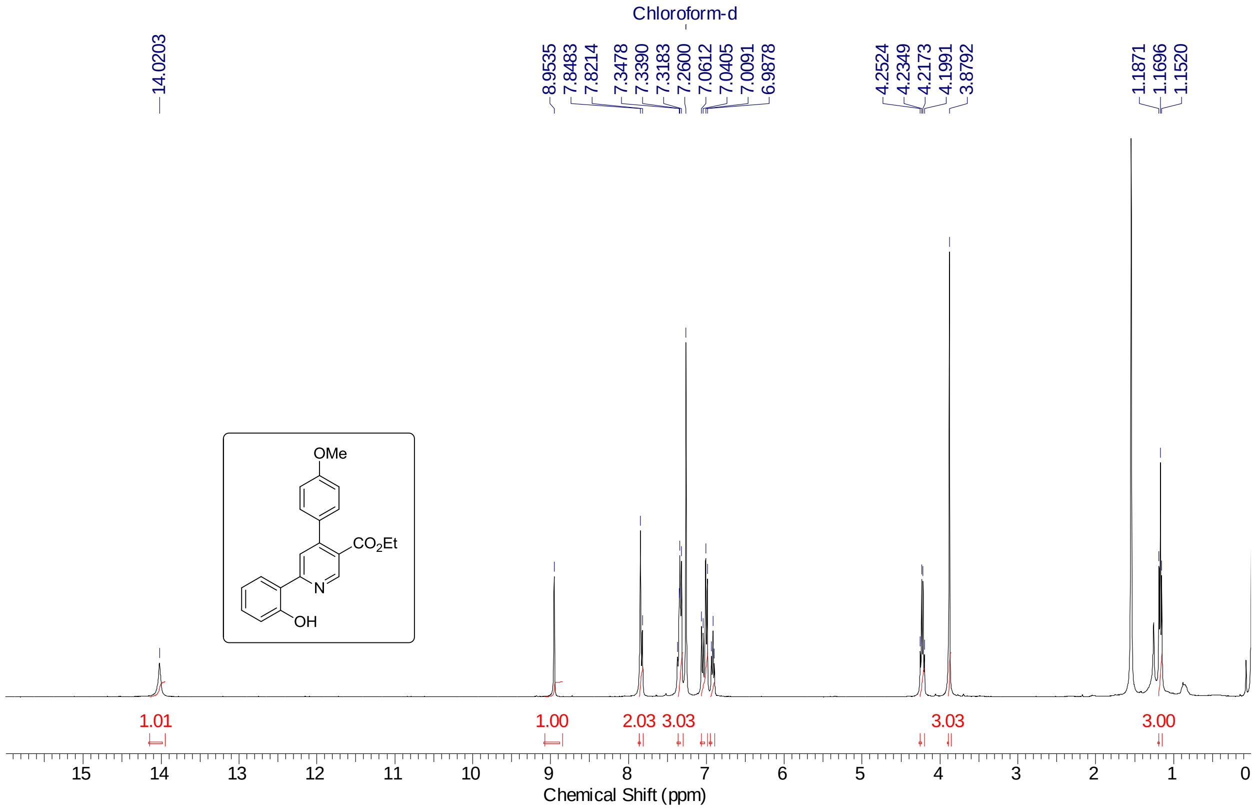

Chloroform-d
14.0203
8.9535
7.8483
7.8214
7.3478
7.3390
7.3183
7.2600
7.0612
7.0405
7.0091
6.9878
4.2524
4.2349
4.2173
4.1991
3.8792
1.1871
1.1696
1.1520
OMe
CO_2Et
N
OH
1.01
1.00
2.03
3.03
3.03
3.00
15
14
13
12
11
10
9
8
7
6
5
4
3
2
1
0
Chemical Shift (ppm)

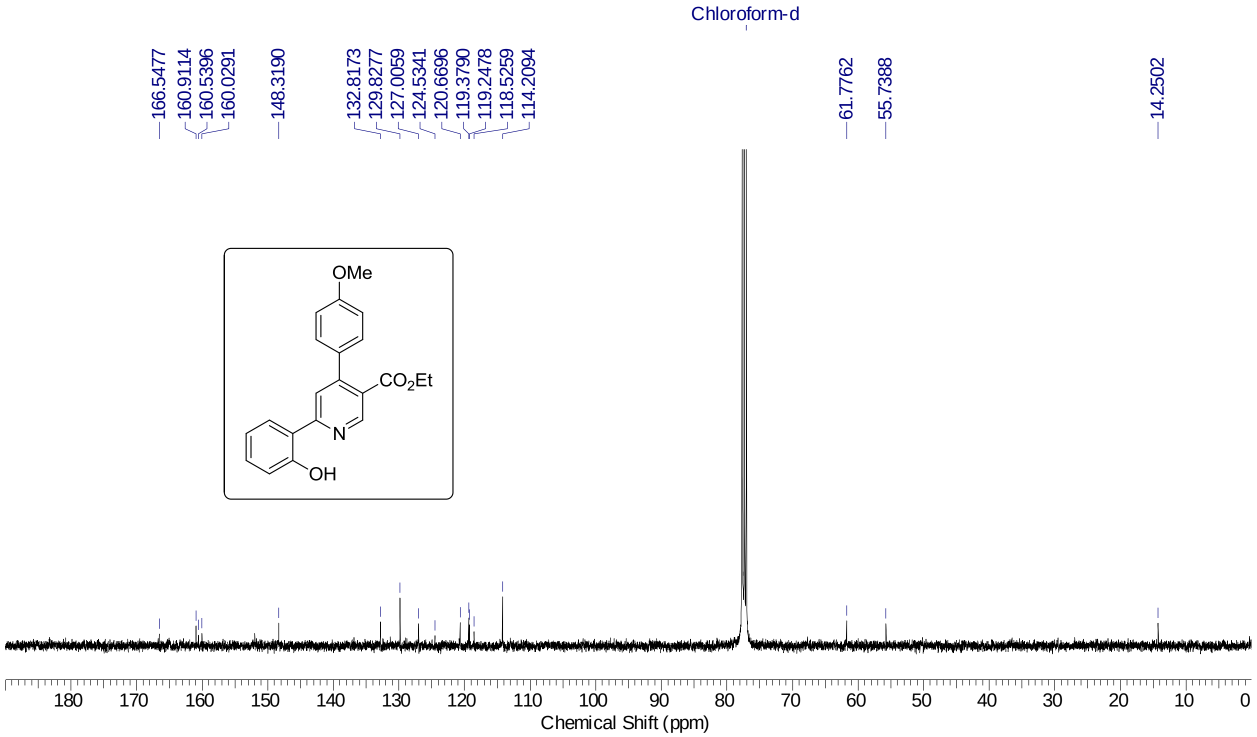

Chloroform-d
166.5477
160.9114
160.5396
160.0291
148.3190
132.8173
129.8277
127.0059
124.5341
120.6696
119.3790
119.2478
118.5259
114.2094
61.7762
55.7388
14.2502
OMe
CO_2Et
N
OH
180
170
160
150
140
130
120
110
100
90
80
70
60
50
40
30
20
10
0
Chemical Shift (ppm)

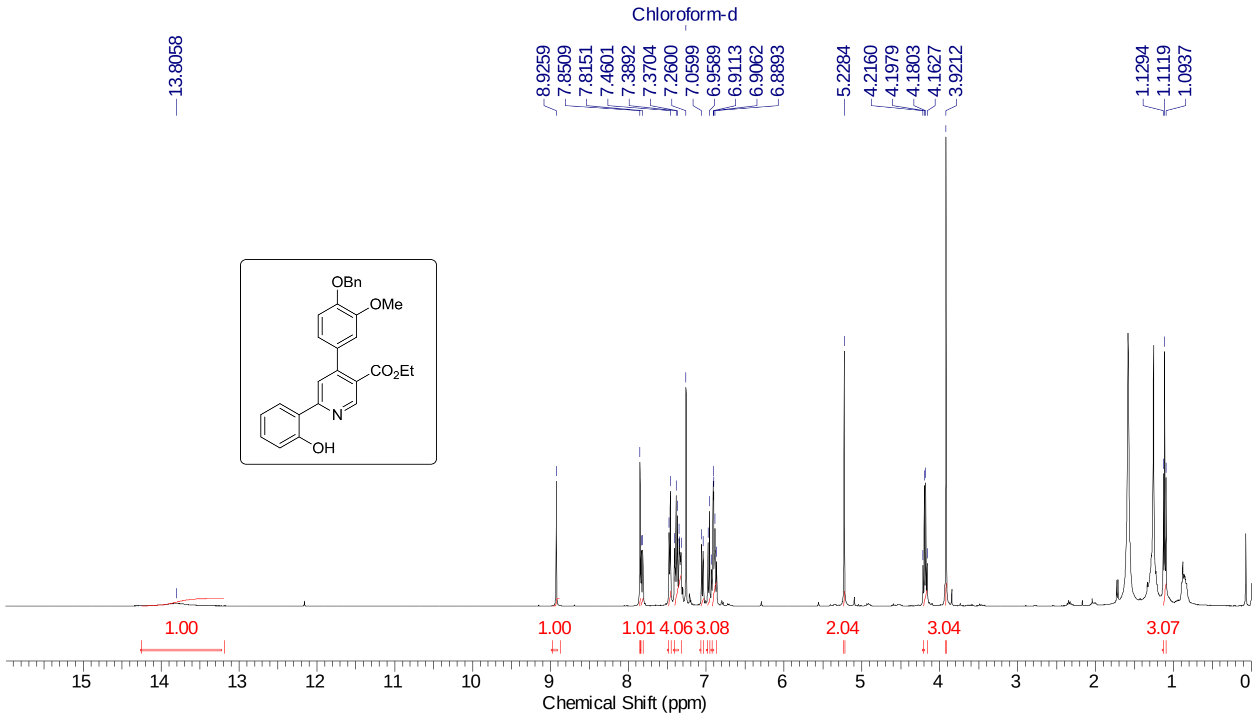
Chloroform-d
13.8058
8.9259
7.8509
7.8151
7.4601
7.3892
7.3704
7.2600
7.0599
6.9589
6.9113
6.9062
6.8893
5.2284
4.2160
4.1979
4.1803
4.1627
3.9212
1.1294
1.1119
1.0937
OBn
OMe
CO_2Et
N
OH
1.00
1.00
1.01
4.06
3.08
2.04
3.04
3.07
Chemical Shift (ppm)

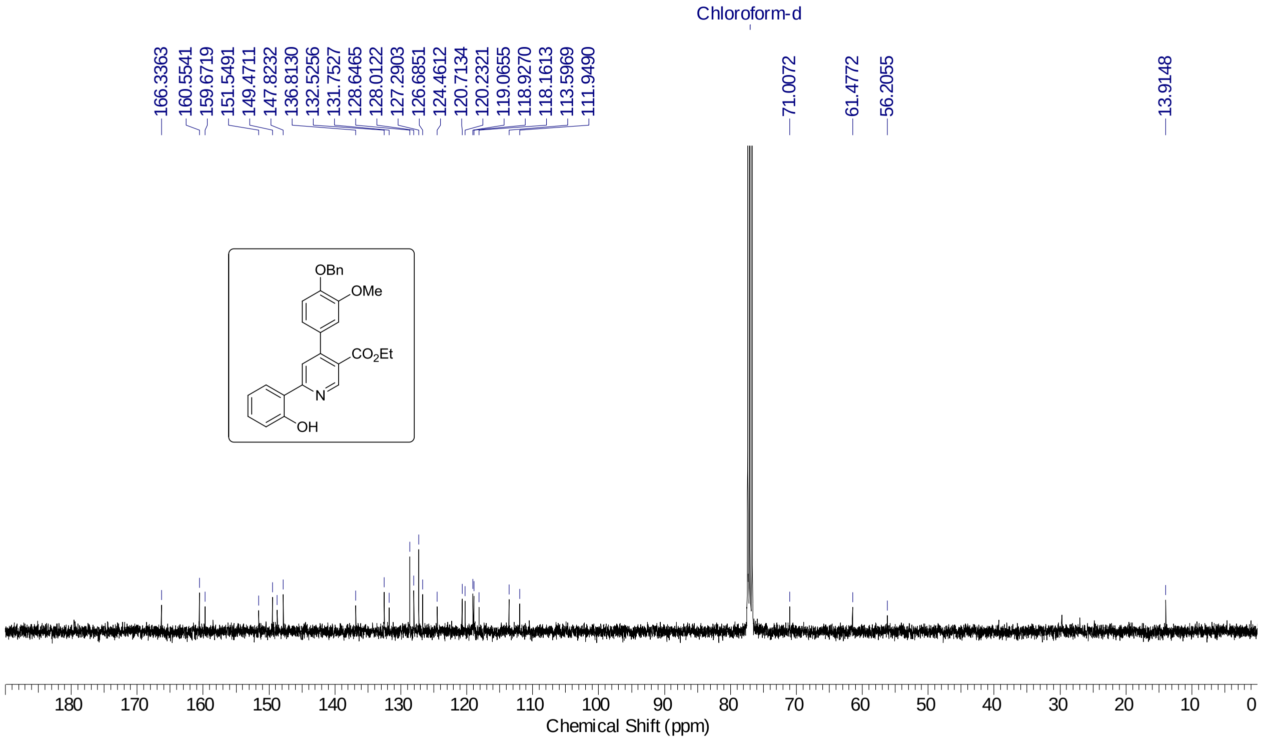
Chloroform-d
166.3363
160.5541
159.6719
151.5491
149.4711
147.8232
136.8130
132.5256
131.7527
128.6465
128.0122
127.2903
126.6851
124.4612
120.7134
120.2321
119.0655
118.9270
118.1613
113.5969
111.9490
71.0072
61.4772
56.2055
13.9148
OBn
OMe
CO_2Et
N
OH
Chemical Shift (ppm)

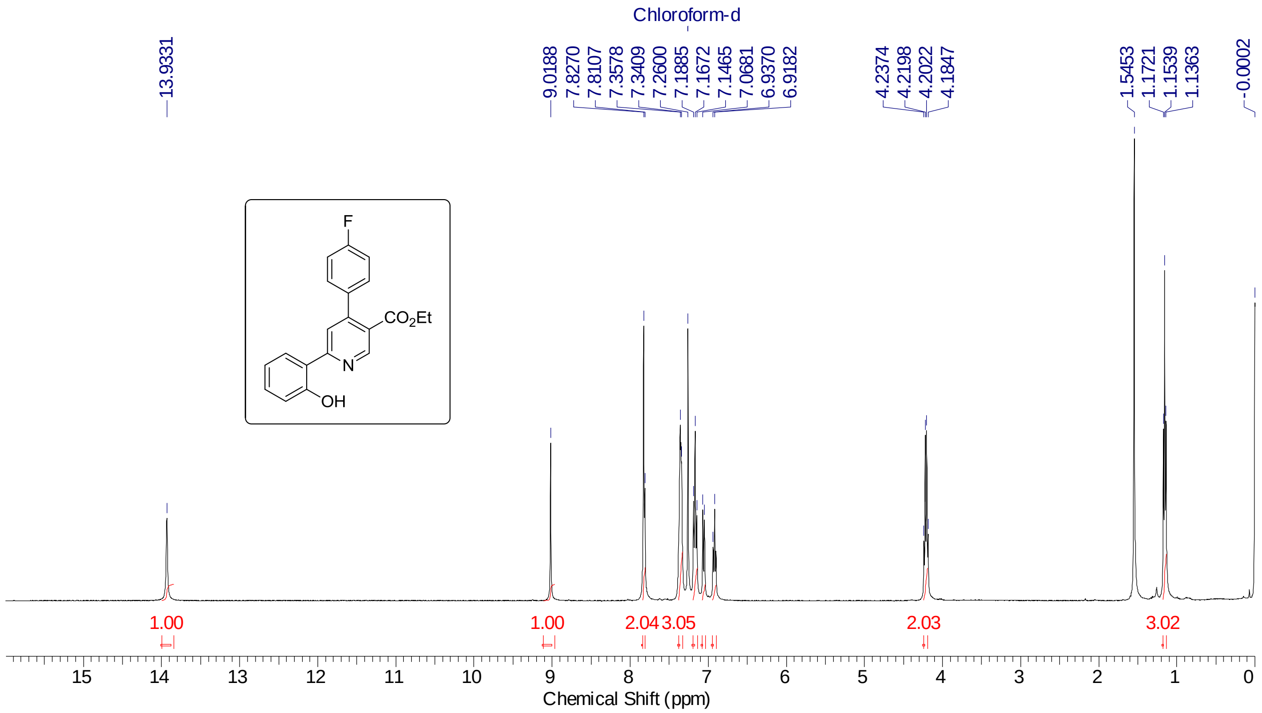

Chloroform-d
13.9331
9.0188
7.8270
7.8107
7.3578
7.3409
7.2600
7.1885
7.1672
7.1465
7.0681
6.9370
6.9182
4.2374
4.2198
4.2022
4.1847
1.5453
1.1721
1.1539
1.1363
-0.0002
F
CO_2Et
N
OH
1.00
1.00
2.04
3.05
2.03
3.02
Chemical Shift (ppm)

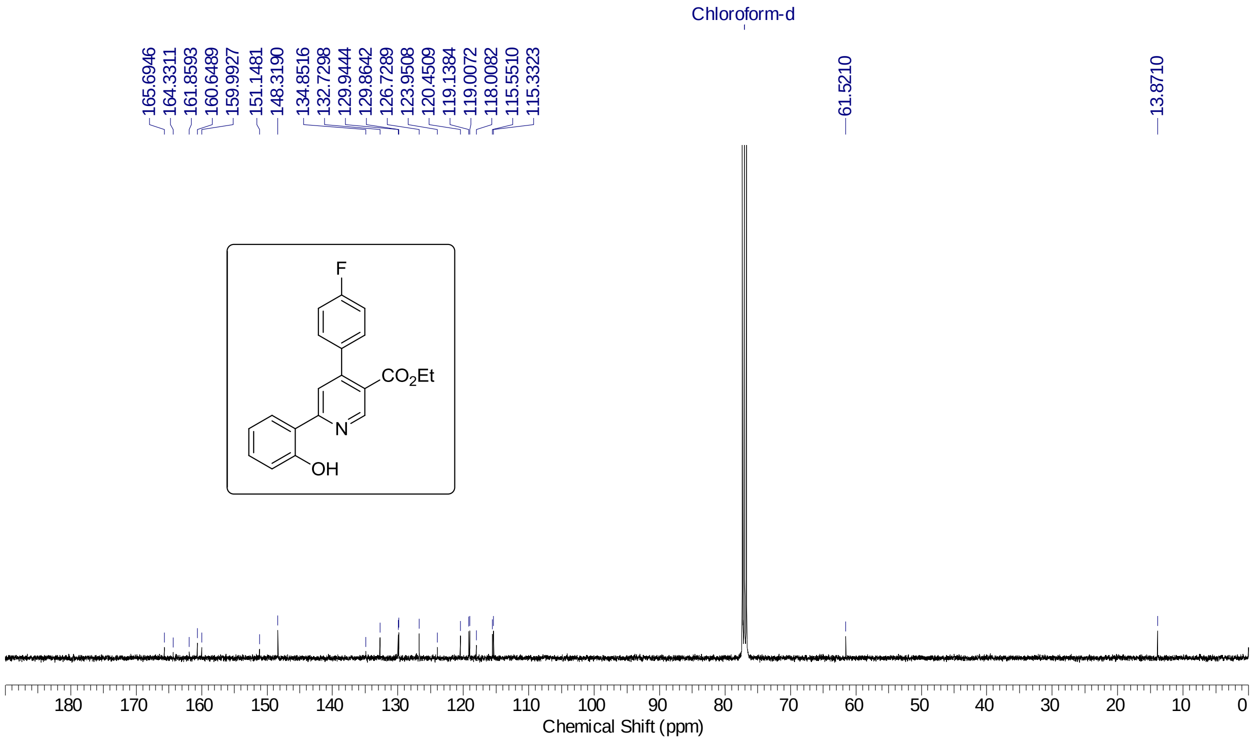

Chloroform-d
165.6946
164.3311
161.8593
160.6489
159.9927
151.1481
148.3190
134.8516
132.7298
129.9444
129.8642
126.7289
123.9508
120.4509
119.1384
119.0072
118.0082
115.5510
115.3323
61.5210
13.8710
F
CO_2Et
N
OH
Chemical Shift (ppm)

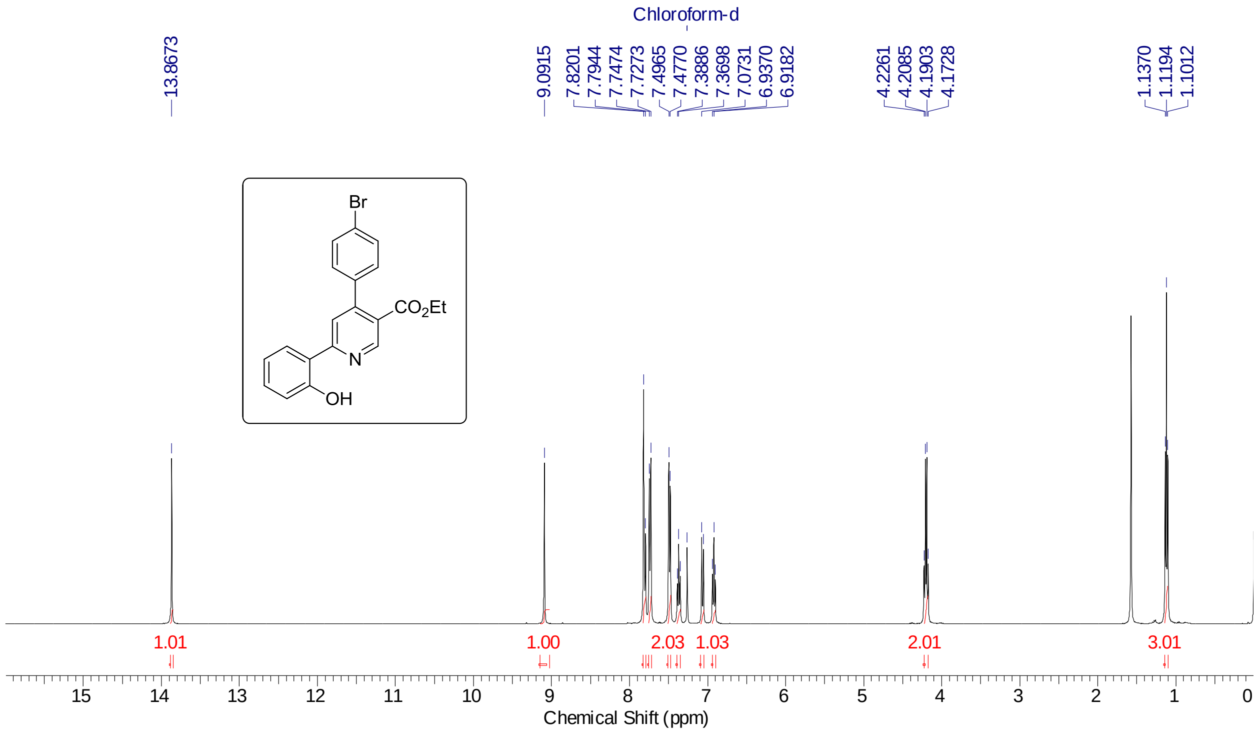
Chloroform-d
13.8673
9.0915
7.8201
7.7944
7.7474
7.7273
7.4965
7.4770
7.3886
7.3698
7.0731
6.9370
6.9182
4.2261
4.2085
4.1903
4.1728
1.1370
1.1194
1.1012
Br
CO_2Et
N
OH
1.01
1.00
2.03
1.03
2.01
3.01
15 14 13 12 11 10 9 8 7 6 5 4 3 2 1 0
Chemical Shift (ppm)

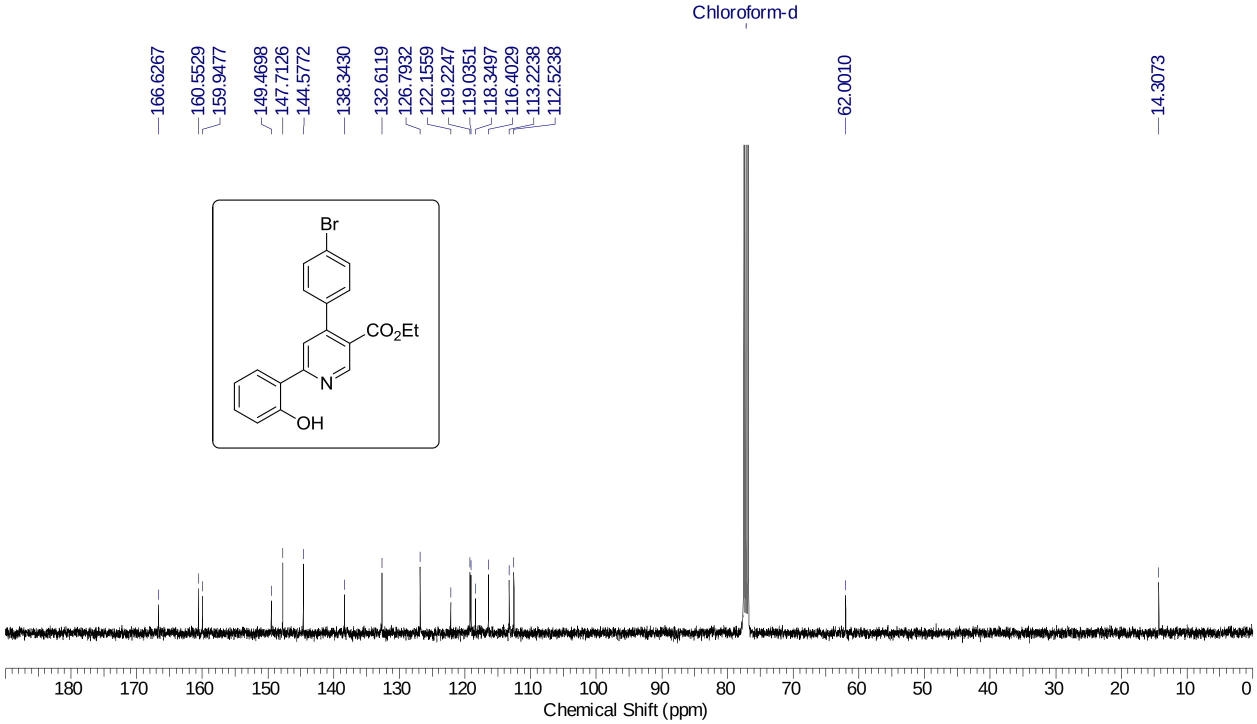
Chloroform-d
166.6267
160.5529
159.9477
149.4698
147.7126
144.5772
138.3430
132.6119
126.7932
122.1559
119.2247
119.0351
118.3497
116.4029
113.2238
112.5238
62.0010
14.3073
Br
CO_2Et
N
OH
180 170 160 150 140 130 120 110 100 90 80 70 60 50 40 30 20 10 0
Chemical Shift (ppm)

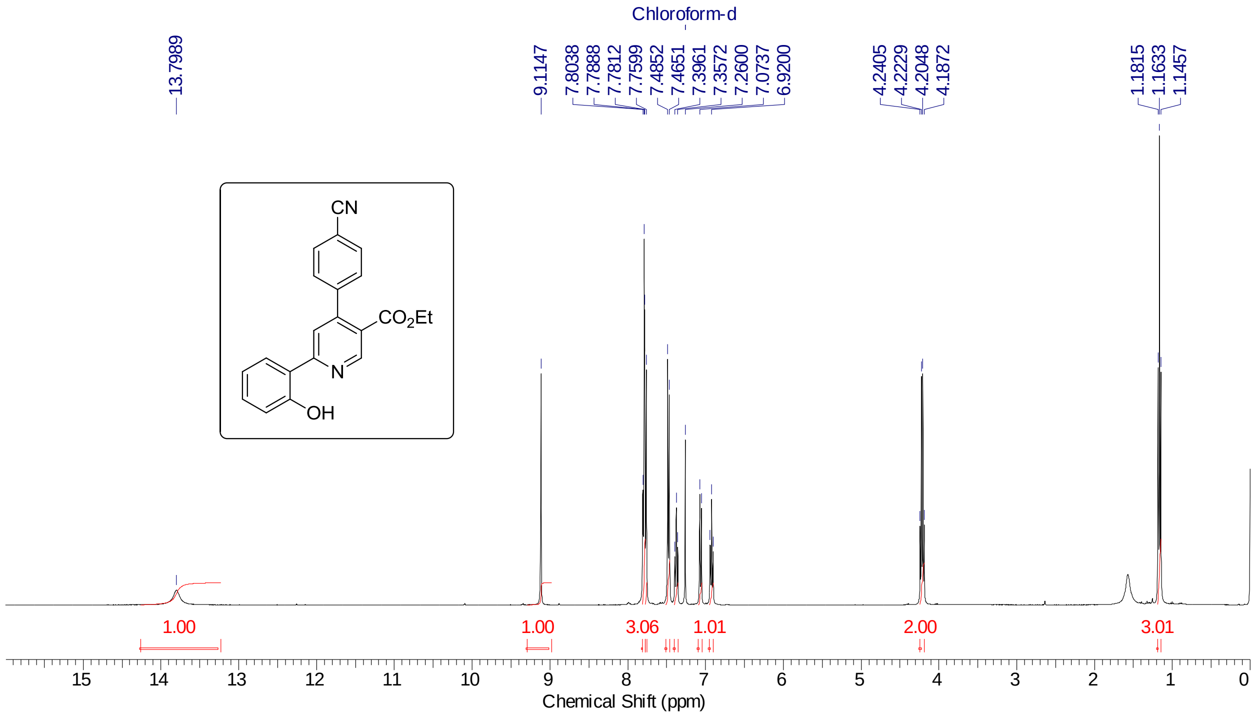
Chloroform-d
13.7989
9.1147
7.8038
7.7888
7.7812
7.7599
7.4852
7.4651
7.3961
7.3572
7.2600
7.0737
6.9200
4.2405
4.2229
4.2048
4.1872
1.1815
1.1633
1.1457
CN
CO_2Et
N
OH
1.00
1.00
3.06
1.01
2.00
3.01
Chemical Shift (ppm)

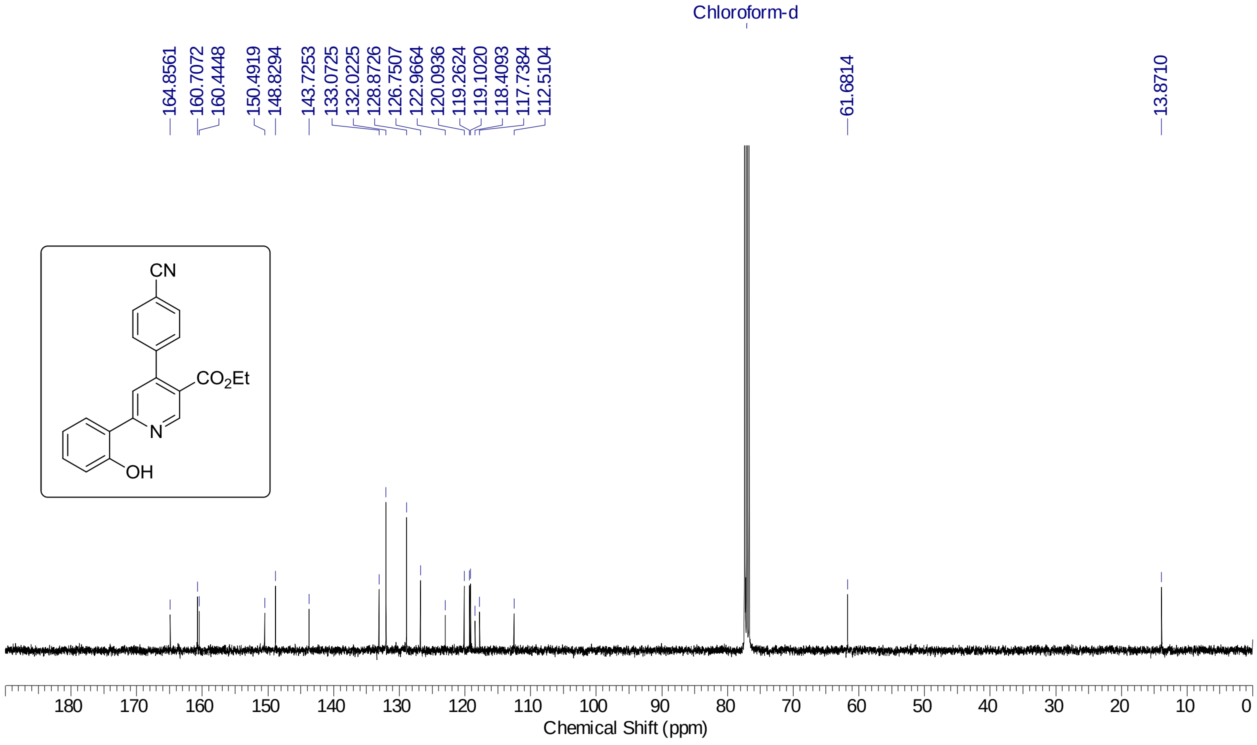
Chloroform-d
164.8561
160.7072
160.4448
150.4919
148.8294
143.7253
133.0725
132.0225
128.8726
126.7507
122.9664
120.0936
119.2624
119.1020
118.4093
117.7384
112.5104
61.6814
13.8710
CN
CO_2Et
N
OH
Chemical Shift (ppm)

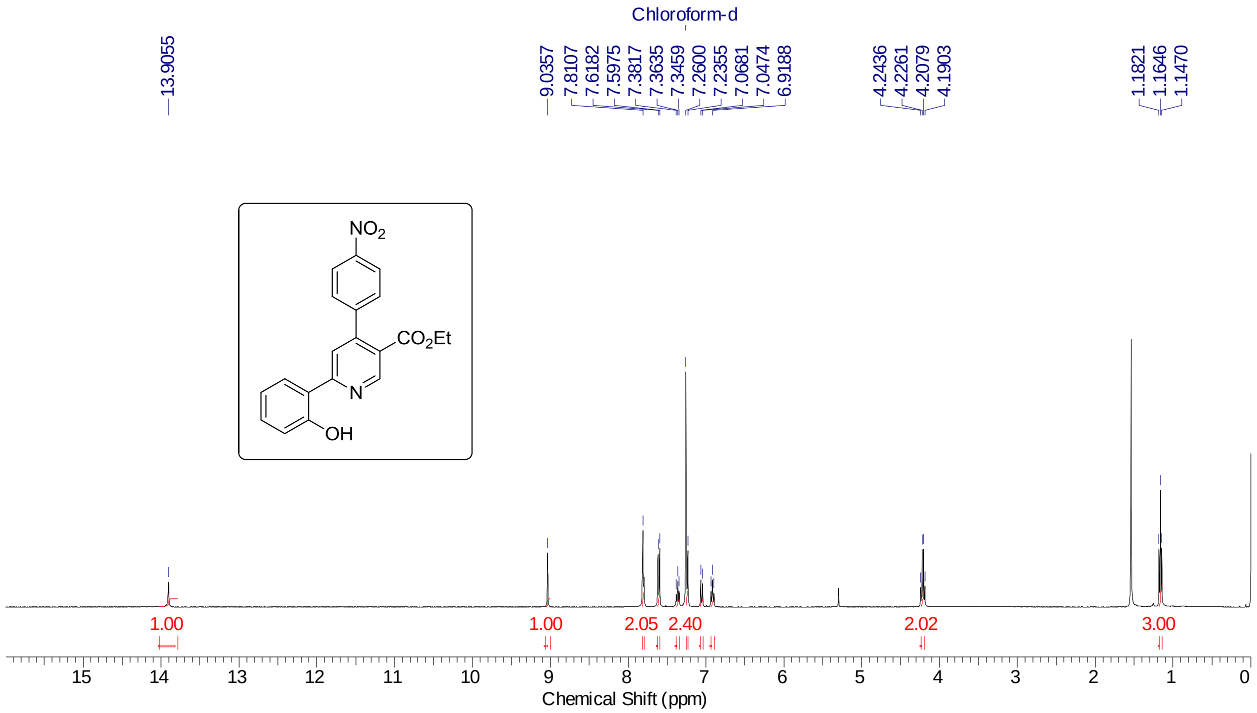
Chloroform-d
13.9055
9.0357
7.8107
7.6182
7.5975
7.3817
7.3635
7.3459
7.2600
7.2355
7.0681
7.0474
6.9188
4.2436
4.2261
4.2079
4.1903
1.1821
1.1646
1.1470
NO_2
CO_2Et
N
OH
1.00
1.00
2.05
2.40
2.02
3.00
Chemical Shift (ppm)

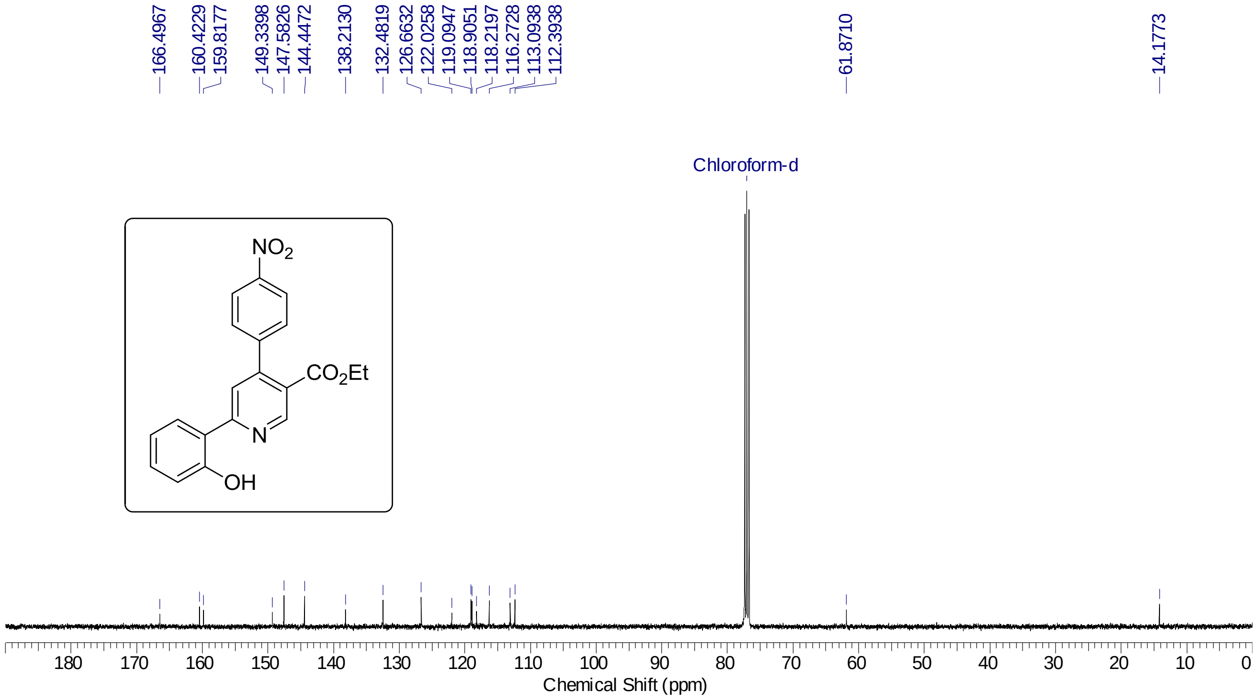
166.4967
160.4229
159.8177
149.3398
147.5826
144.4472
138.2130
132.4819
126.6632
122.0258
119.0947
118.9051
118.2197
116.2728
113.0938
112.3938
61.8710
14.1773
Chloroform-d
NO_2
CO_2Et
N
OH
Chemical Shift (ppm)

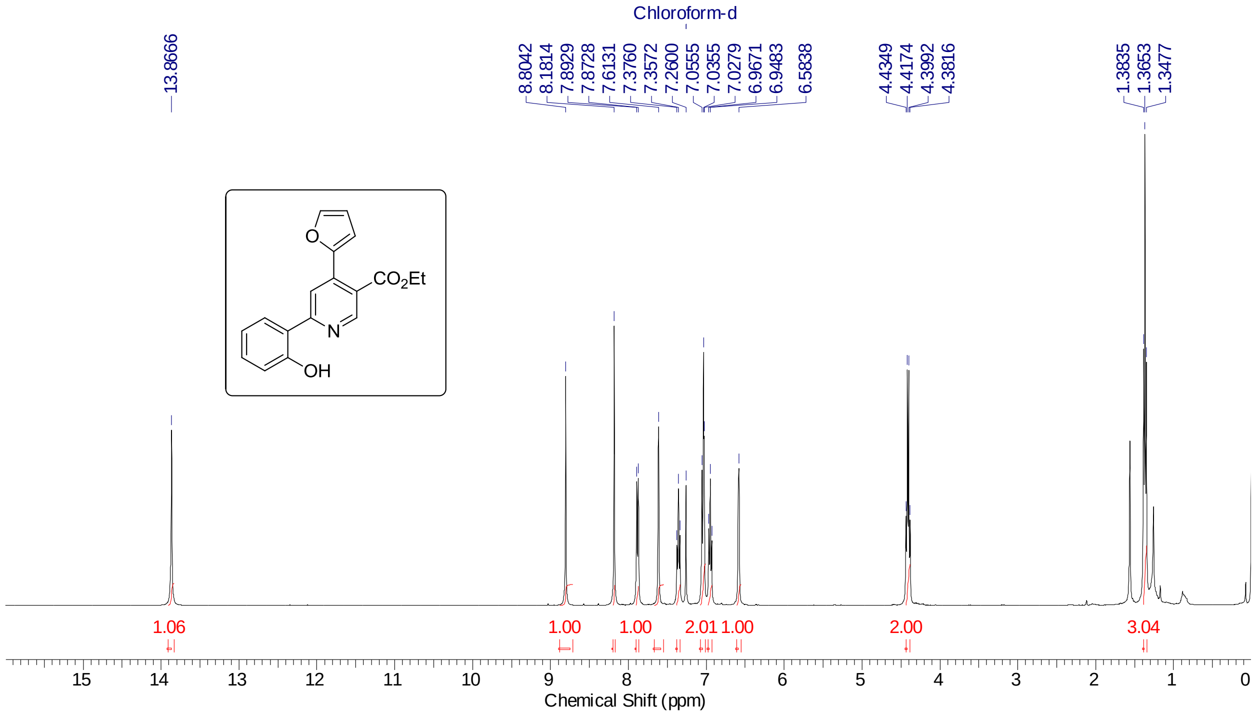
Chloroform-d
13.8666
8.8042
8.1814
7.8929
7.8728
7.6131
7.3760
7.3572
7.2600
7.0555
7.0355
7.0279
6.9671
6.9483
6.5838
4.4349
4.4174
4.3992
4.3816
1.3835
1.3653
1.3477
CO2Et
O
N
OH
1.06
1.00
1.00
2.01
1.00
2.00
3.04
15 14 13 12 11 10 9 8 7 6 5 4 3 2 1 0
Chemical Shift (ppm)

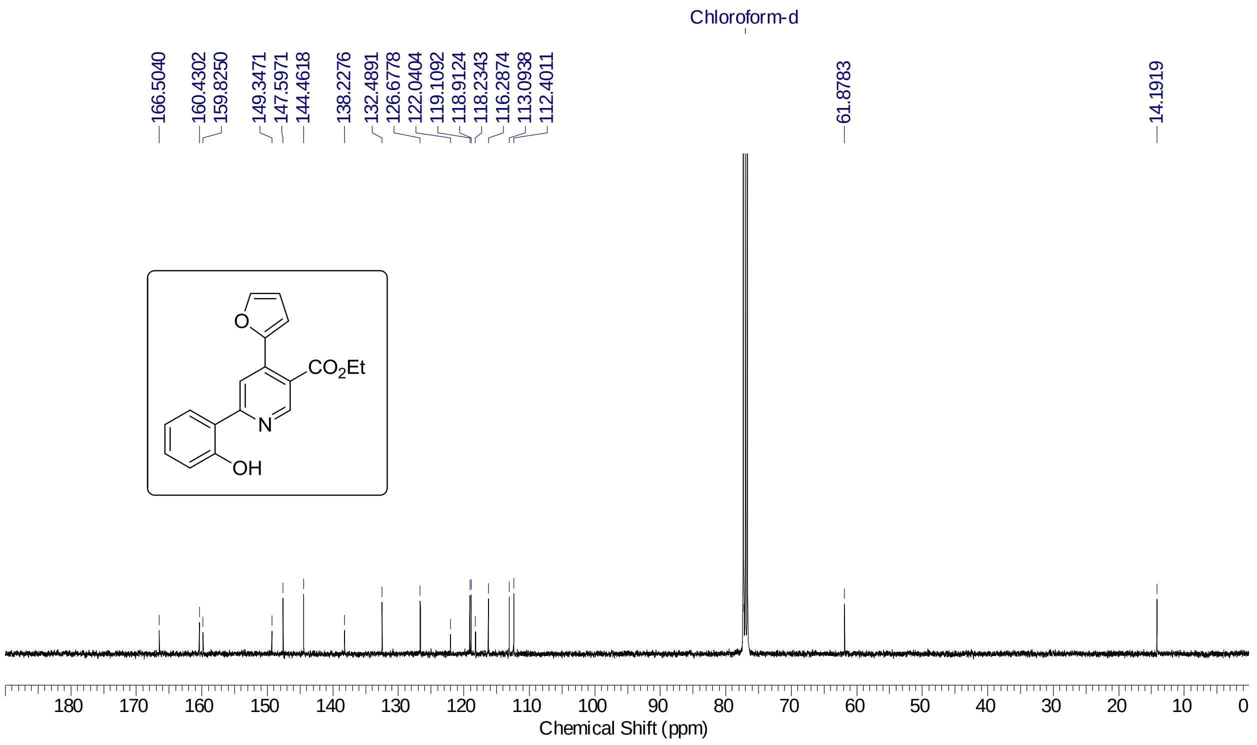
Chloroform-d
166.5040
160.4302
159.8250
149.3471
147.5971
144.4618
138.2276
132.4891
126.6778
122.0404
119.1092
118.9124
118.2343
116.2874
113.0938
112.4011
61.8783
14.1919
CO2Et
O
N
OH
180 170 160 150 140 130 120 110 100 90 80 70 60 50 40 30 20 10 0
Chemical Shift (ppm)

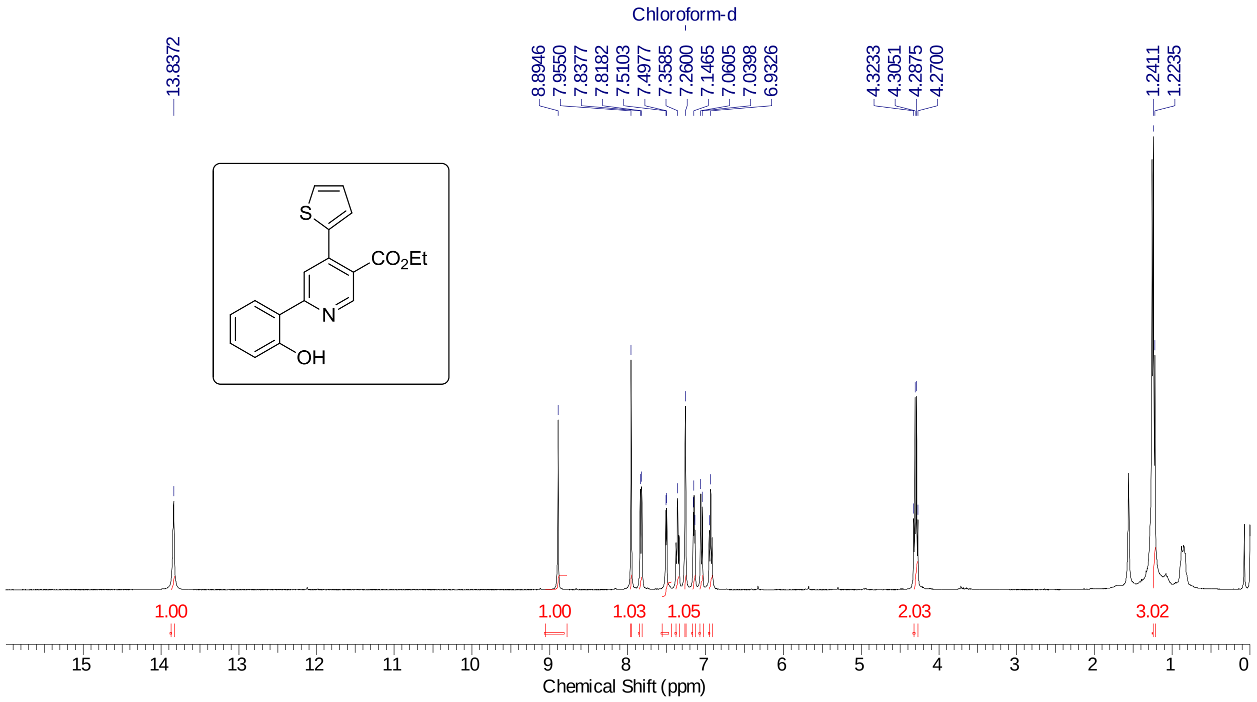
Chloroform-d
13.8372
8.8946
7.9550
7.8377
7.8182
7.5103
7.4977
7.3585
7.2600
7.1465
7.0605
7.0398
6.9326
4.3233
4.3051
4.2875
4.2700
1.2411
1.2235
S
CO2Et
N
OH
1.00
1.00
1.03
1.05
2.03
3.02
15
14
13
12
11
10
9
8
7
6
5
4
3
2
1
0
Chemical Shift (ppm)

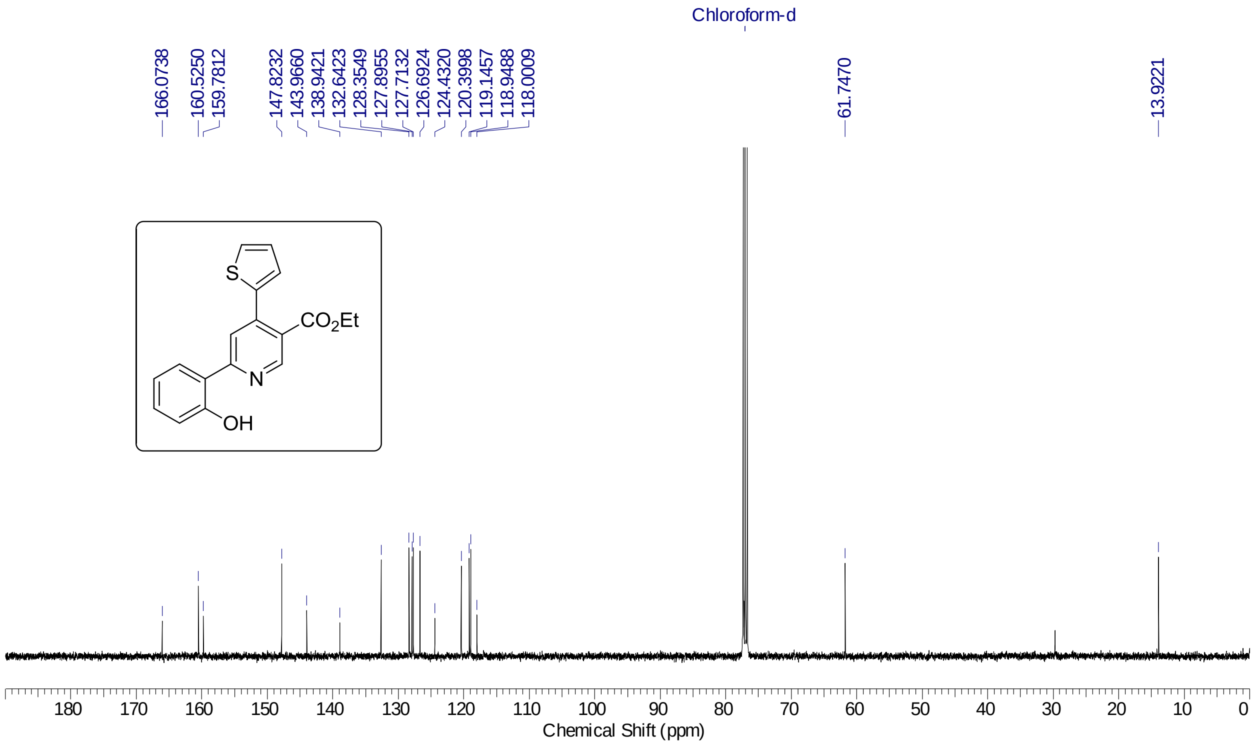
Chloroform-d
166.0738
160.5250
159.7812
147.8232
143.9660
138.9421
132.6423
128.3549
127.8955
127.7132
126.6924
124.4320
120.3998
119.1457
118.9488
118.0009
61.7470
13.9221
S
CO2Et
N
OH
180
170
160
150
140
130
120
110
100
90
80
70
60
50
40
30
20
10
0
Chemical Shift (ppm)

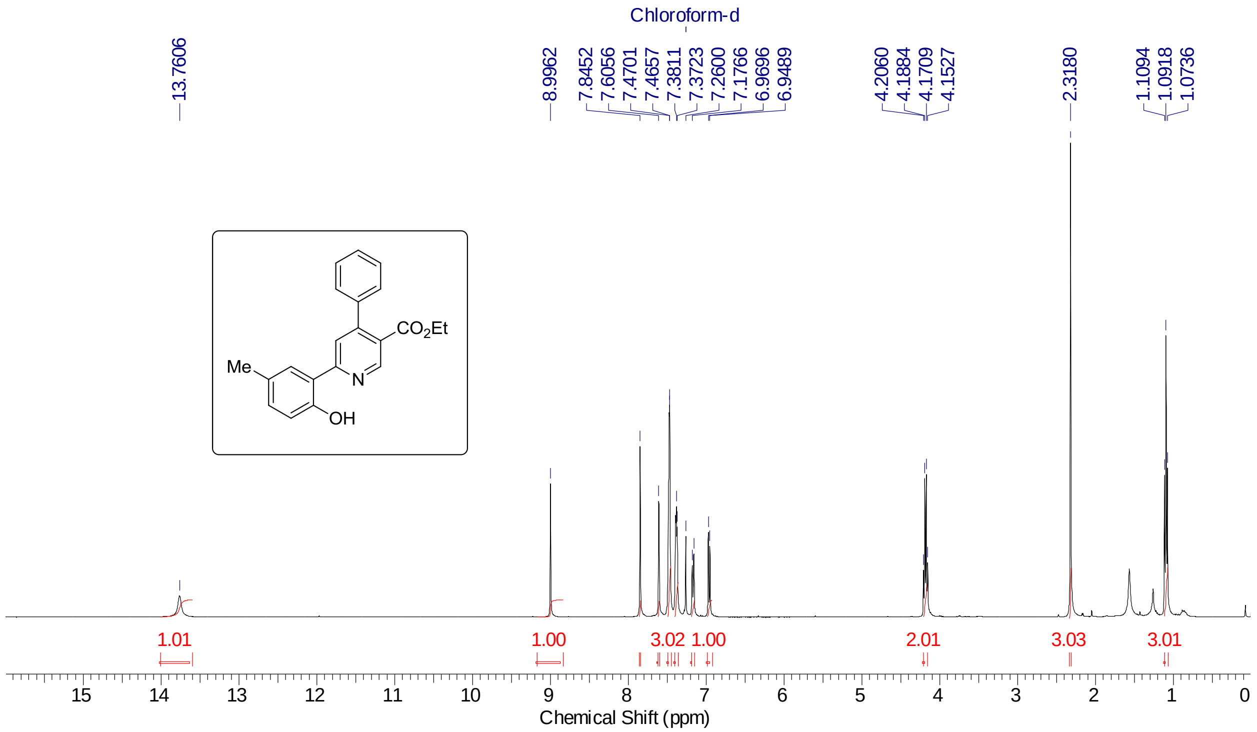

Chloroform-d
13.7606
8.9962
7.8452
7.6056
7.4701
7.4657
7.3811
7.3723
7.2600
7.1766
6.9696
6.9489
4.2060
4.1884
4.1709
4.1527
2.3180
1.1094
1.0918
1.0736
CO2Et
Me
N
OH
1.01
1.00
3.02
1.00
2.01
3.03
3.01
Chemical Shift (ppm)

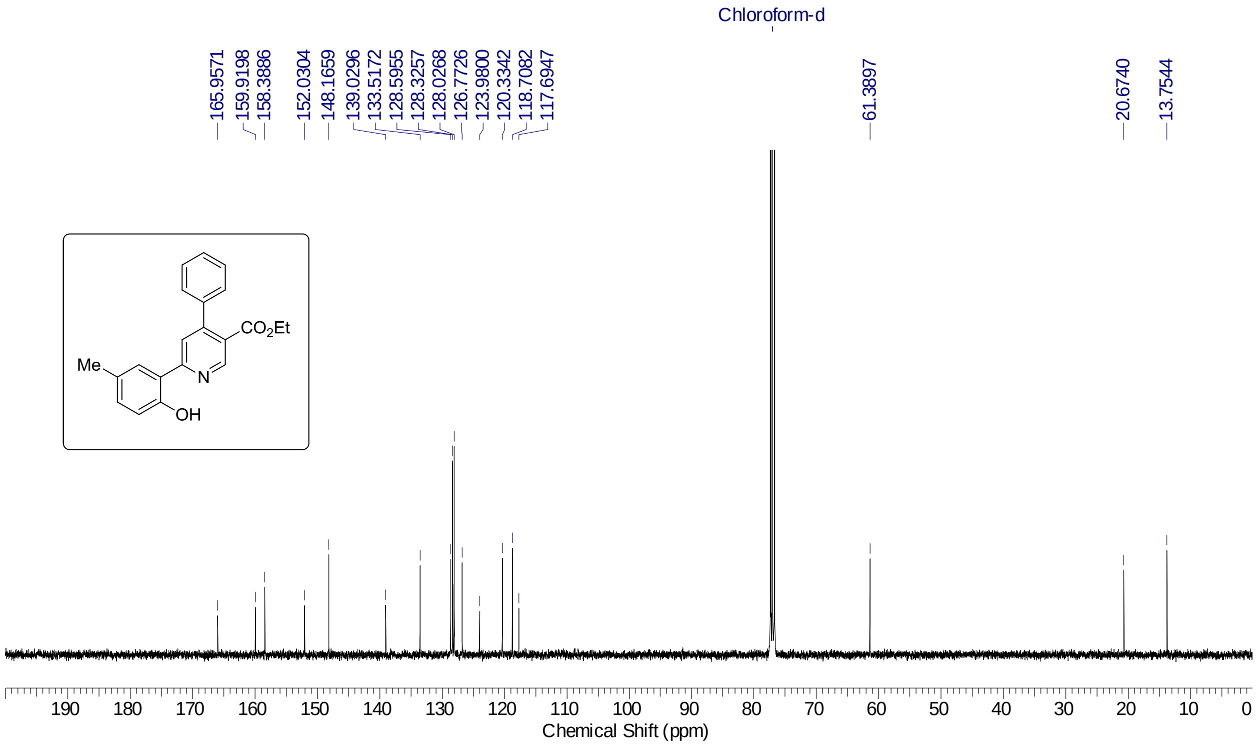

Chloroform-d
165.9571
159.9198
158.3886
152.0304
148.1659
139.0296
133.5172
128.5955
128.3257
128.0268
126.7726
123.9800
120.3342
118.7082
117.6947
61.3897
20.6740
13.7544
CO2Et
Me
N
OH
Chemical Shift (ppm)

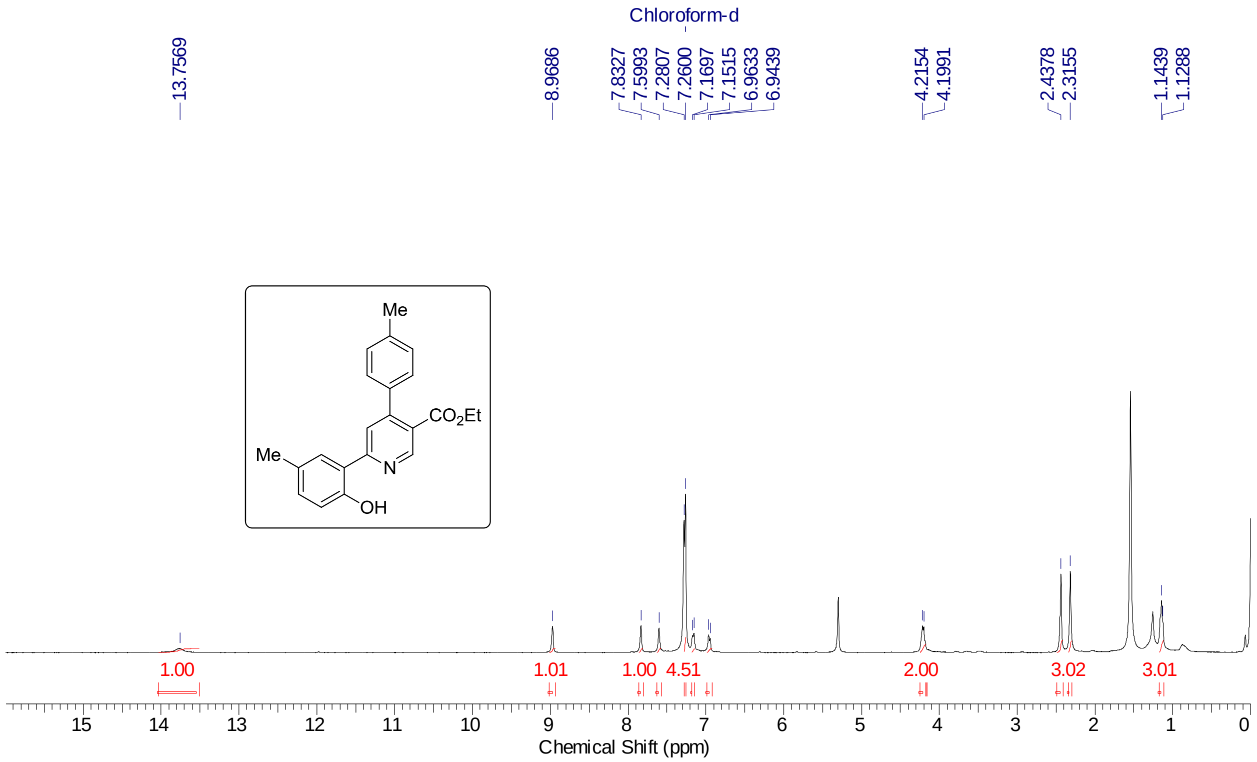
Chloroform-d
13.7569
8.9686
7.8327
7.5993
7.2807
7.2600
7.1697
7.1515
6.9633
6.9439
4.2154
4.1991
2.4378
2.3155
1.1439
1.1288
Me
CO2Et
Me
N
OH
1.00
1.01
1.00
4.51
2.00
3.02
3.01
Chemical Shift (ppm)

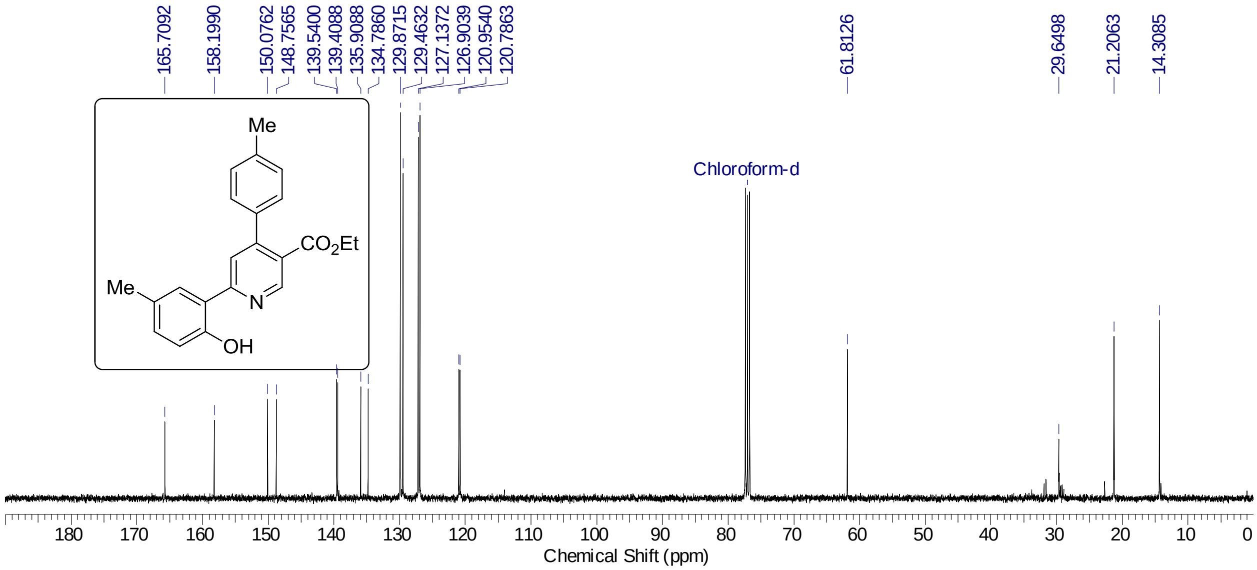
165.7092
158.1990
150.0762
148.7565
139.5400
139.4088
135.9088
134.7860
129.8715
129.4632
127.1372
126.9039
120.9540
120.7863
61.8126
29.6498
21.2063
14.3085
Chloroform-d
Me
CO2Et
Me
N
OH
Chemical Shift (ppm)

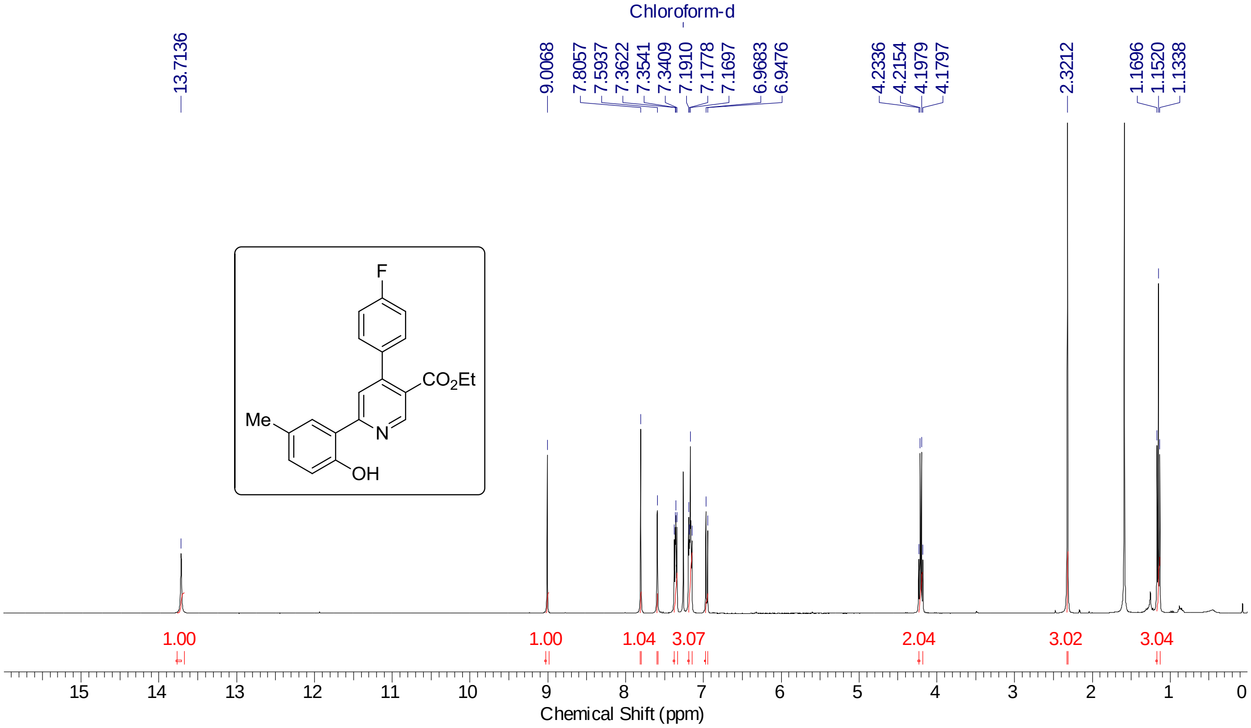
Chloroform-d
13.7136
9.0068
7.8057
7.5937
7.3622
7.3541
7.3409
7.1910
7.1778
7.1697
6.9683
6.9476
4.2336
4.2154
4.1979
4.1797
2.3212
1.1696
1.1520
1.1338
F
CO2Et
Me
N
OH
1.00
1.00
1.04
3.07
2.04
3.02
3.04
Chemical Shift (ppm)

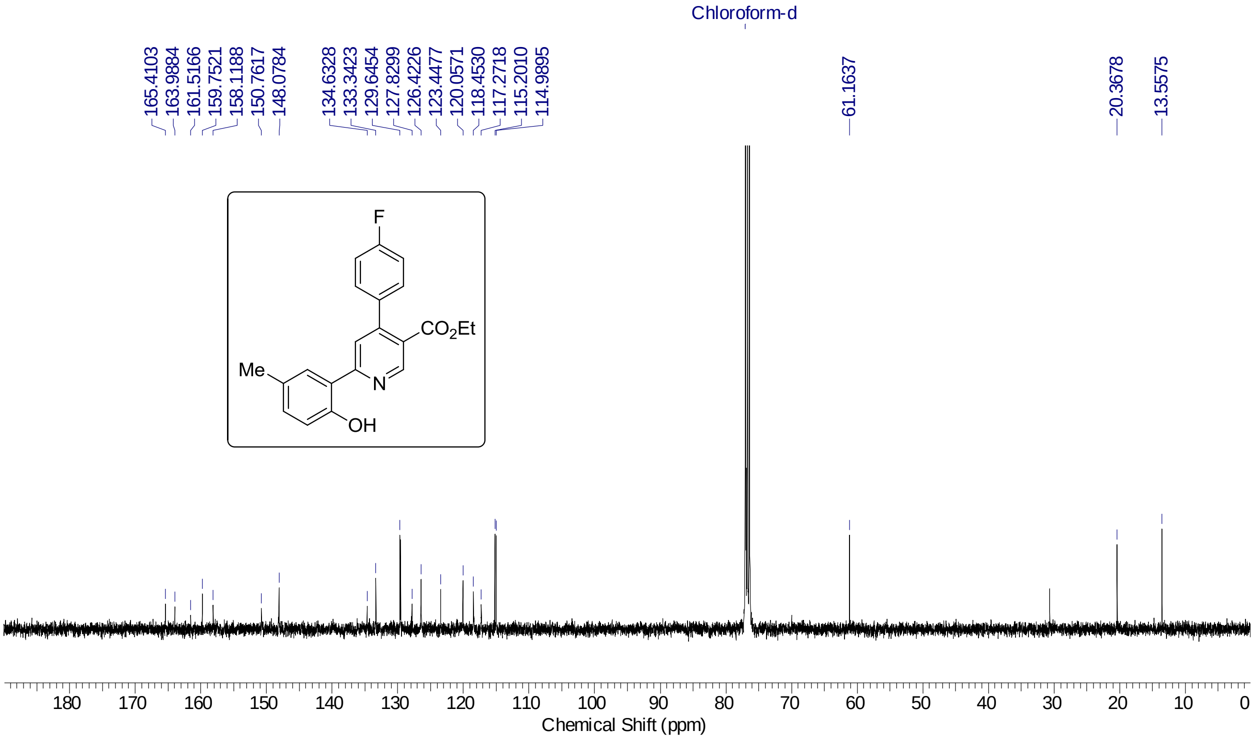
Chloroform-d
165.4103
163.9884
161.5166
159.7521
158.1188
150.7617
148.0784
134.6328
133.3423
129.6454
127.8299
126.4226
123.4477
120.0571
118.4530
117.2718
115.2010
114.9895
61.1637
20.3678
13.5575
F
CO2Et
Me
N
OH
Chemical Shift (ppm)

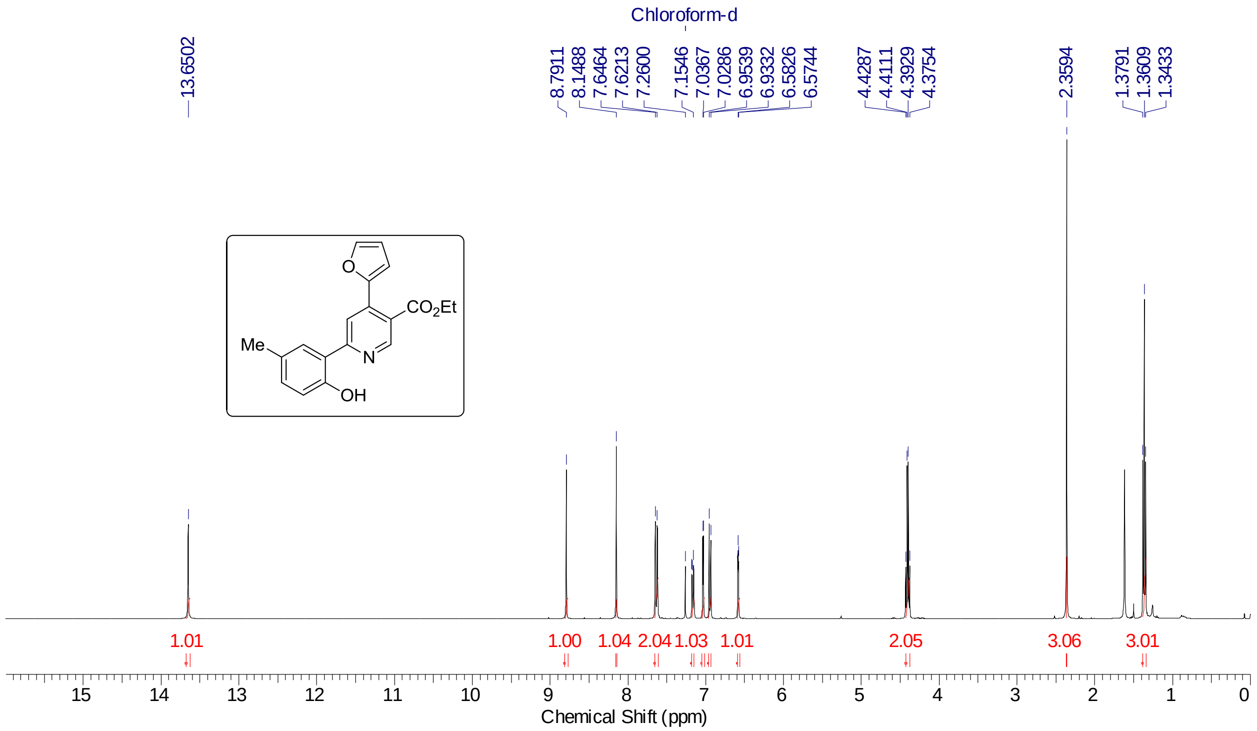
Chloroform-d
13.6502
8.7911
8.1488
7.6464
7.6213
7.2600
7.1546
7.0367
7.0286
6.9539
6.9332
6.5826
6.5744
4.4287
4.4111
4.3929
4.3754
2.3594
1.3791
1.3609
1.3433
CO2Et
Me
N
OH
O
1.01
1.00
1.04
2.04
1.03
1.01
2.05
3.06
3.01
Chemical Shift (ppm)

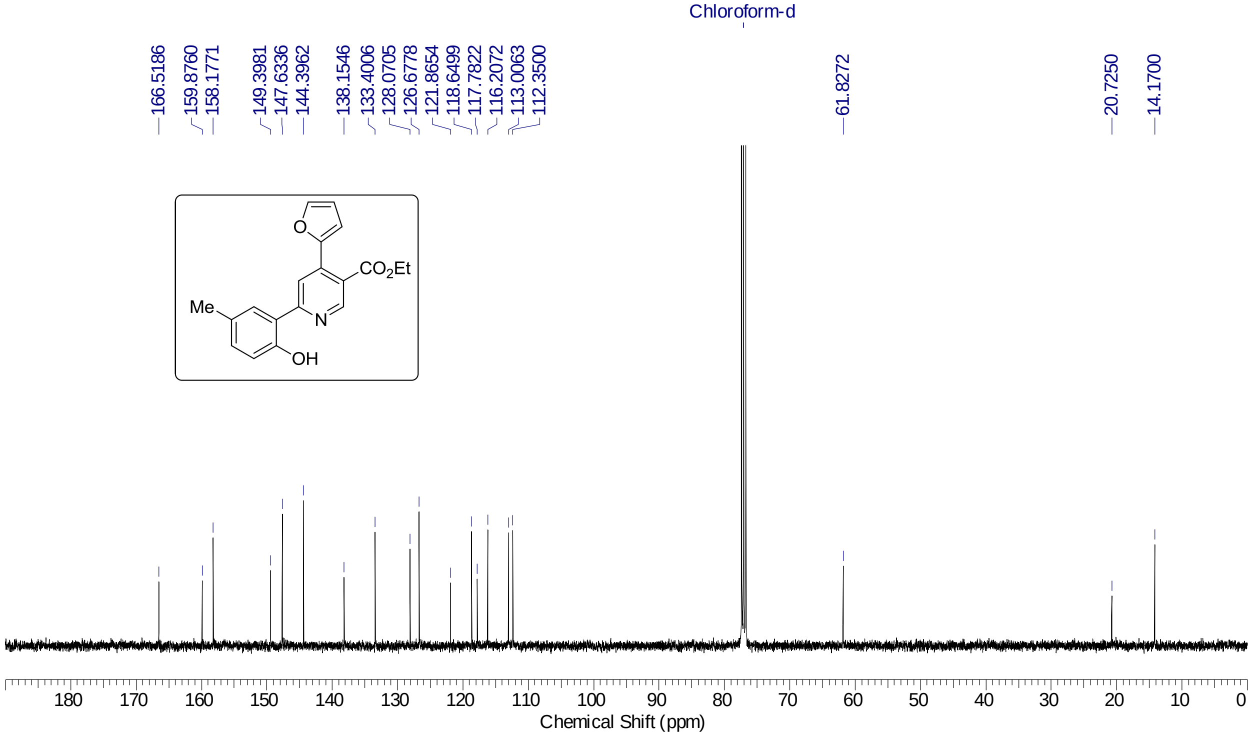
Chloroform-d
166.5186
159.8760
158.1771
149.3981
147.6336
144.3962
138.1546
133.4006
128.0705
126.6778
121.8654
118.6499
117.7822
116.2072
113.0063
112.3500
61.8272
20.7250
14.1700
CO2Et
Me
N
OH
O
Chemical Shift (ppm)

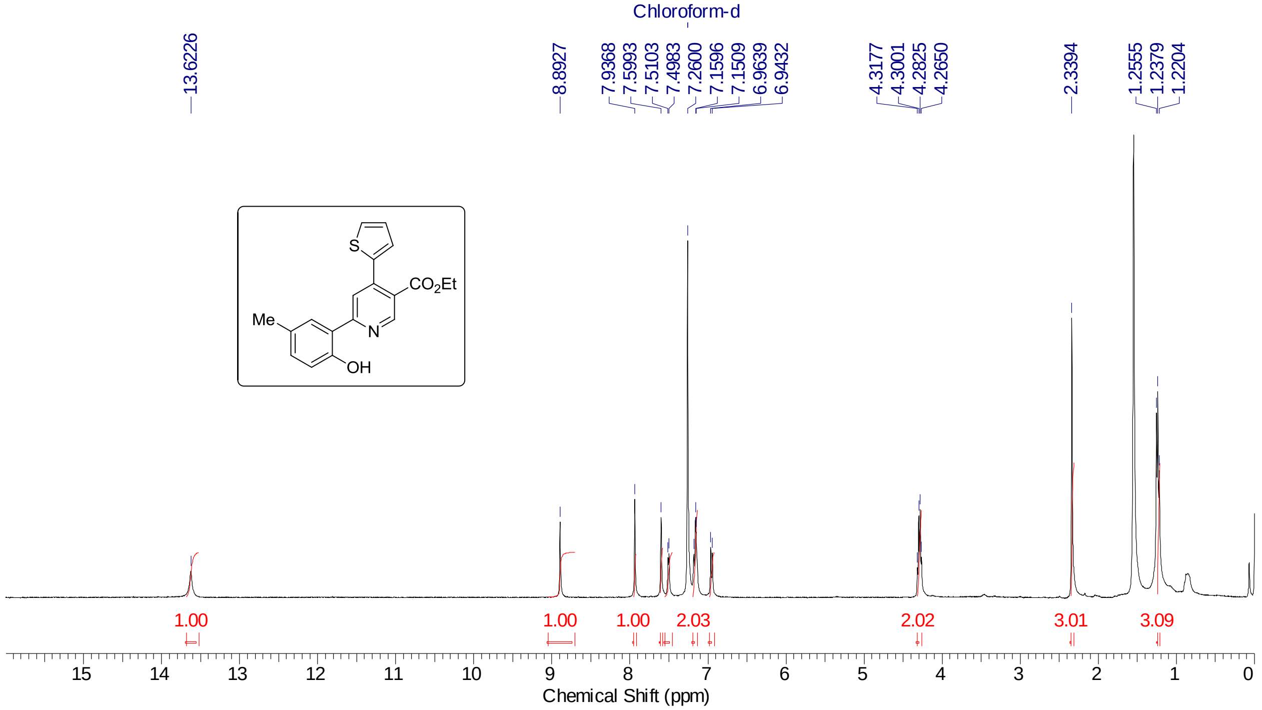
Chloroform-d
13.6226
8.8927
7.9368
7.5993
7.5103
7.4983
7.2600
7.1596
7.1509
6.9639
6.9432
4.3177
4.3001
4.2825
4.2650
2.3394
1.2555
1.2379
1.2204
S
CO2Et
Me
N
OH
1.00
1.00
1.00
2.03
2.02
3.01
3.09
Chemical Shift (ppm)

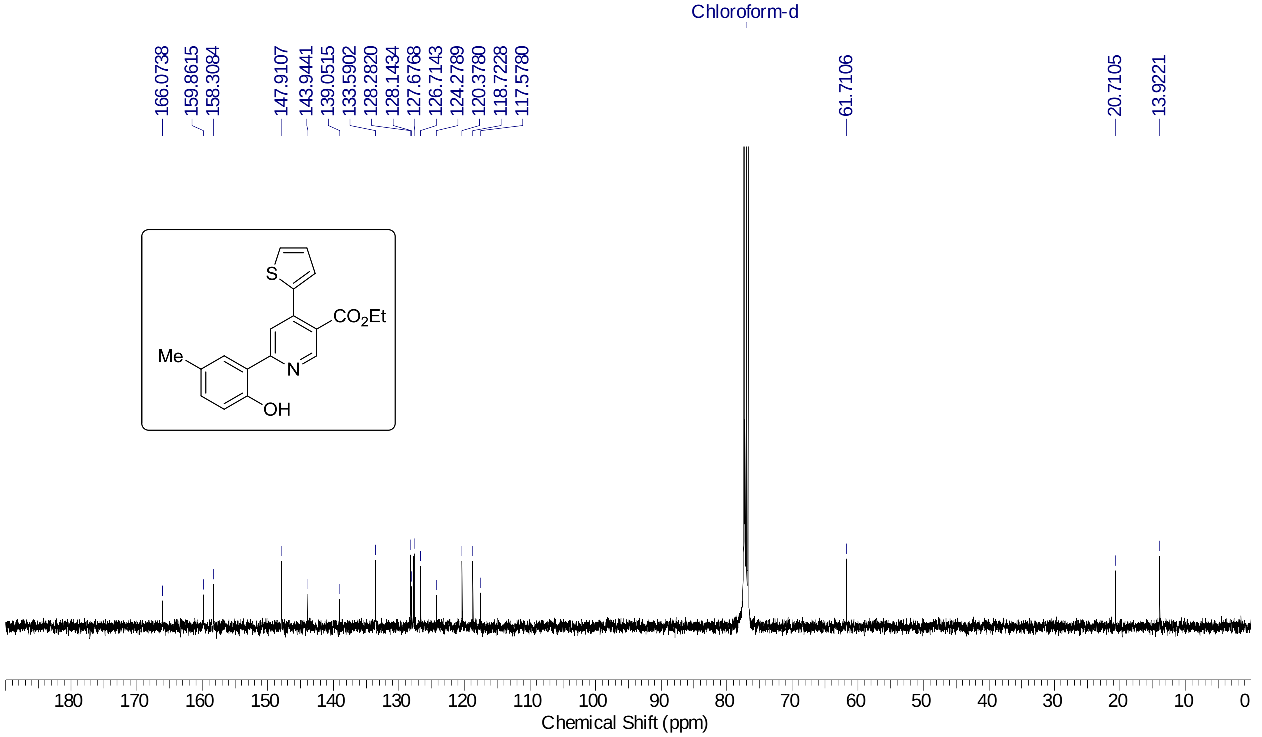
Chloroform-d
166.0738
159.8615
158.3084
147.9107
143.9441
139.0515
133.5902
128.2820
128.1434
127.6768
126.7143
124.2789
120.3780
118.7228
117.5780
61.7106
20.7105
13.9221
S
CO2Et
Me
N
OH
Chemical Shift (ppm)

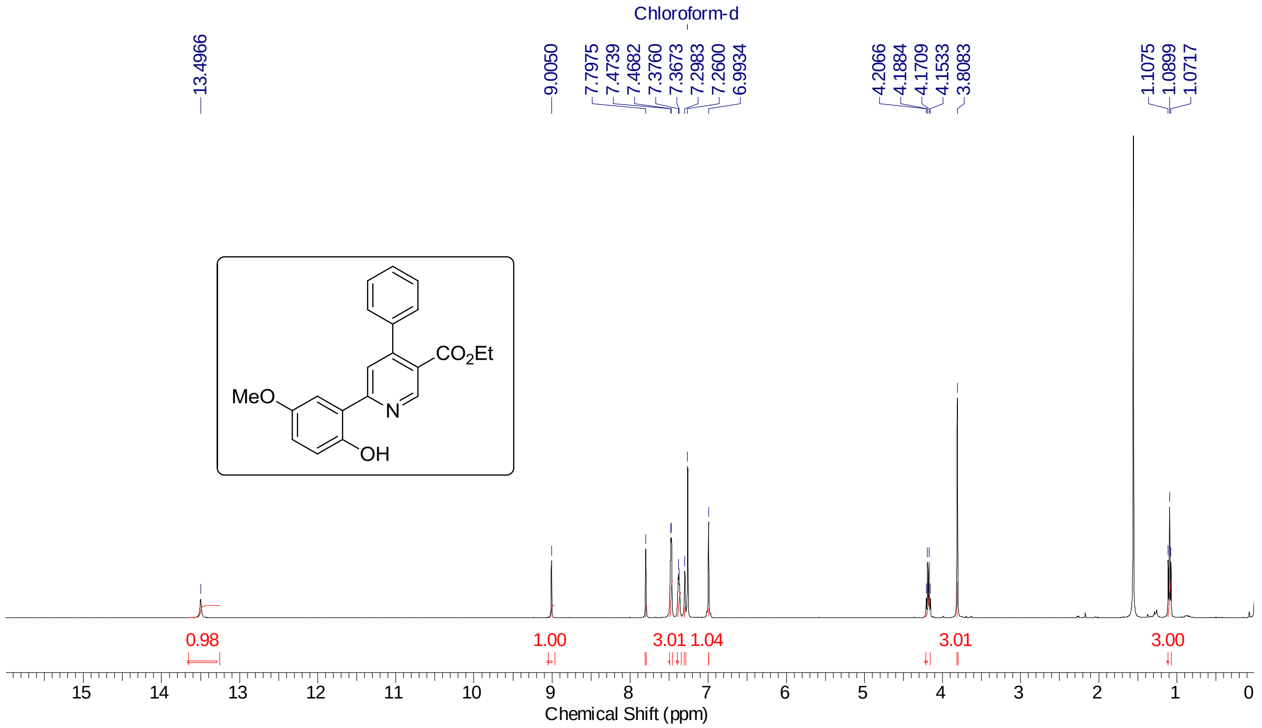
Chloroform-d
13.4966
9.0050
7.7975
7.4739
7.4682
7.3760
7.3673
7.2983
7.2600
6.9934
4.2066
4.1884
4.1709
4.1533
3.8083
1.1075
1.0899
1.0717
MeO
CO2Et
N
OH
0.98
1.00
3.01
1.04
3.01
3.00
Chemical Shift (ppm)

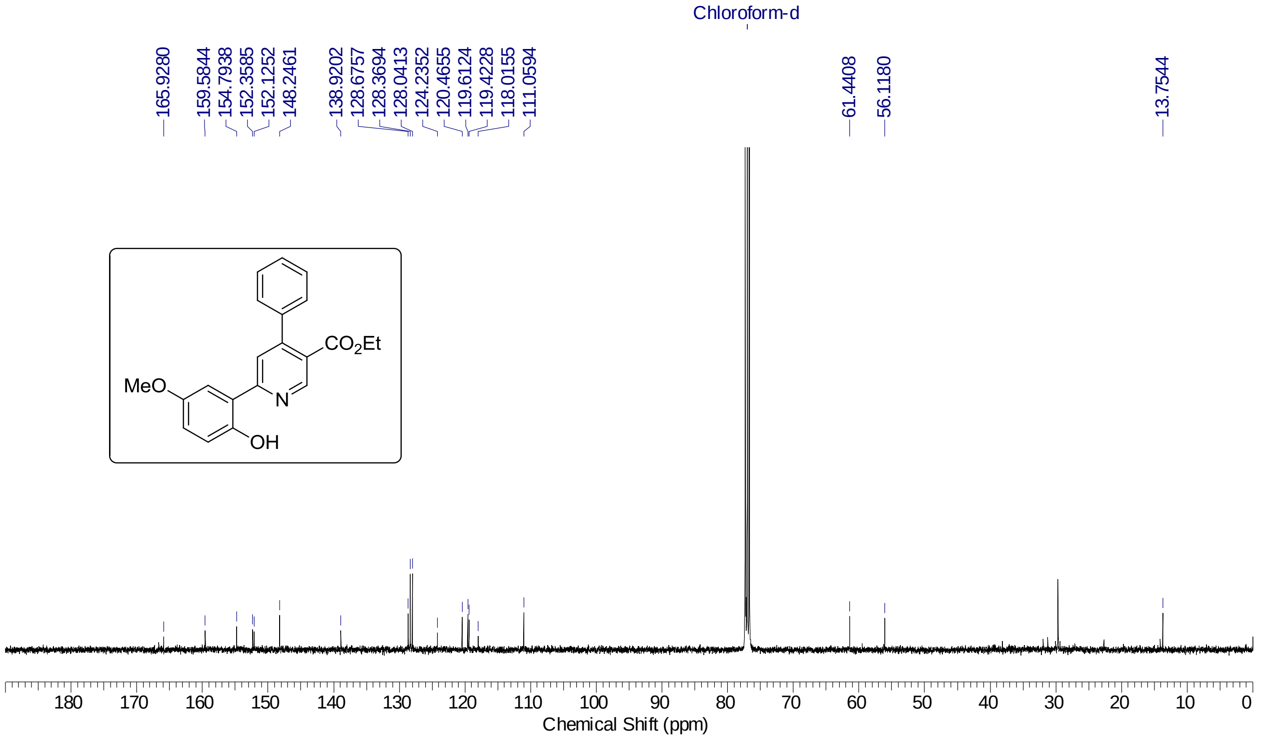
Chloroform-d
165.9280
159.5844
154.7938
152.3585
152.1252
148.2461
138.9202
128.6757
128.3694
128.0413
124.2352
120.4655
119.6124
119.4228
118.0155
111.0594
61.4408
56.1180
13.7544
MeO
CO2Et
N
OH
Chemical Shift (ppm)

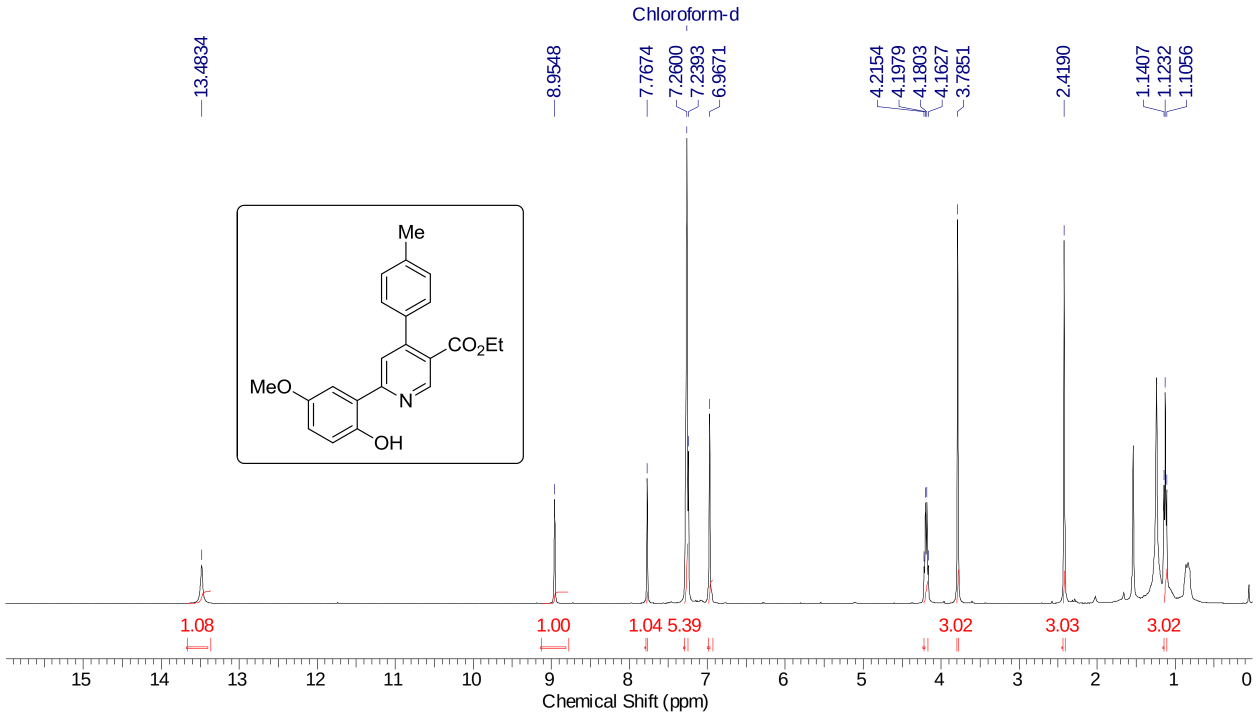
Chloroform-d
13.4834
8.9548
7.7674
7.2600
7.2393
6.9671
4.2154
4.1979
4.1803
4.1627
3.7851
2.4190
1.1407
1.1232
1.1056
Me
CO2Et
MeO
N
OH
1.08
1.00
1.04
5.39
3.02
3.03
3.02
Chemical Shift (ppm)

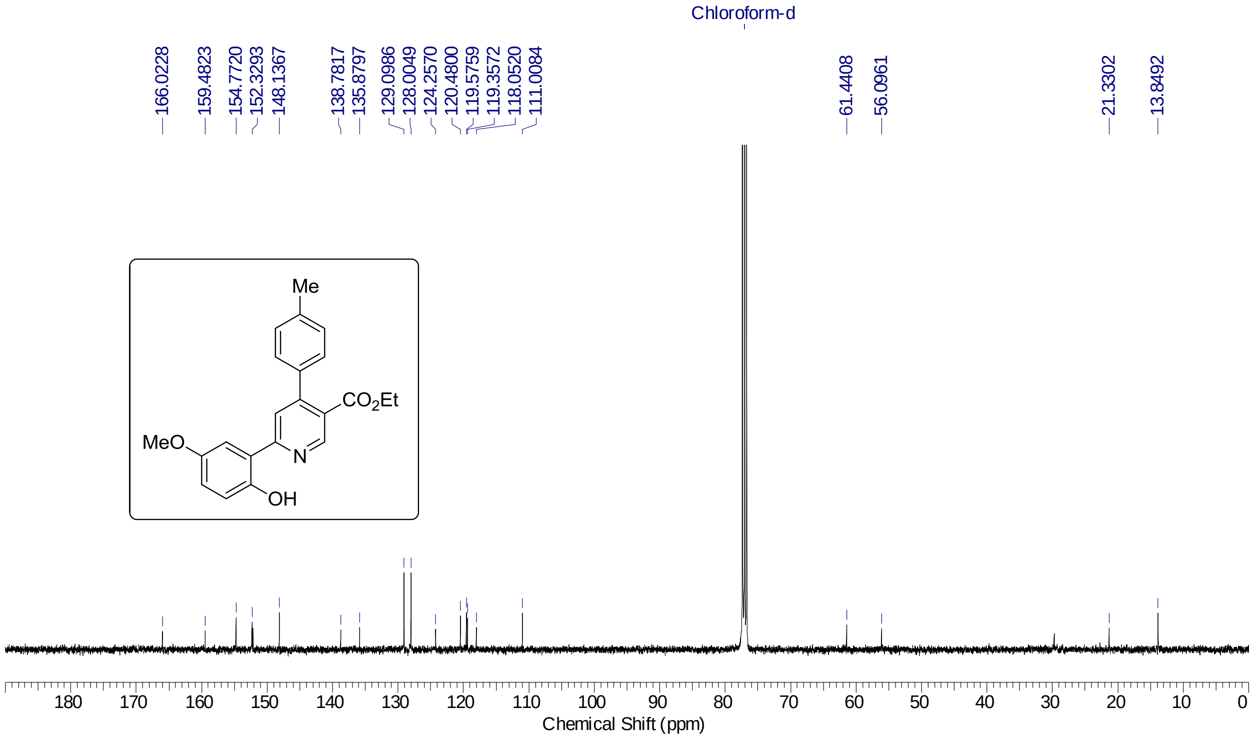
Chloroform-d
166.0228
159.4823
154.7720
152.3293
148.1367
138.7817
135.8797
129.0986
128.0049
124.2570
120.4800
119.5759
119.3572
118.0520
111.0084
61.4408
56.0961
21.3302
13.8492
Me
CO2Et
MeO
N
OH
Chemical Shift (ppm)

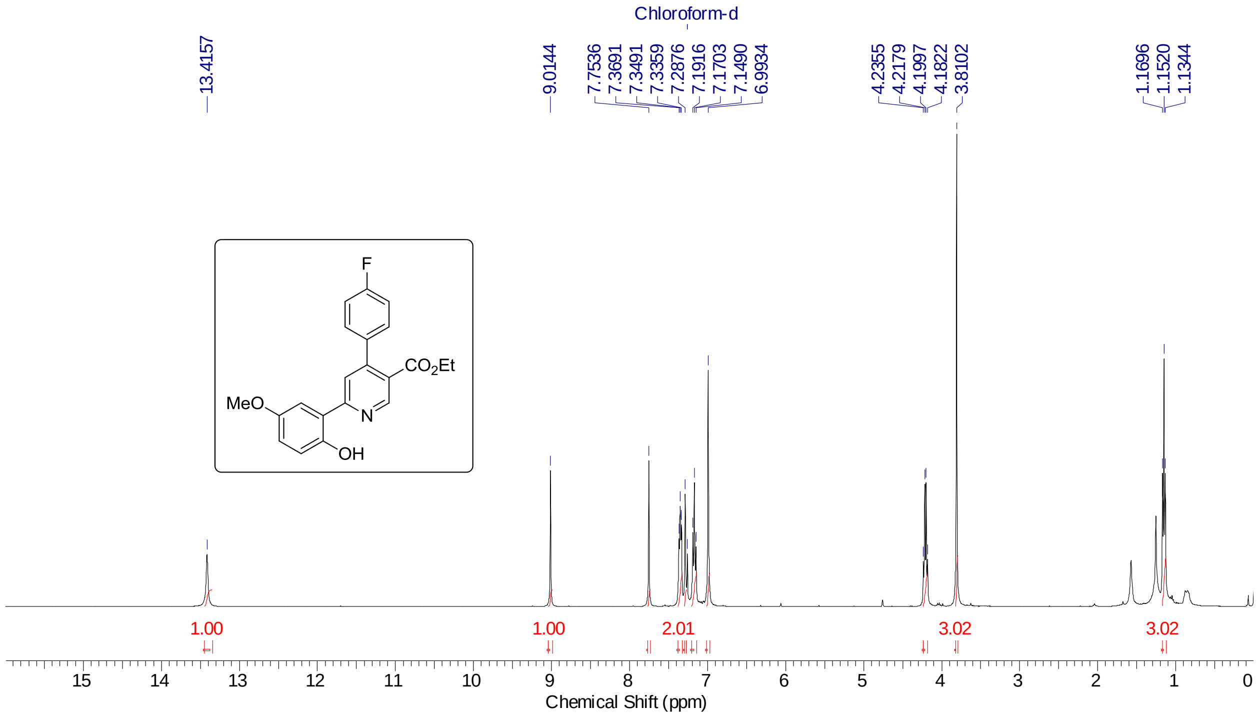

Chloroform-d
13.4157
9.0144
7.7536
7.3691
7.3491
7.3359
7.2876
7.1916
7.1703
7.1490
6.9934
4.2355
4.2179
4.1997
4.1822
3.8102
1.1696
1.1520
1.1344
F
CO2Et
MeO
N
OH
1.00
1.00
2.01
3.02
3.02
15 14 13 12 11 10 9 8 7 6 5 4 3 2 1 0
Chemical Shift (ppm)

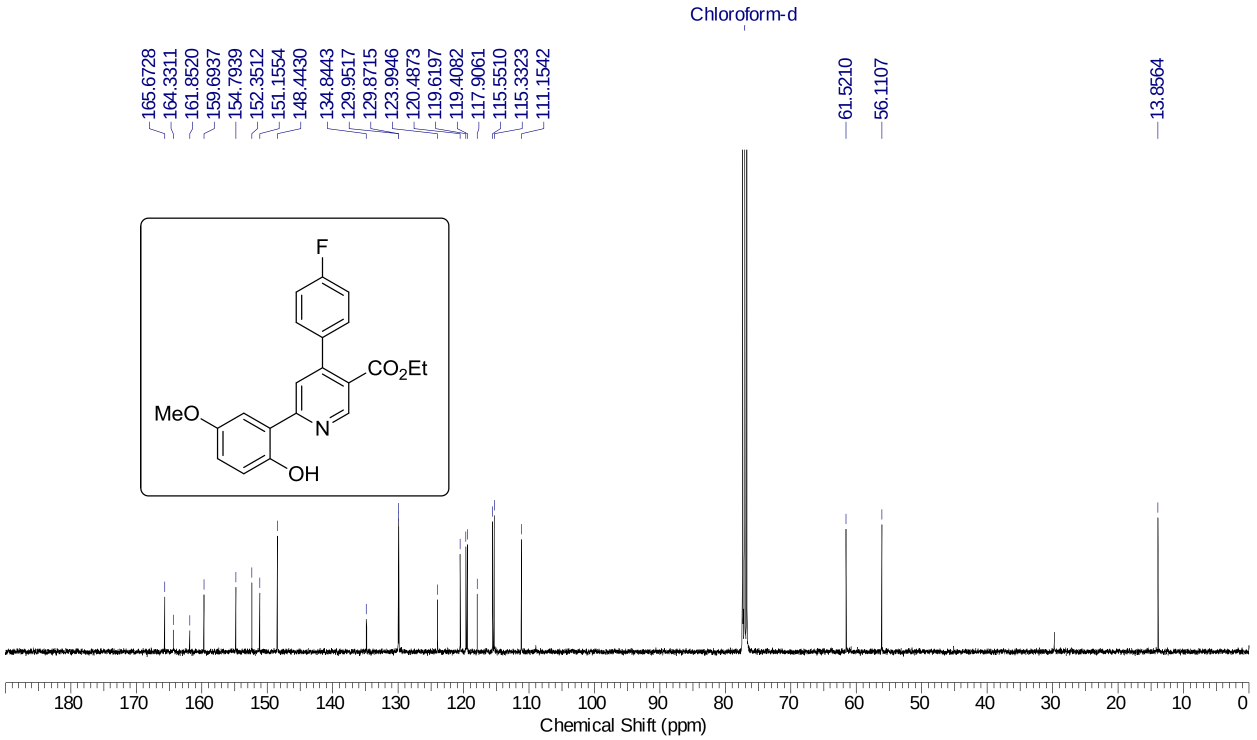

Chloroform-d
165.6728
164.3311
161.8520
159.6937
154.7939
152.3512
151.1554
148.4430
134.8443
129.9517
129.8715
123.9946
120.4873
119.6197
119.4082
117.9061
115.5510
115.3323
111.1542
61.5210
56.1107
13.8564
F
CO2Et
MeO
N
OH
180 170 160 150 140 130 120 110 100 90 80 70 60 50 40 30 20 10 0
Chemical Shift (ppm)

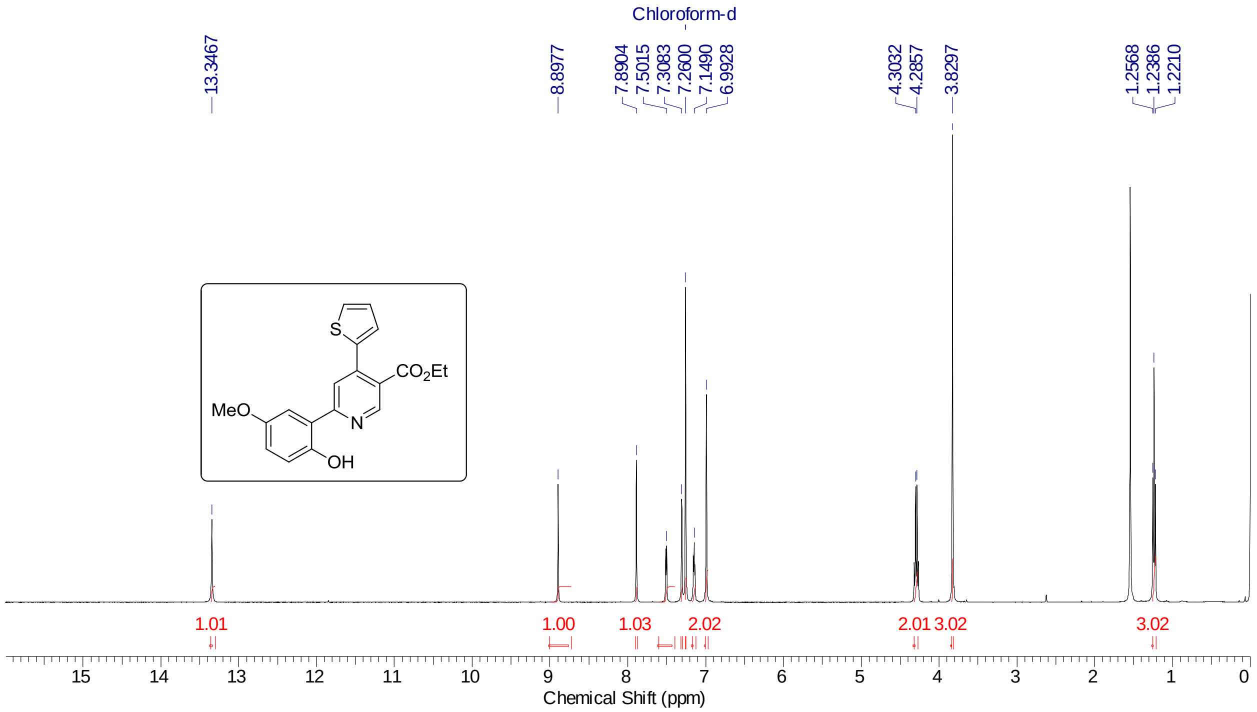
Chloroform-d
13.3467
8.8977
7.8904
7.5015
7.3083
7.2600
7.1490
6.9928
4.3032
4.2857
3.8297
1.2568
1.2386
1.2210
MeO
OH
N
S
CO2Et
1.01
1.00
1.03
2.02
2.01
3.02
3.02
Chemical Shift (ppm)

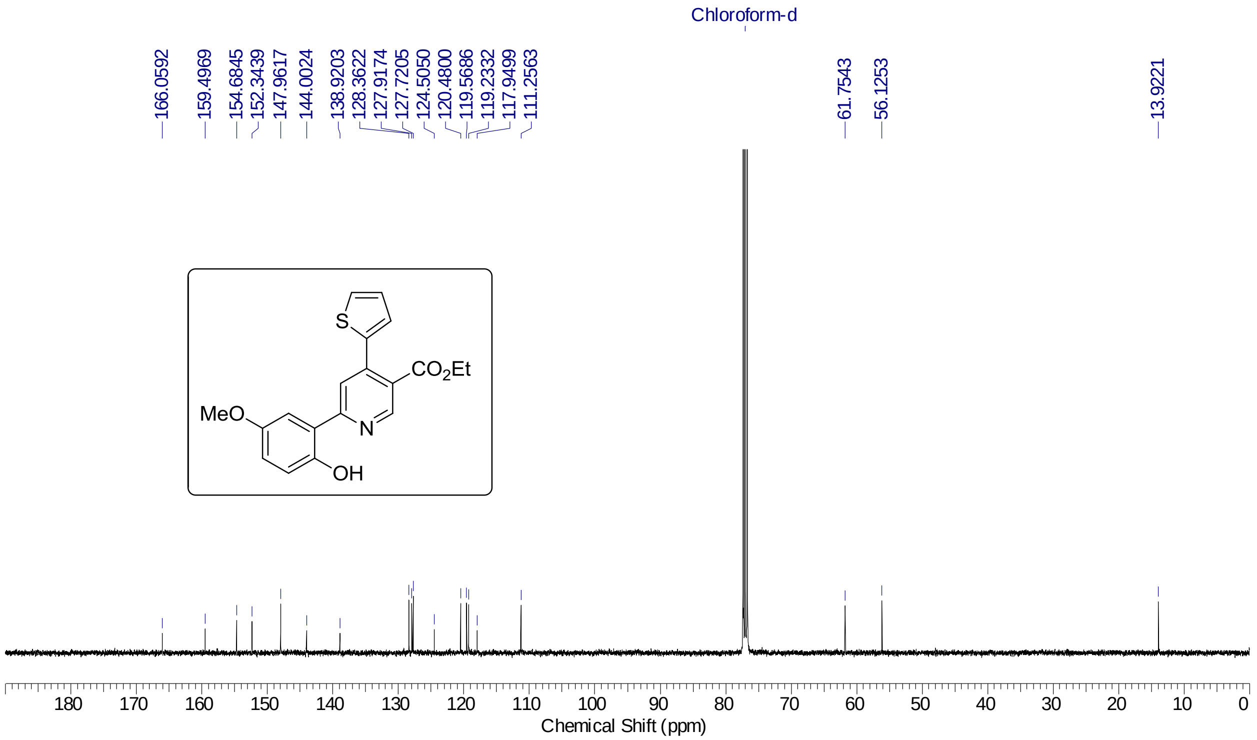
Chloroform-d
166.0592
159.4969
154.6845
152.3439
147.9617
144.0024
138.9203
128.3622
127.9174
127.7205
124.5050
120.4800
119.5686
119.2332
117.9499
111.2563
61.7543
56.1253
13.9221
MeO
OH
N
S
CO2Et
Chemical Shift (ppm)

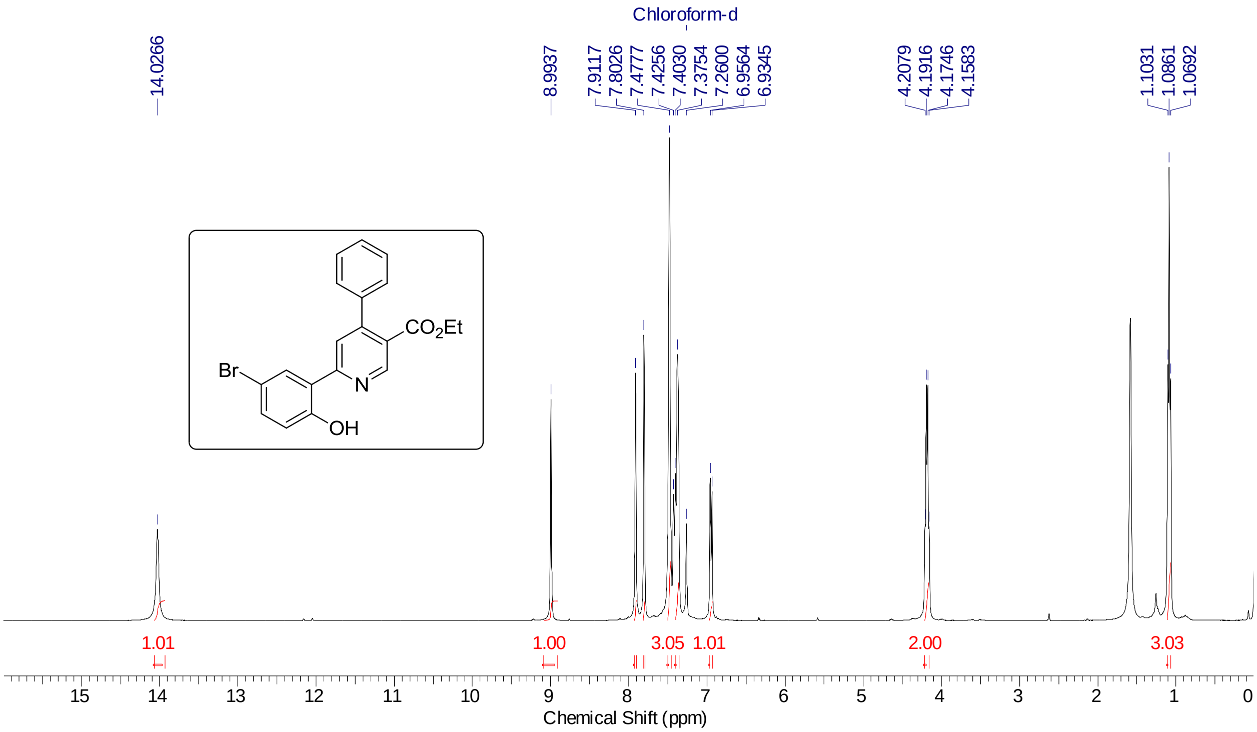
Chloroform-d
14.0266
8.9937
7.9117
7.8026
7.4777
7.4256
7.4030
7.3754
7.2600
6.9564
6.9345
4.2079
4.1916
4.1746
4.1583
1.1031
1.0861
1.0692
Br
N
OH
CO2Et
1.01
1.00
3.05
1.01
2.00
3.03
15
14
13
12
11
10
9
8
7
6
5
4
3
2
1
0
Chemical Shift (ppm)

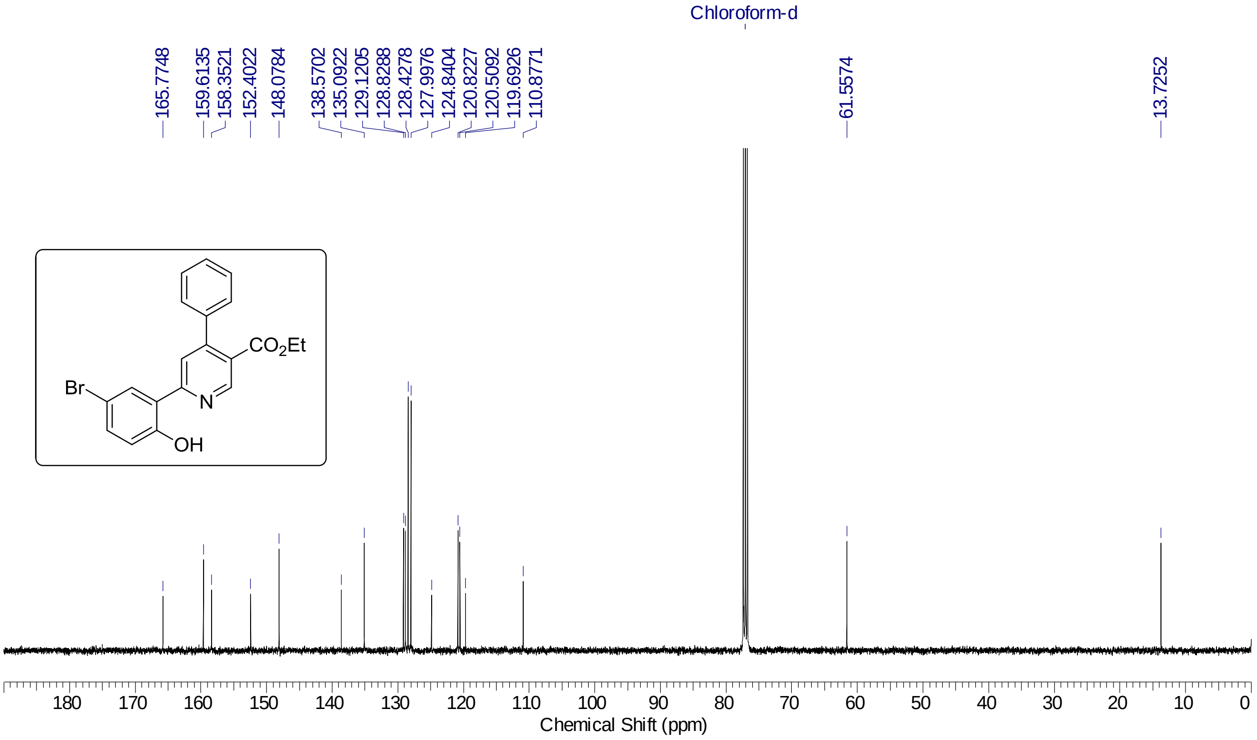
Chloroform-d
165.7748
159.6135
158.3521
152.4022
148.0784
138.5702
135.0922
129.1205
128.8288
128.4278
127.9976
124.8404
120.8227
120.5092
119.6926
110.8771
61.5574
13.7252
Br
N
OH
CO2Et
180
170
160
150
140
130
120
110
100
90
80
70
60
50
40
30
20
10
0
Chemical Shift (ppm)

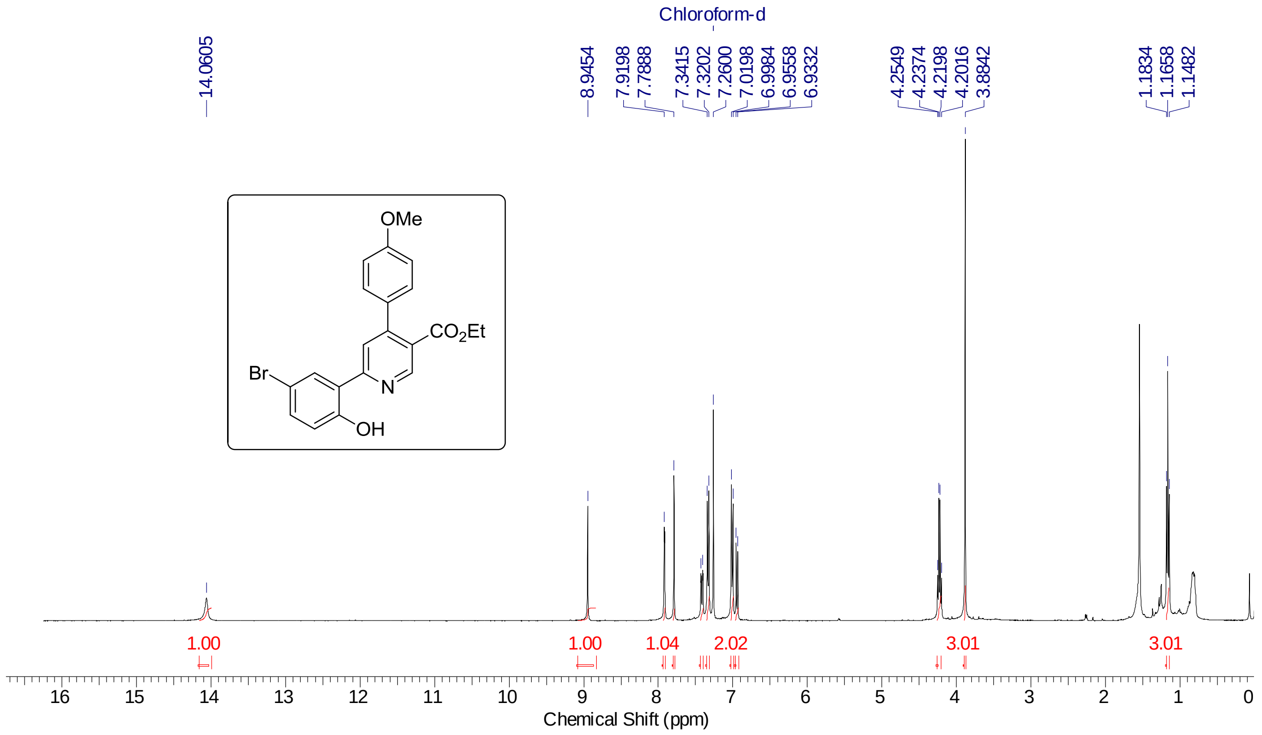
Chloroform-d
14.0605
8.9454
7.9198
7.7888
7.3415
7.3202
7.2600
7.0198
6.9984
6.9558
6.9332
4.2549
4.2374
4.2198
4.2016
3.8842
1.1834
1.1658
1.1482
OMe
CO_2Et
Br
N
OH
1.00
1.00
1.04
2.02
3.01
3.01
16
15
14
13
12
11
10
9
8
7
6
5
4
3
2
1
0
Chemical Shift (ppm)

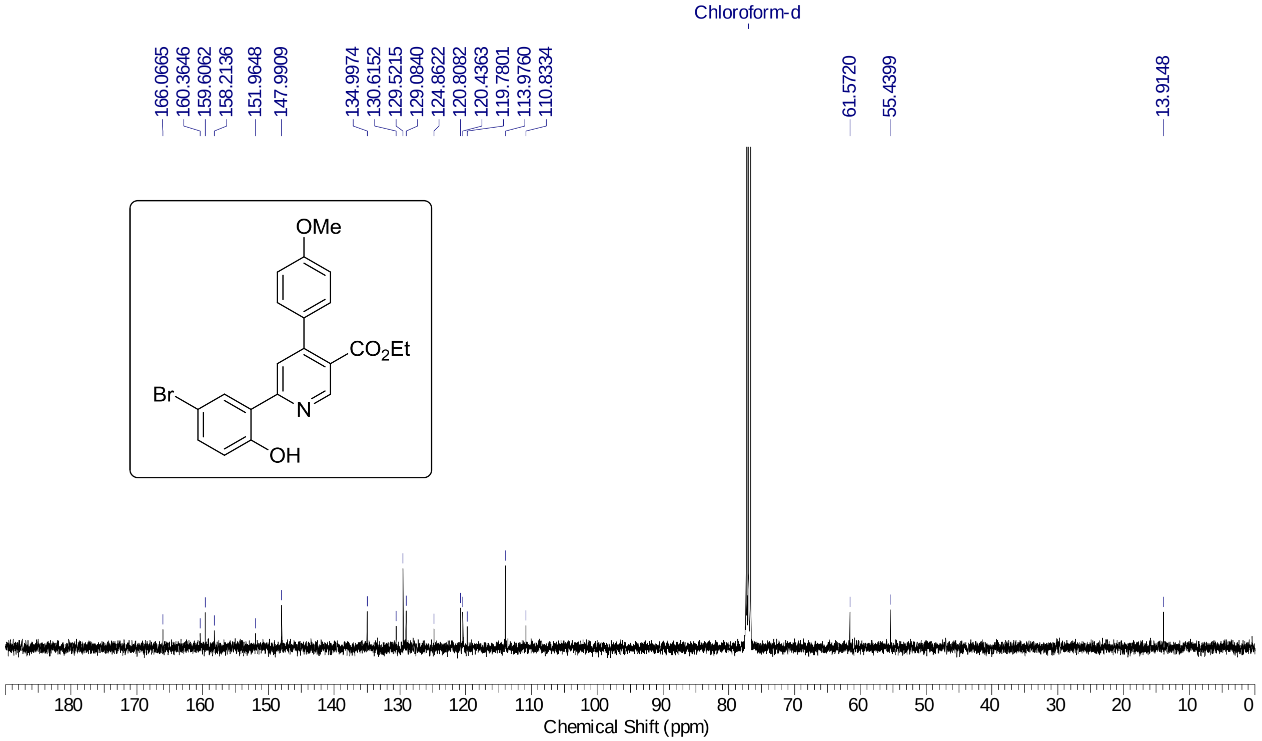
Chloroform-d
166.0665
160.3646
159.6062
158.2136
151.9648
147.9909
134.9974
130.6152
129.5215
129.0840
124.8622
120.8082
120.4363
119.7801
113.9760
110.8334
61.5720
55.4399
13.9148
OMe
CO_2Et
Br
N
OH
180
170
160
150
140
130
120
110
100
90
80
70
60
50
40
30
20
10
0
Chemical Shift (ppm)

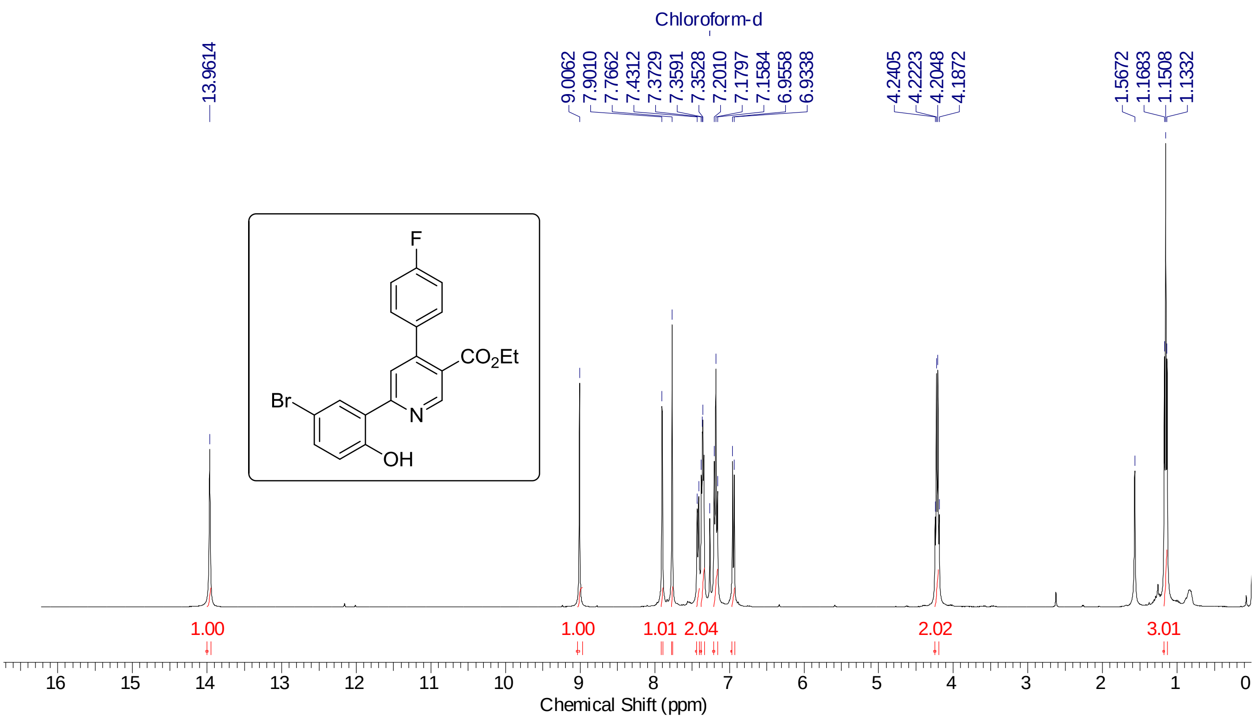
Chloroform-d
13.9614
9.0062
7.9010
7.7662
7.4312
7.3729
7.3591
7.3528
7.2010
7.1797
7.1584
6.9558
6.9338
4.2405
4.2223
4.2048
4.1872
1.5672
1.1683
1.1508
1.1332
F
CO2Et
Br
N
OH
1.00
1.00
1.01
2.04
2.02
3.01
Chemical Shift (ppm)

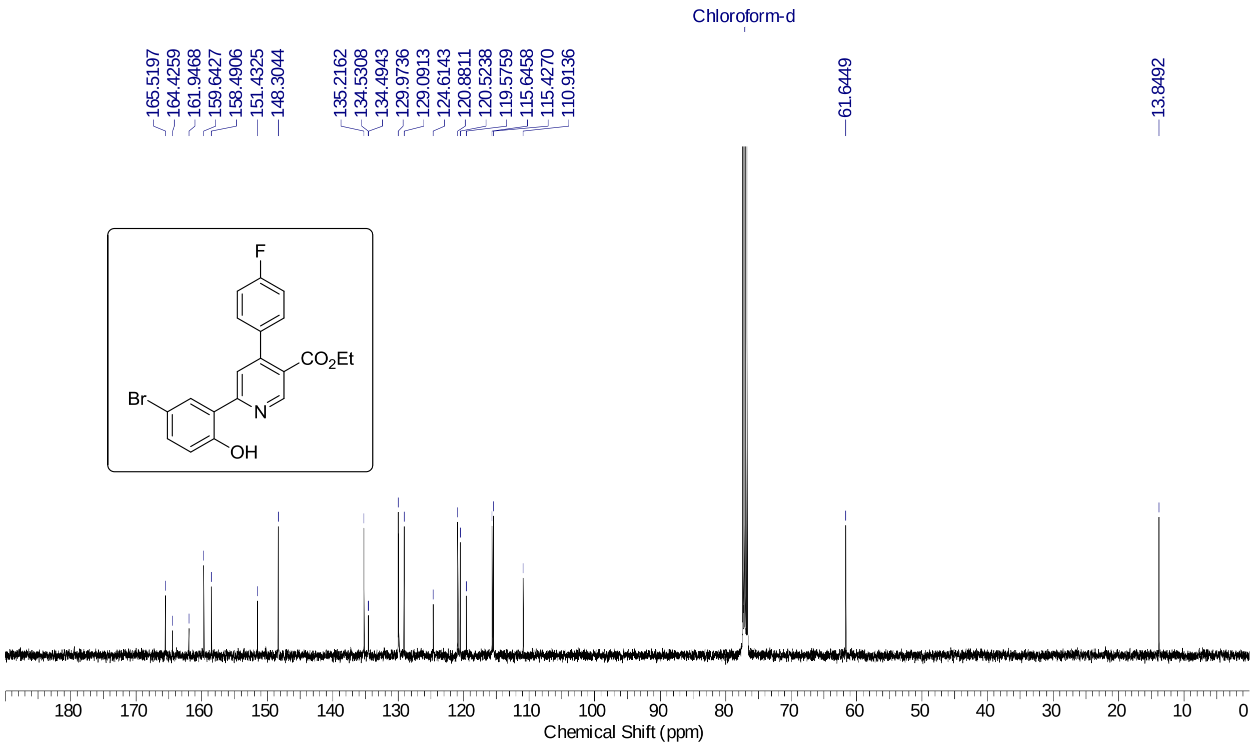
Chloroform-d
165.5197
164.4259
161.9468
159.6427
158.4906
151.4325
148.3044
135.2162
134.5308
134.4943
129.9736
129.0913
124.6143
120.8811
120.5238
119.5759
115.6458
115.4270
110.9136
61.6449
13.8492
F
CO2Et
Br
N
OH
Chemical Shift (ppm)

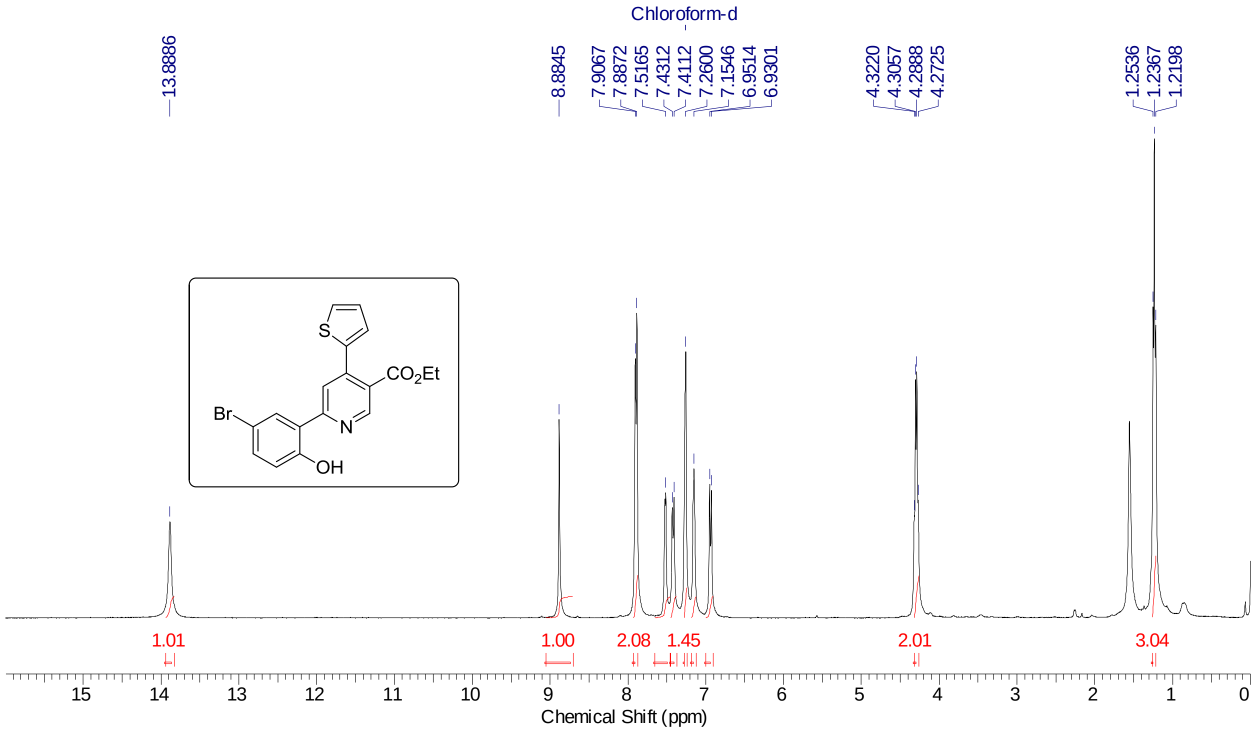
Chloroform-d
13.8886
8.8845
7.9067
7.8872
7.5165
7.4312
7.4112
7.2600
7.1546
6.9514
6.9301
4.3220
4.3057
4.2888
4.2725
1.2536
1.2367
1.2198
CO2Et
Br
N
OH
S
1.01
1.00
2.08
1.45
2.01
3.04
Chemical Shift (ppm)

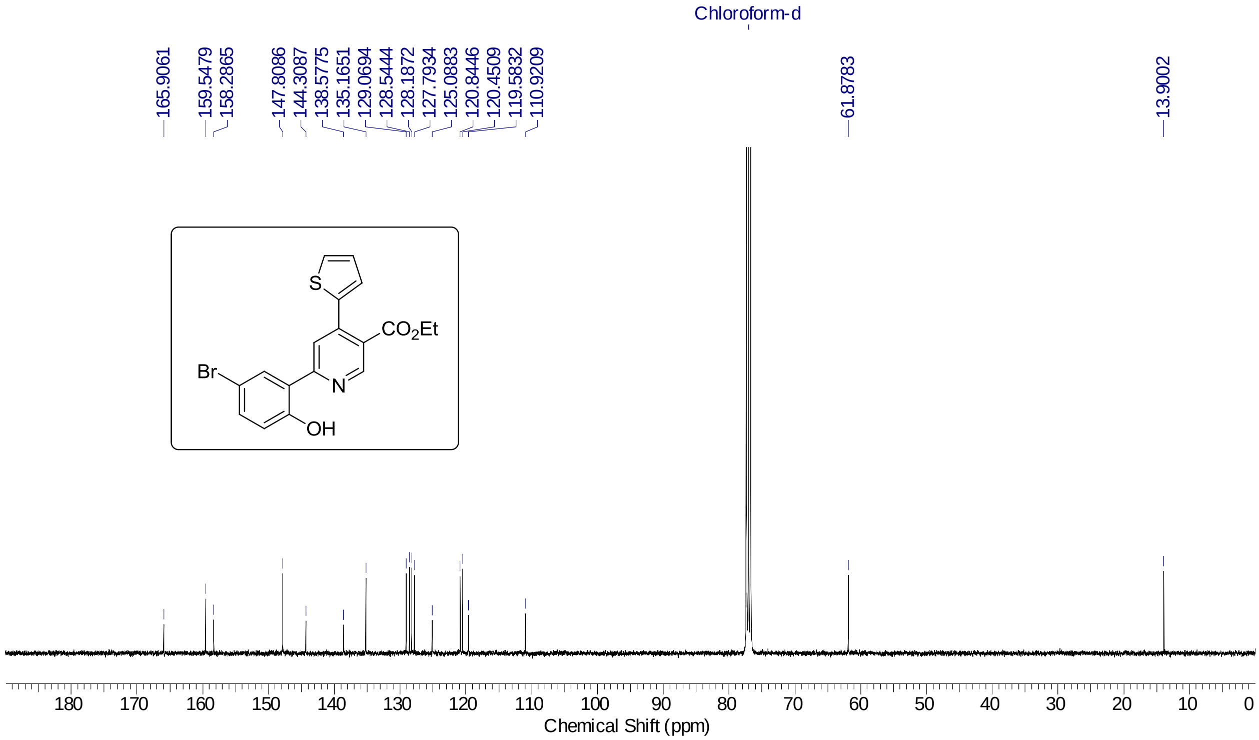
Chloroform-d
165.9061
159.5479
158.2865
147.8086
144.3087
138.5775
135.1651
129.0694
128.5444
128.1872
127.7934
125.0883
120.8446
120.4509
119.5832
110.9209
61.8783
13.9002
CO2Et
Br
N
OH
S
Chemical Shift (ppm)

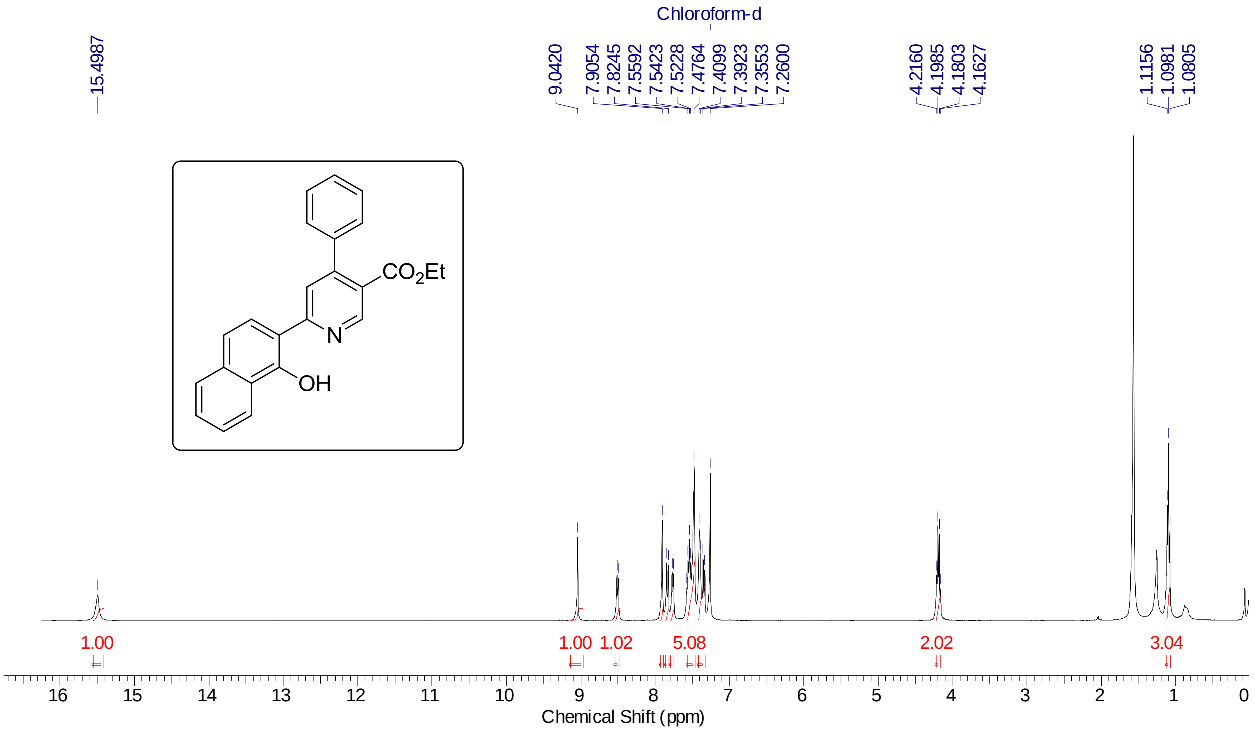
Chloroform-d
15.4987
9.0420
7.9054
7.8245
7.5592
7.5423
7.5228
7.4764
7.4099
7.3923
7.3553
7.2600
4.2160
4.1985
4.1803
4.1627
1.1156
1.0981
1.0805
CO2Et
N
OH
1.00
1.00
1.02
5.08
2.02
3.04
Chemical Shift (ppm)

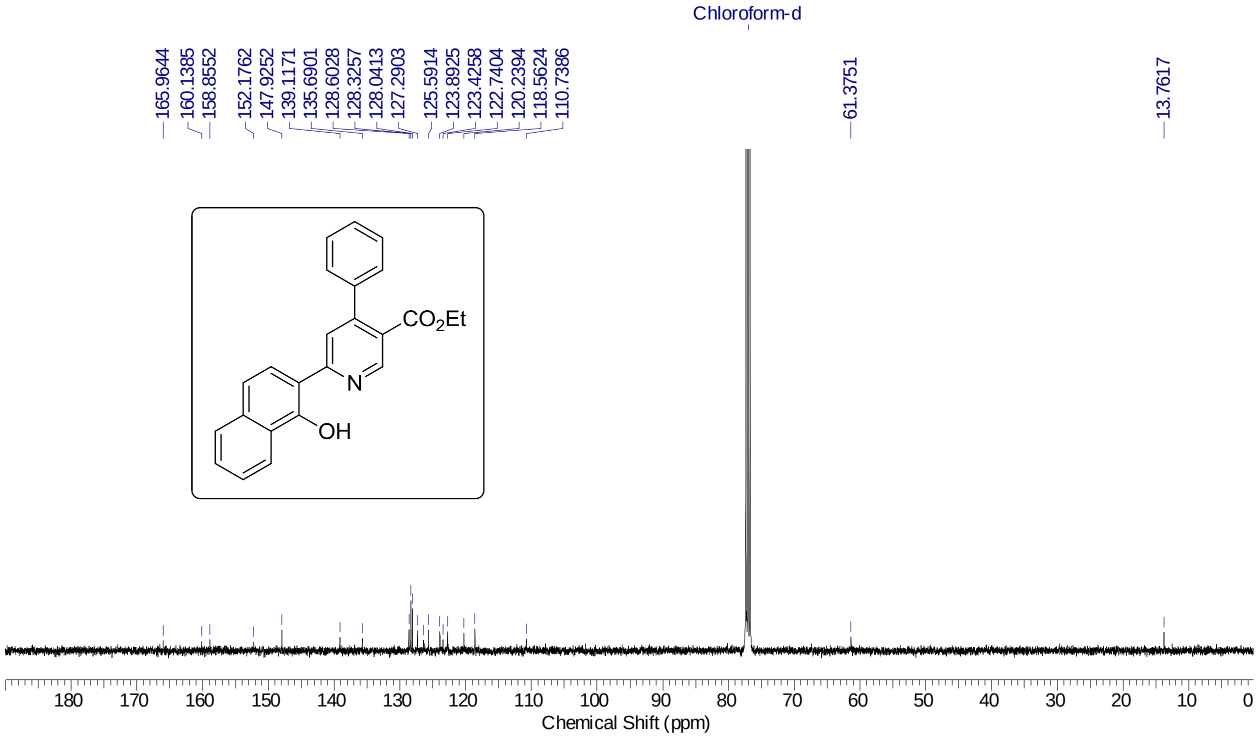
Chloroform-d
165.9644
160.1385
158.8552
152.1762
147.9252
139.1171
135.6901
128.6028
128.3257
128.0413
127.2903
125.5914
123.8925
123.4258
122.7404
120.2394
118.5624
110.7386
61.3751
13.7617
CO2Et
N
OH
Chemical Shift (ppm)

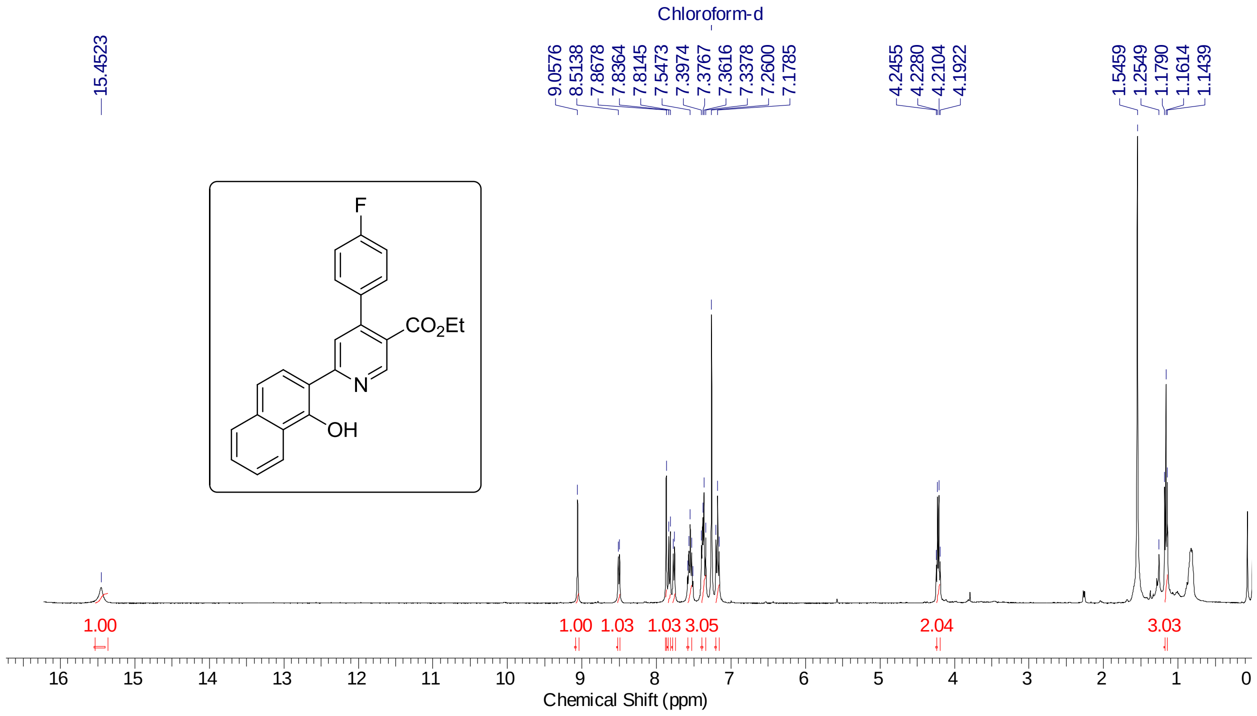

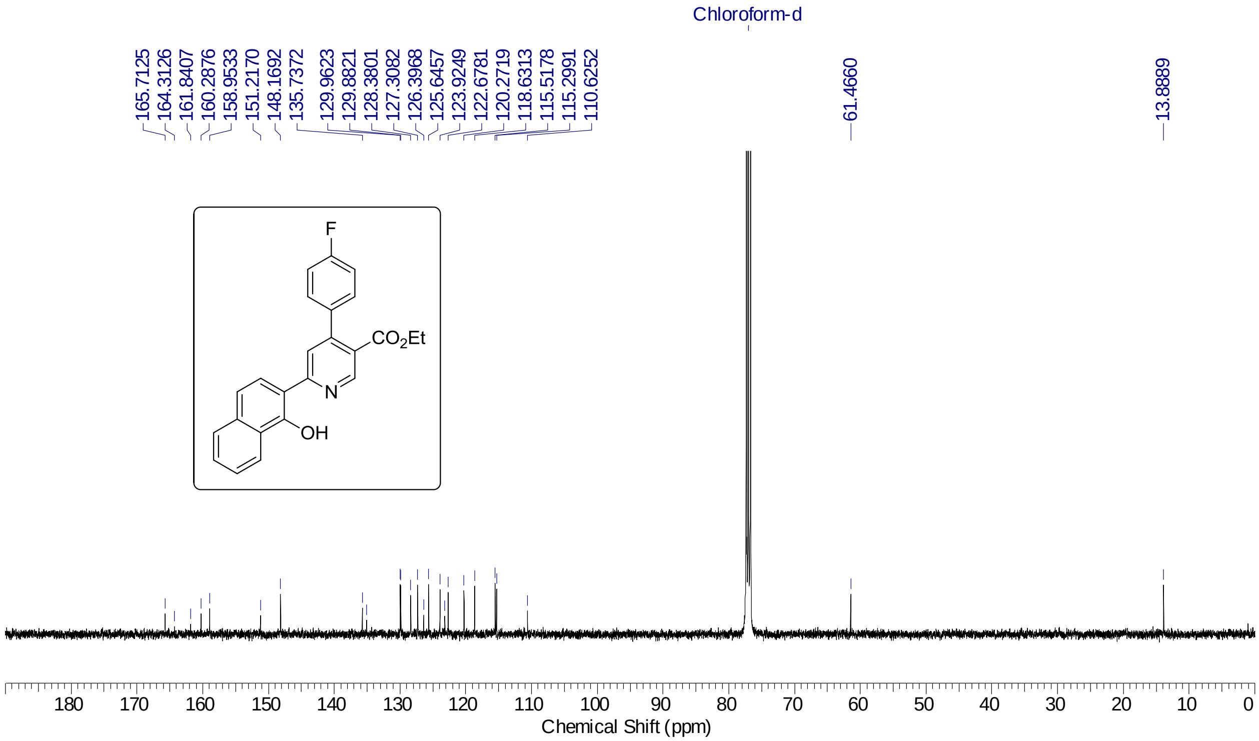

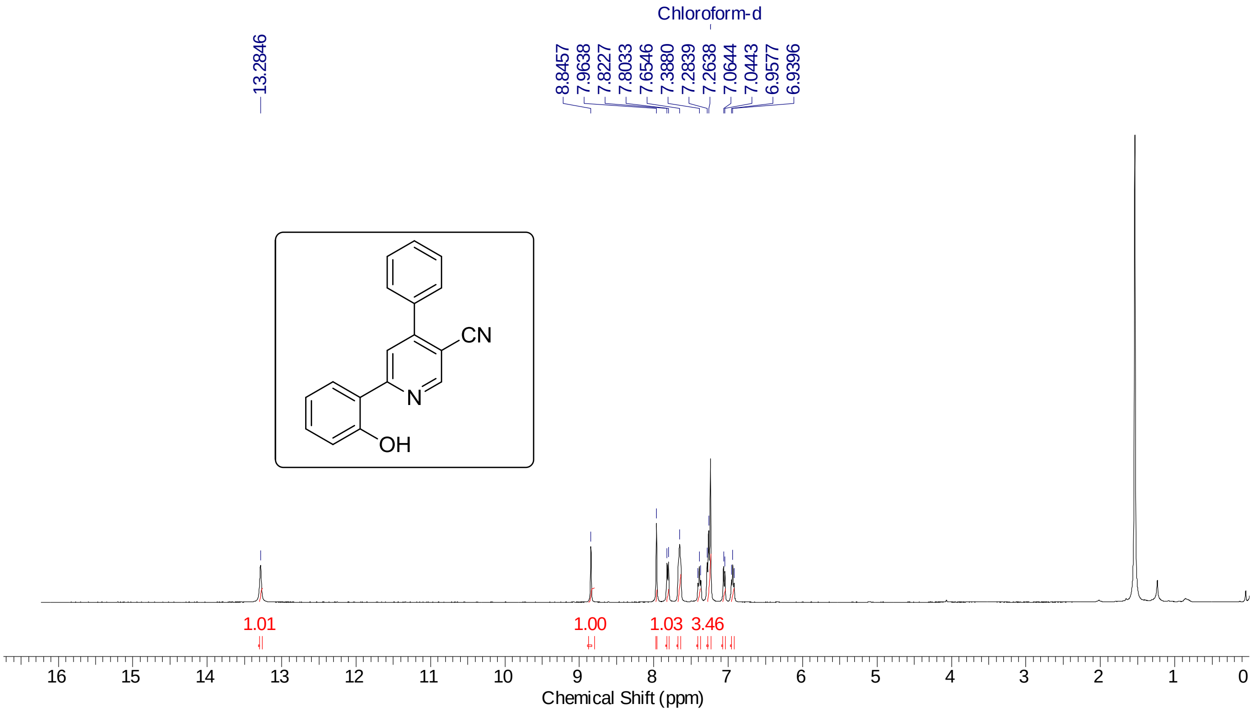
13.2846
8.8457
7.9638
7.8227
7.8033
7.6546
7.3880
7.2839
7.2638
7.0644
7.0443
6.9577
6.9396
Chloroform-d
CN
N
OH
1.01
1.00
1.03
3.46
Chemical Shift (ppm)

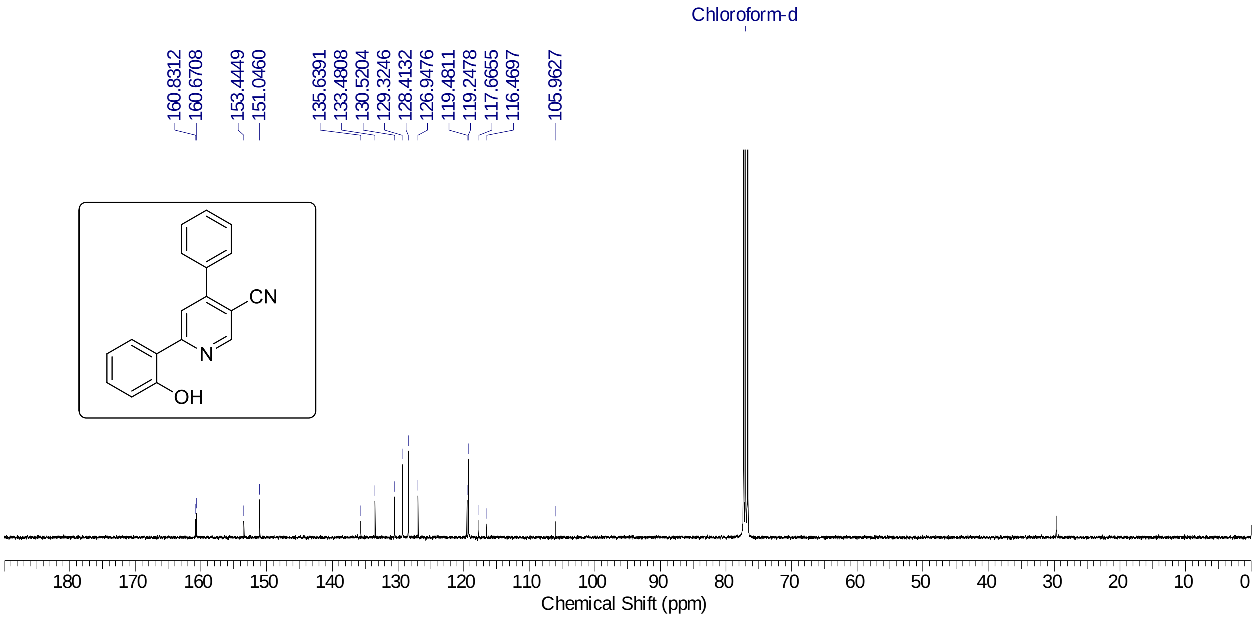
160.8312
160.6708
153.4449
151.0460
135.6391
133.4808
130.5204
129.3246
128.4132
126.9476
119.4811
119.2478
117.6655
116.4697
105.9627
Chloroform-d
CN
N
OH
Chemical Shift (ppm)

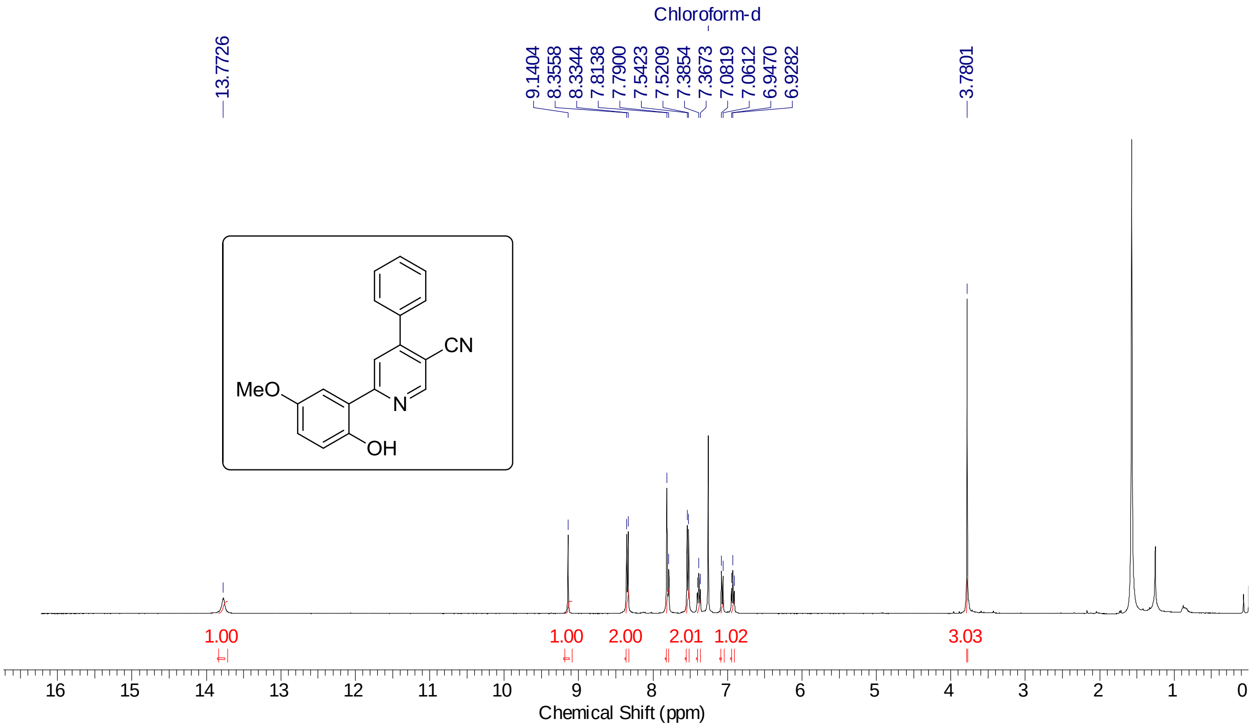
Chloroform-d
13.7726
9.1404
8.3558
8.3344
7.8138
7.7900
7.5423
7.5209
7.3854
7.3673
7.0819
7.0612
6.9470
6.9282
3.7801
MeO
CN
N
OH
1.00
1.00
2.00
2.01
1.02
3.03
Chemical Shift (ppm)

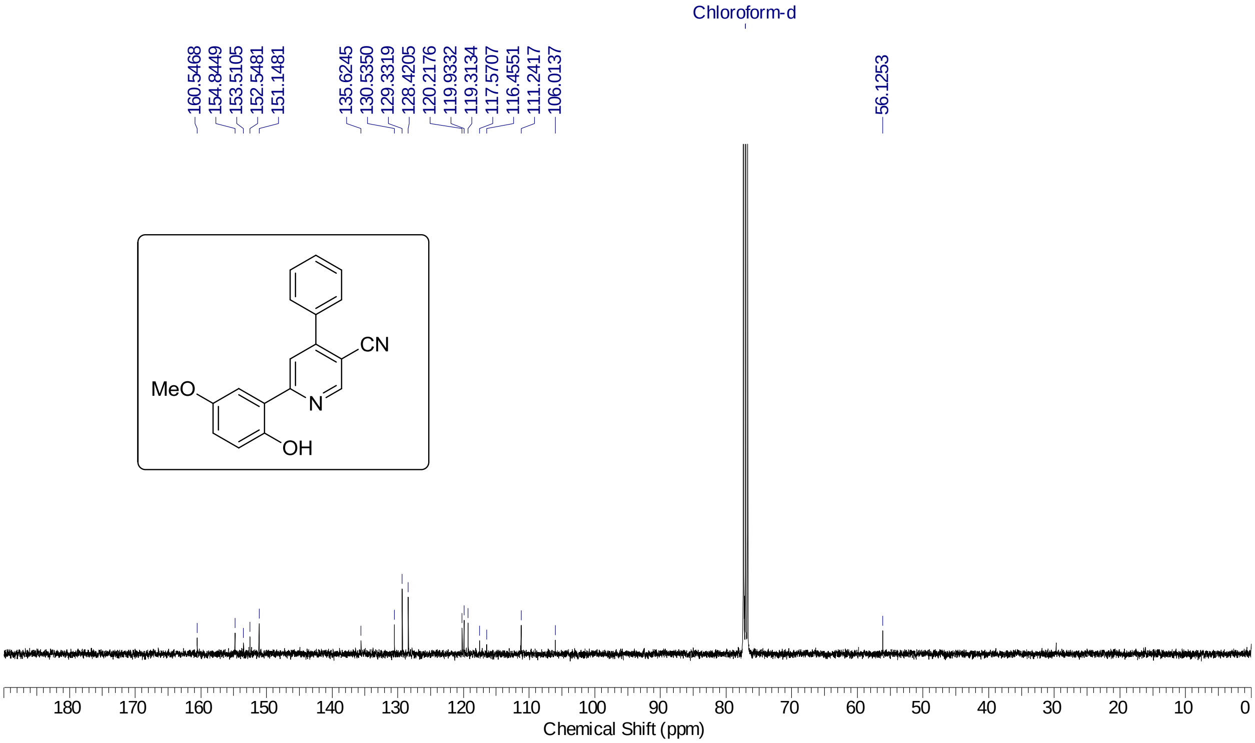
Chloroform-d
160.5468
154.8449
153.5105
152.5481
151.1481
135.6245
130.5350
129.3319
128.4205
120.2176
119.9332
119.3134
117.5707
116.4551
111.2417
106.0137
56.1253
MeO
CN
N
OH
Chemical Shift (ppm)

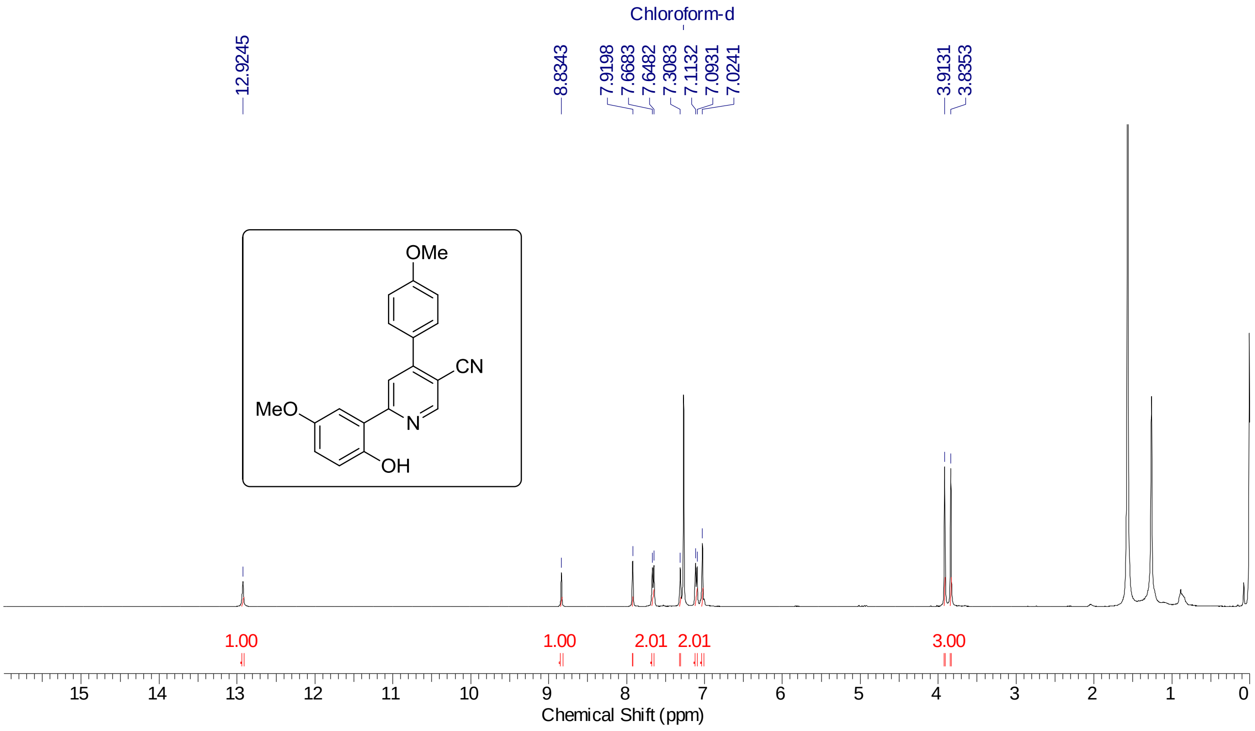
Chloroform-d
12.9245
8.8343
7.9198
7.6683
7.6482
7.3083
7.1132
7.0931
7.0241
3.9131
3.8353
OMe
CN
MeO
N
OH
1.00
1.00
2.01
2.01
3.00
15 14 13 12 11 10 9 8 7 6 5 4 3 2 1 0
Chemical Shift (ppm)

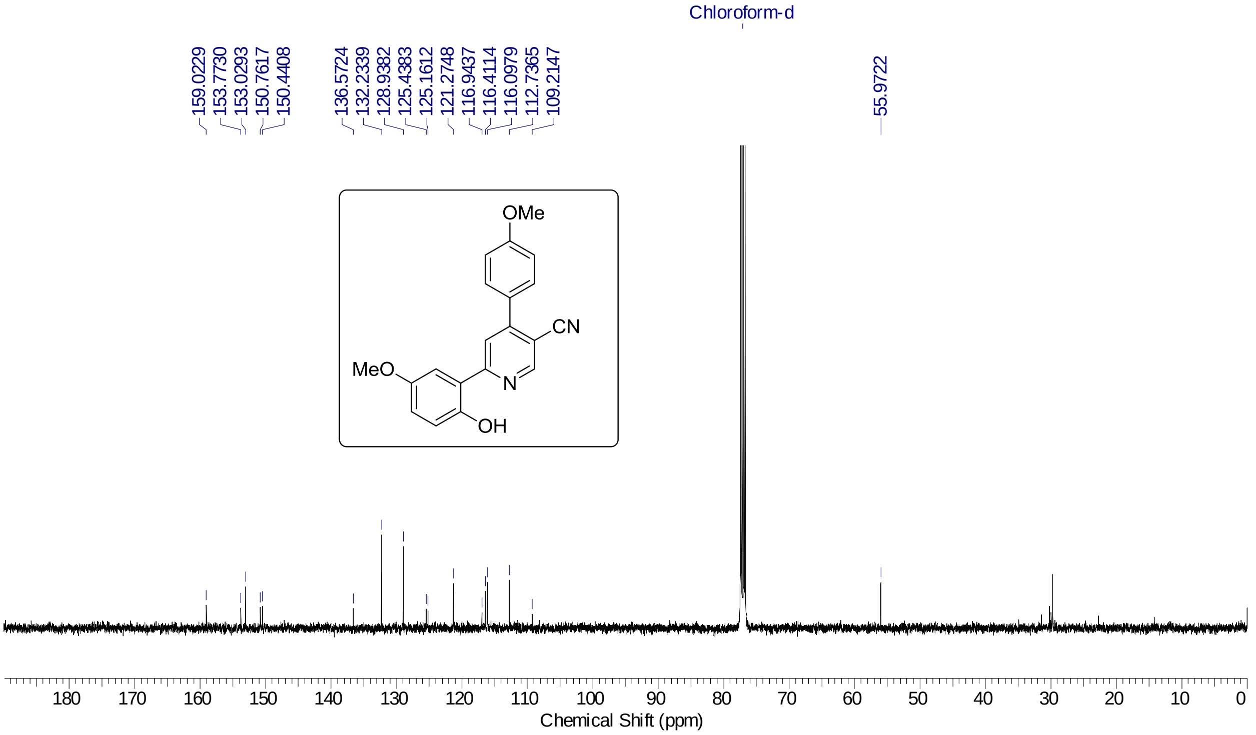
Chloroform-d
159.0229
153.7730
153.0293
150.7617
150.4408
136.5724
132.2339
128.9382
125.4383
125.1612
121.2748
116.9437
116.4114
116.0979
112.7365
109.2147
55.9722
OMe
CN
MeO
N
OH
180 170 160 150 140 130 120 110 100 90 80 70 60 50 40 30 20 10 0
Chemical Shift (ppm)

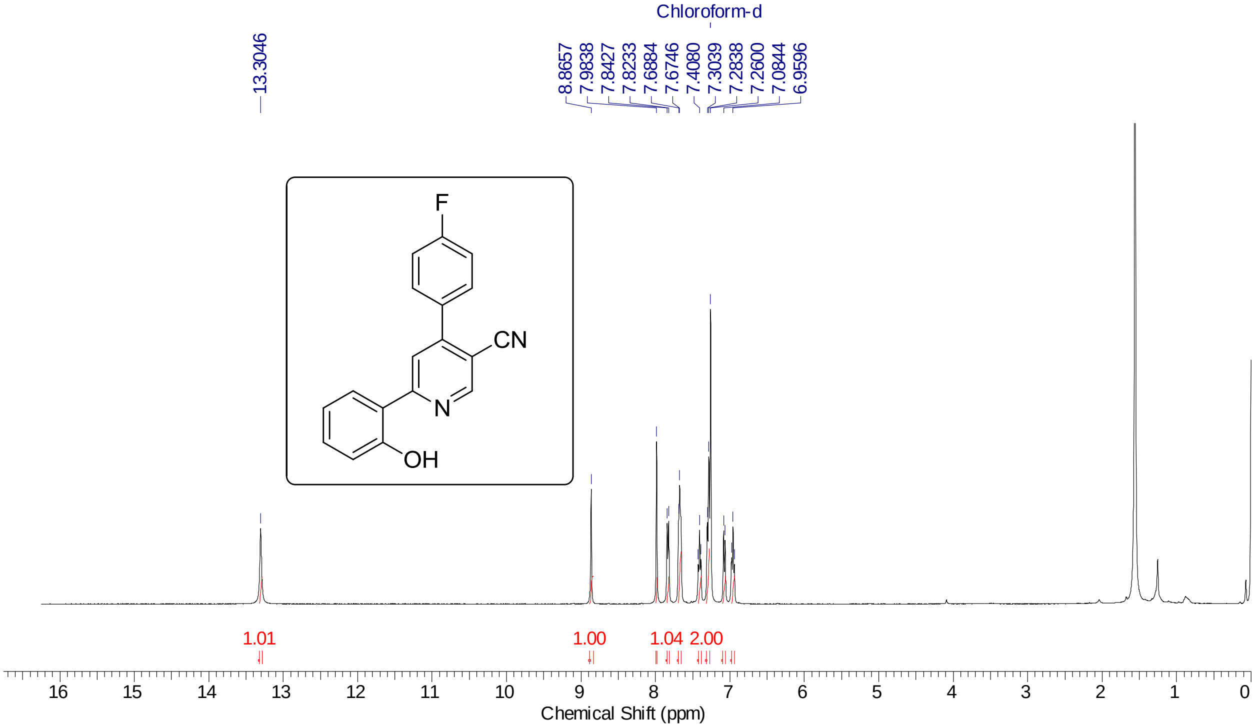
Chloroform-d
13.3046
8.8657
7.9838
7.8427
7.8233
7.6884
7.6746
7.4080
7.3039
7.2838
7.2600
7.0844
6.9596
F
CN
N
OH
1.01
1.00
1.04
2.00
Chemical Shift (ppm)

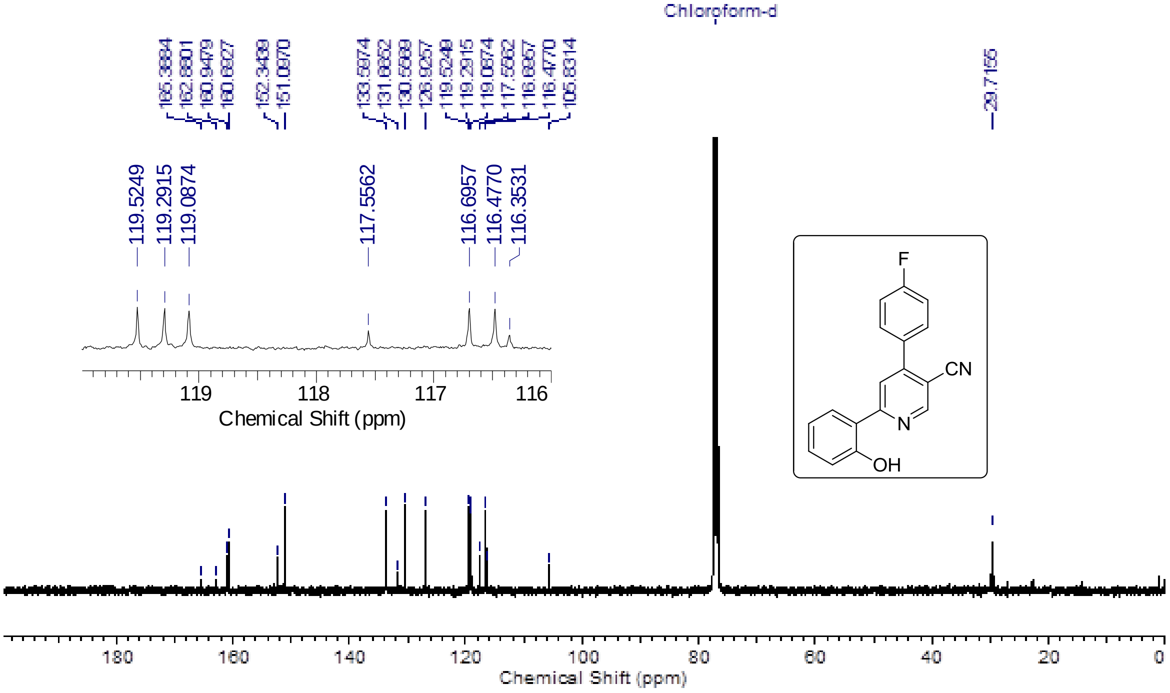
Chloroform-d
165.3884
162.8801
160.9479
160.6927
152.3439
151.0970
133.5974
131.6662
130.5589
126.9257
119.5249
119.2915
119.0874
117.5562
116.6957
116.4770
105.8314
29.7155
119.5249
119.2915
119.0874
117.5562
116.6957
116.4770
116.3531
Chemical Shift (ppm)
F
CN
N
OH
Chemical Shift (ppm)
